# Abnormal Cellular Populations Shape Thymic Epithelial Tumor Heterogeneity and Anti‐Tumor by Blocking Metabolic Interactions in Organoids

**DOI:** 10.1002/advs.202406653

**Published:** 2024-09-11

**Authors:** Xuefei Liu, Changchun Wang, Yueyu Huang, Qiaoli Lv, Chang Yu, Jianghua Ying, Lianhui Duan, Yangzhong Guo, Guanyin Huang, Wenhui Shen, Ming Jiang, Weimin Mao, Zhixiang Zuo, An Zhao

**Affiliations:** ^1^ Zhejiang Cancer Institute Zhejiang Cancer Hospital Hangzhou Institute of Medicine (HIM) Chinese Academy of Sciences Hangzhou Zhejiang 310022 China; ^2^ Department of Biochemistry School of Medicine Southern University of Science and Technology Shenzhen 518055 China; ^3^ Shenzhen Institute of Pediatrics Shenzhen Children's Hospital Shenzhen 518026 China; ^4^ Department of Thoracic Oncology Zhejiang Cancer Hospital Hangzhou Institute of Medicine (HIM) Chinese Academy of Sciences Hangzhou Zhejiang 310022 China; ^5^ Thoracic Oncology Laboratory Jiangxi Cancer Hospital Nanchang Medical College Nanchang Jiangxi 330029 China; ^6^ Department of Pathology Zhejiang Cancer Hospital Hangzhou Institute of Medicine (HIM) Chinese Academy of Sciences Hangzhou Zhejiang 310022 China; ^7^ Department of Ultrasound Zhejiang Cancer Hospital Hangzhou Institute of Medicine (HIM) Chinese Academy of Sciences Hangzhou Zhejiang 310022 China; ^8^ Center for Genetic Medicine The Fourth Affiliated Hospital Zhejiang University School of Medicine Hangzhou Zhejiang 310011 China; ^9^ Zhejiang Provincial Key Laboratory of Genetic & Developmental Disorders Hangzhou Zhejiang 310011 China; ^10^ Zhejiang Provincial Key Laboratory of Diagnosis and Treatment of Thoracic Cancer Zhejiang Cancer Hospital Hangzhou Institute of Medicine (HIM) Chinese Academy of Sciences Hangzhou Zhejiang 310022 China; ^11^ State Key Laboratory of Oncology in South China Collaborative Innovation Center for Cancer Medicine Sun Yat‐sen University Cancer Center Guangzhou 510308 China

**Keywords:** histopathology, ionocyte, KRT14^+^ progenitor cells, metabolic interaction, thymic epithelial tumors

## Abstract

A variety of abnormal epithelial cells and immature and mature immune cells in thymic epithelial tumors (TETs) affect histopathological features, the degree of malignancy, and the response to treatment. Here, gene expression, trajectory inference, and T cell antigen receptor (TCR)‐based lineage tracking are profiled in TETs at single‐cell resolution. An original subpopulation of KRT14^+^ progenitor cells with a spindle cell phenotype is shown. An abnormal infiltration of immature T cells with a TCR hyper‐rearrangement state is revealed, due to the lack of CCL21^+^ medullary epithelial cells. For thymic carcinoma, the novel biomarkers of MSLN, CCL20, and SLC1A5 are identified and observed an elevated expression of LAG3 and HAVCR2 in malignant tumorn‐infiltrating mature T cells. These common features based on the single‐cell populations may inform pathological reclassification of TETs. Meanwhile, it is found that macrophages (MACs) attract thymic tumor cells through the LGALS9‐SLC1A5 axis, providing them with glutamine to elicit metabolic reprogramming. This MAC‐based metabolic pattern can promote malignancy progression. Additionally, an interactive immune environment in TETs is identified that correlates with the infiltration of abnormal FOXI1^+^ CFTR^−^ ionocytes. Collectively, the data broaden the knowledge of TET cellular ecosystems, providing a basis for tackling histopathological diagnosis and related treatment.

## Graphical abstract

A variety of abnormal epithelial cells and immature and mature immune cells in thymic epithelial tumors (TETs) affect histopathological features. Through single‐cell resolution perspective and in vitro organoid analysis, the TETs cell ecosystem is deciphered, which provides a basis for tackling histopathological diagnosis and related treatment.

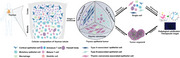

## 1 Introduction

The histological phenotypes and molecular characteristics of tumors inform precise treatment options.^[^ ^1^ ^]^ Currently, the classification of thymic epithelial tumors (TETs) is based on a morphology of spindle or polygonal epithelial cells, and subcategorized based on the proportion of immature lymphocytes in the tumor; however, the rate of pathological inconsistency remains at ≈30–40%.^[^ ^2^ ^]^ Importantly, low‐risk thymoma (TM) can also recur and transform into thymic carcinoma (TC).^[^ ^3^ ^]^ Another phenomenon unique to TETs is the presence of numerous immature lymphocytes accompanying the abnormal epithelial cells, possibly due to an autoimmunity effect.^[^ ^4^ ^]^ Thymic degeneration, clonal evolution of abnormal cells, and immune cell infiltration of the tumor microenvironment are responsible for the diversity of thymic epithelial tumor (TET) phenotypes.

**Figure 1:**
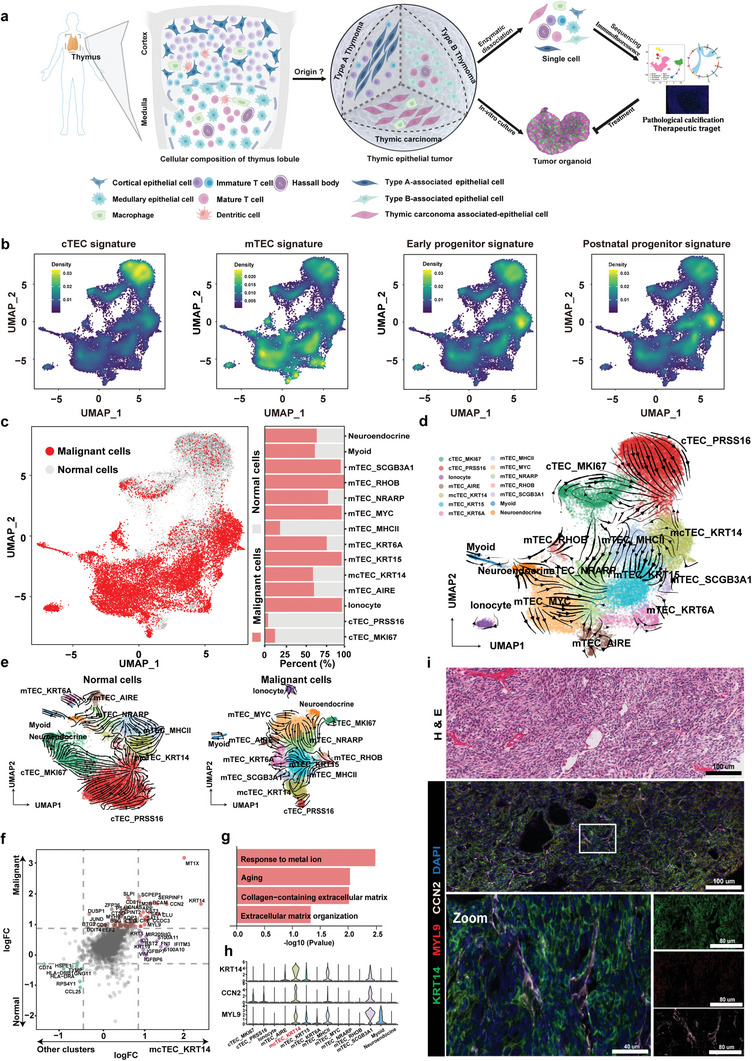
KRT14^+^ spindle cells are progenitor cells of origin in TETs. a) Schematic illustration of sample collection, processing, and single‐cell analysis. b) UMAP plots presenting the aggregated expression profiles of gene groups distinguishing and cTECs, mTEC, early progenitors, and postnatal progenitors. c, UMAP plot showing malignant and normal cells, as defined by inferCNV (left panel), and the relative proportions of malignant or normal cells across the indicated subpopulations (right panel). d‐e) UMAP plot showing the RNA velocity of 14 epithelial cell subtypes in all cells (d), and normal cells and malignant cells (e). f) Scatter plot of the differentially expressed genes from the mcTEC_KRT14 cluster and the other clusters, or malignant cells and normal cells. The genes upregulated in the mcTEC_KRT14 cluster and malignant cells (red) and downregulated in the mcTEC_KRT14 cluster and malignant cells (green) are highlighted. Genes log2FC > 0.58 or < −0.58 were considered significant. g) Bar graph showing the pathway enriched in the mcTEC_KRT14 cluster. The x‐axis indicates the ‐log_10_ P value. h) Violin plot showing KRT14, CCN2, and MYL9 expression in each epithelial cell subpopulation. i) H&E stained tissue sections showing the morphology of KRT14^+^ cells, mIF analysis of frozen TM tissue with antibodies against KRT14 (green), MYL9 (red), CCN2 (white), and DAPI (nuclei; blue), Scale bars: 100 µm; inset, 40 µm.

Single‐cell high‐throughput omics technology has accelerated our understanding of the heterogeneity in the tumor microenvironment, enabling diagnostic and therapeutic information to be obtained from the dominant cell subsets and their role in cell‐cell communication.^[^ ^5^, ^6^ ^]^ More recently, in addition to the traditional consideration that thymic cortical and medullary epithelial cells are the main cells in the thymus, unique progenitor cells, tuft cells, and ionocytes have been discovered in the thymic single‐cell atlas.^[^ ^7^, ^8^, ^9^, ^10^ ^]^ Although several biomarkers have been reported to be associated with prognosis, the origins and molecular characteristics of morphologically distinct malignant cells from different TET subtypes remain unknown.^[^ ^11^, ^12^ ^]^ Here, we applied single‐cell RNA sequencing (scRNA‐seq), single‐cell T cell receptor sequencing (scTCR‐seq), multi‐immunofluorescence (mIF), and in‐vitro organoid culture to elucidate the cellular composition characteristics of different TET subtypes, enabling a refined histopathological classification and treatment strategy based on clinical tissue samples (**Figure** **1**a).

## 2 Results

### 2.1 Characterization of Cellular Composition in TETs

We conducted single‐cell transcriptomic profiling of normal thymus tissues from 7 individuals and 13 TETs cases diagnosed with type A TM, type AB TM, type B1 TM, type B2 type B2 TM, type B3 TM, TM with lymphoid stroma (MNT), metaplastic thymoma (MT), and TC (Table S1, Supporting Information). After combining all scRNA‐seq data, quality control, and batch correction, 159607 cells were detected, with a median of 1983 genes per cell, in which 53311 and 106296 cells were collected from normal and tumor samples, respectively. Using the classical features of different cell clusters, we identified 8 cell types, including B cells (3698; *CD19* and *MS4A1*), endothelial cells (4478; *PECAM1* and *VWF*), epithelial cells (27 343; *KRT8* and *KRT17*), fibroblast cells (3882; *COL1A1* and *COL6A1*), mast cells (7998; *CPA3* and *MS4A2*), myeloid cells (7871; *CD14* and *FCGR3A*), plasma cells (1322; *TNFRSF17* and *IGHG1*), and T cells (109 731, *CD3D* and *CD3E*) (Figure S1a‐b, Supporting Information). Numerous epithelial cells were detected in both normal thymic tissues and TC tissues, however, the proportion of T cells was significantly greater than that of epithelial cells in patients with type B TM (Figure S1c, Supporting Information).

We subclassified the thymic epithelial cells (TECs) expressing *EPCAM* and *KRT8* and found 14 epithelial subpopulations (Figure S2a, Supporting Information). Among the 14 subpopulations, we identified cortical TECs (cTECs) highly expressing *PRSS16* and *PSMB11*, pan‐medullary epithelial cells (mTECs) highly expressing *CLDN3* and *CLDN4*, mature medullary epithelial cells highly expressing *AIRE*, and an intermediate population between cTECs and mTECs (mcTECs) highly expressing *CCL19* and *KRT14*. Three rare epithelial cell types, namely CHGA^+^ neuroendocrine cells, FOXI1^+^ ionocytes, and MYOG^+^ myoid cells, were also clustered (Figure S2a‐c, Supporting Information). One proliferative cTEC and one proliferative mTEC cluster with high cell cycle scores were detected (Figure S2d, Supporting Information). PRSS16^+^ cTECs were enriched in normal thymus tissues and KRT14^+^ mcTECs were enriched in TM. Ionocytes were primarily present in type A and AB TM (MA1 and MA2), while MYC^+^ mTECs were mainly enriched in TC (CA1 and CA2) (Figure S2e, Supporting Information). Two clusters of cTECs had similar expression profiles, and the MHCII^+^ mTEC, KRT14^+^ mcTECs, and SCGB3A1^+^ mTECs had similar expression profiles, while the other more mature mTECs clustered separately (Figure S2e, Supporting Information).

### 2.2 KRT14^+^ Spindle Cells were Identified as Progenitor Cells of Origin for TETs

Using the known signature of the thymus,^[^ ^13^ ^]^ we identified four major epithelial cell states during thymic development (Figure 1b). Interestingly, the KRT14^+^ mcTEC subpopulation had the highest early progenitor signature score and postnatal progenitor signature score among all epithelial cells. We inferred copy‐number variation (CNV) from single‐cell transcriptomes and detected the canonical CNV (e.g., chromosome 6 deletion; Figure S2f, Supporting Information). Malignant and normal TECs were distinguished by CNV (Figure 1c). Malignant TECs were less prevalent in the cTECs, as they were mainly derived from normal tissue. However, in ionocytes, KRT15^+^, KRT6A^+^, MYC^+^, NRANP^+^, RHOB^+^, and SCGB3A1^+^ mTEC subpopulations, malignant cells accounted for over 75% of the cells (Figure 1c). We next performed whole exome sequencing (WES) on four patients, MA2, MA3, MA7, and M2. Although patients MA2 and MA3 were diagnosed with type A or AB TM, we did not identify the reported mutation (Table S2, Supporting Information), *GTF2I*, which is commonly found in type A and AB TM.^[^ ^14^ ^]^ However, a significant *HRAS* mutation in the MA2 patient with TM was detected, and the tumor mutational burden (TMB) of these four patients was similar to the TMB of THYM in the TCGA database (Figure S2g‐h, Supporting Information). Additionally, we verified that the mutation sites of patient MA2, as identified by WES, could be mapped to the scRNA‐Seq data (Figure S2i, Supporting Information).

Using RNA velocity or PAGA analysis, regardless of whether we used all cells for clustering or only malignant or normal cells for clustering, we found apparent directional flows and transition probabilities (links) from KRT14^+^ mcTECs to other mTECs or cTECs, indicating these thymic epithelial progenitor cells (TEPCs), like‐KRT14^+^ mcTECs, had the potential to differentiate into other cells (Figure 1d‐e; Figure S3a‐b, Supporting Information). *APP*, *MYL9*, *CCN2*, *CLU*, *CST3*, and *SAA1* were significantly upregulated in KRT14^+^ mcTECs, compared to other TEC subpopulations, and revealed a significant enrichment of pathway in aging, which is congruent with the late‐onset (median age = 57) demographic of this disease^[^ ^15^ ^]^ and collagen‐containing extracellular matrix (Figure 1f‐g). These genes, which exhibit a positive correlation with the *KRT14* expression, potentially interact with each other at the protein level (Figure S3c‐d, Supporting Information).

Notably, the KRT14^+^ mcTECs showed significant upregulation of *CCN2* and *MYL9*, genes closely related to the regulation of cellular fibrosis^[^ ^16^ ^]^ (Figure 1h). In HE‐stained type A TM tissue, epithelial cells with KRT14^+^ expression showed a predominantly spindle morphology and were characterized by sheets of enlarged and hyperchromatic spindle cells with oval nuclei exhibiting prominent nucleoli and scattered mitoses, and the co‐expression of *CCN2*, *MYL9*, and *KRT14* was also identified by mIF (Figure 1i) We also detected the positive expression of MYL9 in tissues from KRT14^+^ patients (MA3 and M2) by IHC, as compared with the negative expression of MYL9 in KRT14‐negative patients (MA7 and CA1) (Figure S3e, Supporting Information). These results indicate that KRT14^+^ mcTECs is one of the typical cell populations in TETs, with unique spindle‐shaped morphology and progenitor‐like molecular characteristics.

### 2.3 Reclassification of TETs based on KRT14^+^ Spindle Cells Phenotype

**Figure 2:**
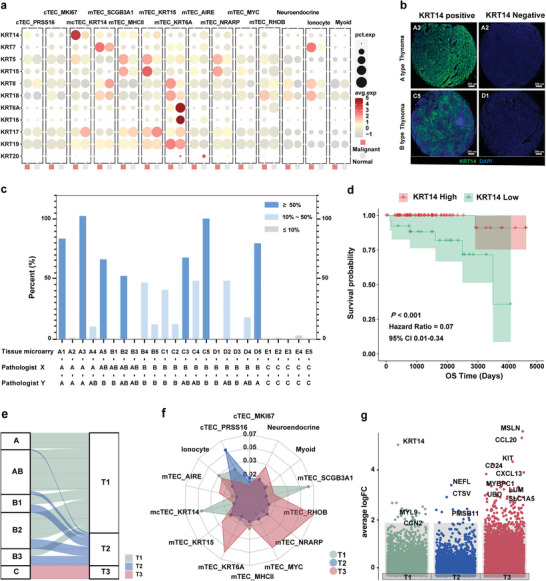
Reclassification of TETs based on KRT14^+^ spindle cell phenotype. a) KRT gene expression levels across epithelial subtypes in normal and malignant cells. Dot size indicates the fraction of expressing cells and the color represents normalized expression levels. b‐c) IHC staining for KRT14 (green) and nuclei (DAPI; blue) in TETs. Scale bars: 200 µm (b). Bar graph showing the percentage of KRT14‐positive cells in 25 patients (c). d) Kaplan‐Meier estimation of overall survival time in TM according to the expression level of KRT14. HRs and 95% CIs were calculated based on a Cox regression model (two‐sided P value by log‐rank test). e) Sankey plot showing the representative TCGA data from TETs divided into three subtypes T1 (KRT14^+^), T2 (KRT14^−^), and T3 (TC). f) Radar plot showing the infiltration score for each cluster of each TET subtype. Infiltration scores were calculated using MCP_Counter with each cluster's differentially expressed genes (logFC > 0.58 and *P* < 0.05). g) Representative upregulated genes in each subtype (T1, T2, and T3) based on DEG analysis (logFC > 0.58 and *P* < 0.05) in TCGA. P values were calculated using a two‐sided paired Wilcoxon signed‐rank test.

To further evaluate the uniqueness of the KRT14^+^ spindle cell phenotype in TETs, we tested all expressed cytokeratins in malignant and normal TEC subpopulations. KRT6A and KRT17, commonly used markers for the detection of thymoma, were not significantly overexpressed in the malignant cell populations; Only KRT14 was found overexpressed in the malignant mcTEC subpopulation (**Figure** **2**a). We next evaluated the expression of KRT14 in TET tissue microarrays (TMA) containing 20 TM and 5 TC tissue spots (Figure S4a and Table S3, Supporting Information). KRT14 was not expressed in tumors with TC, but in both type A and type B TM, there was a strong positive expression (>50%) in 35% of cases (7 out of 20), and a positive expression (>10%) in 75% of cases (Figure 2b‐c; Figure S4b, Supporting Information). In the TCGA‐THYM dataset, the overall survival (OS) and progression‐free intervalsurvival (PFI) of KRT14 high‐expression patients were significantly better survival than those of KRT14 low‐expression patients (Figure 2d; Figure S4c, Supporting Information).

We next categorized all patients from the The Cancer Genome Atlas （TCGA) dataset diagnosed with TETs into three subtypes. The T1 subtype represented TM patients with high KRT14 expression, T2 subtype represented TM patients with low KRT14 expression, and T3 represented patients with TCs (Table S4, Supporting Information). Many patients with type A and AB TM, and approximately half of the patients with type B TM, were classified into the T1 subtype, while the remaining TM patients were classified into the T2 subtype (Figure 2e). The prognosis of TM patients in the T2 and T3 subtypes was similar, indicating that TM patients with low KRT14 expression may have a poor prognostic risk (Figure S4d, Supporting Information). The above finding suggests that KRT14 may serve as a biomarker of the spindle cell population and is positively correlated with a better prognosis of TM.^[^ ^17^, ^18^ ^]^

To gain additional molecular regulatory information on aberrant KRT14^+^ mcTECs, we found the *NFIX*, *GATA2*, *TBX1*, *ZNF560*, and *ZNF208* transcription factors (TFs) to be upregulated (Figure S5a, Supporting Information) and identified their regulatory network (Figure S5b, Supporting Information). We examined these overexpressed TFs in the extended bulk expression dataset to find that *TBX1* showed the highest shift in expression (Figure S5c, Supporting Information). Of note, the median expression value of *TBX1* was greatest in the KRT14^+^ high TM group compared to the other 32 TCGA pan‐cancer tumor types (Figure S5d, Supporting Information). Interestingly, IHC staining revealed TBX1 especially expressed in the medulla region with Hassall's corpuscles; mIF also showed KRT14^+^ cells surrounding TBX1^+^ cells (Figure S5e, Supporting Information). It has been reported that homozygous TBX1 knockout mice have a phenotype resembling del22q11.2 syndrome, including thymus abnormalities.^[^ ^19^ ^]^ KRT14^+^ cells are not found in the embryonic stage, and differentiation appears in the thymus of children and adults.^[^ ^20^, ^21^ ^]^ These findings suggest that TM with the KRT14^+^ mcTEC phenotype may be a benign proliferative state of progenitor cells.

We further investigated the molecular characteristics of T1, T2, and T3 subtypes. Deconvolution analysis was used to compare the distribution of each cell population across the three subtypes, we found that the KRT14^+^ mcTEC was significantly assembled in the T1 subtype, while the PRSS16^+^ cTEC was significantly assembled in the T2 subtype, and MYC^+^ mTEC was significantly assembled in the T3 subtype (Figure 2f). In the bulk‐RNA sequencing data, several cTEC markers, such as *CTSV* and *PSMB11*, were also identified in the T2 subtype; these markers were overlapped in the PRSS16^+^ cTEC (Figure 2g; Figure S6a and Table S5, Supporting Information). In addition, 4 selected cTEC signatures (*LY75*, *CTSV*, *CXCL12*, and *PSMB11*) were confirmed to be overexpressed in the T2 subtype and cTEC populations (Figure S6b‐d, Supporting Information). Pathway analysis also showed that there were significant differences in WNT, NFkB, and TNFa signaling pathways among the TEC subpopulations and three subtypes (Figure S6e‐f, Supporting Information). Collectively, these results imply that the different subtypes of TETs have distinct characteristics of cell subpopulations, and KRT14 and cTEC‐associated signatures are potential biomarkers for predicting the prognosis of TMs.

### 2.4 Molecular Impacts of TM‐Enriched Immature T Cells

Lymphocyte‐rich B‐like area (B‐like area) is a common histological feature that defines the AB and B types of TM; however, the molecular factors responsible for this unique histology remain unknown. We hypothesized that the abnormal interaction between TEC populations and lymphocytes is the reason for the B‐like area formation.

**Figure 3:**
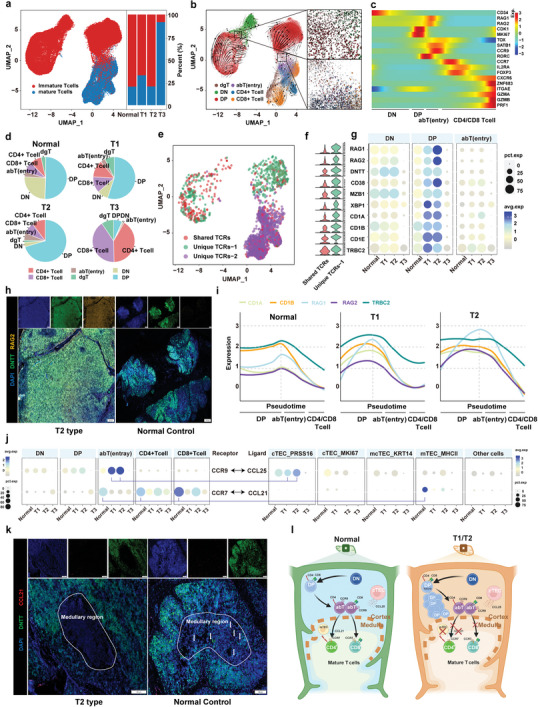
Molecular impact of TM‐enriched immature T cells. a) UMAP of total T cells showing mature and immature T cells (left panel) and bar graph showing the percentage of mature and immature T cells in the normal thymus and the three TET subtypes (right panel). b) UMAP of the RNA velocity of 6 different states of T cells (left panel) and the developmental trajectory from DN to DP and DP to abT cells (right panel). c) Heatmap showing the differentially expressed genes across T cell differentiation pseudotime. The x‐axis represents pseudo‐temporal ordering. The gene expression levels across the pseudotime axis were maximum‐normalized and smoothed. d) Pie charts showing the proportion of the different cell subtypes across normal tissue and the T1, T2, and T3 subtypes. e) UMAP showing the three types of TCR characteristics in T cell development. Shared TCRs represent TCRs shared between immature and mature cells. Unique TCRs‐1 represent TCRs only found in immature cells. Unique TCRs‐2 represent TCRs only found in mature cells. f) Violin plots showing the ten most upregulated genes in the unique‐TCRs‐1 clusters compared to shared‐TCRs. g) Expression levels of the genes presented in panel f across the DN, DP, and abT clusters in patients with normal tissue and the three TET subtypes. Dot size indicates the fraction of expressing cells and the color reflects the normalized expression levels. h) mIF staining images showing DNTT (green), RAG2 (yellow), and DAPI (nuclei; blue) in normal human thymus and T2 TETs. Scale bars, 200 µm. i) Line plots showing the expression of TCR rearrangement molecules (CD1A, CD1B, RAG1, RAG2, and TRBC2) along pseudotime in normal tissue and the T1 and T2 subtypes. Each gene is depicted using a different color line. j) CCR9 and CCR7 expression levels across 5 T cell subpopulations in patients with normal tissue and the three TET subtypes (left panel). CCL25 and CCL21 expression levels across 5 epithelial subpopulations in patients with normal tissue and the three TET subtypes (right panel). Each line represents potential interactions between cells. k) mIF staining images showing DNTT (green), CCL21 (red), and DAPI (nuclei; blue) in the normal human thymus and T2 TET samples (Scale bars, 200 µm). White circles indicate the medullary region structure (Scale bars, 100 µm). l) Schematic illustration of T cell migration from the cortical region to the medullary region in the T1 and T2 subtypes.

We reclassified the enrolled patients by Jaccard correlation analysis on the single‐cell expression data: 7 patients (MA1–MA5 and M1–M2) as the T1 subtype, 4 patients (MA6–MA9) as the T2 subtype and 2 patients (CA1 and CA2) as the T3 subtype (Figure S7a, Supporting Information). In general, lymphocytes were abundant in the normal, T1, and T2 subtypes, with the proportions significantly higher than that in the T3 subtype (Figure S7b, Supporting Information). Mature T cells and immature T cells were distinguished by unsupervised clustering; the lymphocytes of the T3 subtype were dominated by mature T cells, whereas the lymphocytes of the T1 and T2 subtypes were dominated by immature T cells (**Figure** **3**a). Based on classical features, T cells were clustered into 6 developmental stages: double negative (DN) cells (CD3^+^, CD4^−^, and CD8^−^), delta‐gamma T (dgT) cells (TRGC2^+^), double positive (DP) cells (CD3^+^, CD4^+^, and CD8^+^), alpha‐beta T (abT) cells (CD3^+^, TOX^+^, and CCR9^+^), CD4^+^ T cells (CD3^+^, CD4^+^, and CD8^−^), and CD8 cells (CD3^+^, CD4^−^, and CD8^+^; Figure 3b; Figure S7c, Supporting Information). RNA velocity analysis indicated that the flow directions were from DN to DP cells and from DP to abT cells (Figure 3b). We generated a heatmap of hallmark gene expression changes in thymic progenitor cell (CD34^+^), DN and DP cell (RAG1^+^), abT (TOX^+^ and CCR9^+^) and mature T cell (GZMB^+^ and PRF1^+^) stages (Figure 3c). Significant enrichment of DP and abT stage T cells was found in patients with the T2 subtype (Figure 3d; Figure S7d, Supporting Information).

Further extracting the single cell TCR information of lymphocytes, no significant bias in the different subtypes was found in the number, specific rearrangement event, or diversity of the TCR (Figure S8a‐b, Supporting Information). The distribution of the number of TCR paired‐lymphocytes also showed significant enrichment of immature T cells in the T1 and T2 subtypes, similar to the above single‐cell expression data (Figure S8c‐d, Supporting Information). We next compared the changes in gene expression of TCR‐paired immature T cells (unique‐TCR‐1; TCRs that only appear in the immature stage) and TCR‐paired normal development T cells (shared‐TCRs that appear in both immature and mature state) that exhibited augmented expression of genes associated with TCR rearrangements, such as *RAG1*, *RAG2* and *DNTT* (Figure 3e‐f; Figures S8e and S9a‐b, Supporting Information). The expression levels of *RAG1* and *RAG2* in the T2 subtype were also significantly upregulated compared to normal tissue and the T3 subtype (Figure 3g). The results of mIF assays indicate that RAG2 was highly expressed in DNTT‐labeled immature T cells from the T2 subtype compared with normal thymus tissues (Figure 3h). Antigen receptor β rearrangement is an event that occurs during the DP stage of T‐cell development. This process involves *TRBC2*, a gene encoding the TCR beta constant two protein.^[^ ^22^ ^]^ We found that the expression level of *TRBC2* was also upregulated in T1 and T2 subtypes compared with normal tissues (Figure 3g). We further evaluated the expression level of *RAG1/2* and *TRBC2* in different developmental stages of immature T cells. Interestingly, the expression changes of TCR rearrangement regulatory genes were coordinated during immature T cell development in the normal thymus, such that *RAG1/2* and *TRBC2* were simultaneously up‐regulated at the late DP stage and down‐regulated at the mature T cell stage (Figure 3i). However, *TRBC2* was not upregulated at the same time as *RAG1/2* at the late DP stage in T2 (Figure 3i). These results suggest that TM‐associated immature T cells are in an abnormal state of TCR rearrangement due to the abnormal expression of *RAG1/2* and *TRBC2*.

Chemokines (CCLs) secreted by TECs interact with chemokine receptors (CCRs) to guide the migration and development of T cells in an orderly manner.^[^ ^23^ ^]^ Evidence of abnormal immature T cells and proliferating TECs led us to hypothesize that these cells may interact. Therefore, we evaluated the correlation of CCL‐CCR ligand‐receptor pairs at the cell cluster level. Among the CCL‐CCR pairs, CCL25‐CCR9 pairs were significantly enriched in T2 subtype patients, whereas the absence of CCL21 expressing mTECs likely hindered CCR7^+^ immature T cell migration from the cortical to medullary regions (Figure 3j). In normal thymic tissues, the MHCII^+^ mTECs exhibited elevated expression of *CCL21* and upregulated human leukocyte antigen (HLA) genes, possibly guiding immature T cell migration to the medulla and negative selection^[^ ^24^ ^]^ (Figure S10a, Supporting Information). CCL21^+^ mTECs were assessed by mIF, exhibiting positive fluorescence in normal thymic medullae but not in T2 thymic medullae (Figure 3k; Figure S10b, Supporting Information). In addition, the expression levels of *RAG1*, *RAG2*, and *DNTT* positively correlated with the expression levels of *CCL25* and *CCR9*, but negatively correlated with the expression levels of *CCL21* in the extended TCGA and Gene Expression Omnibus (GEO) datasets (Figure S10c, Supporting Information). These findings hint that the lack of CCL21^+^ mTECs hinders the migration of immature T cells to the next stage, leading to the accumulation of resident immature T cells at the TCR hyper‐rearrangement state by communicating with CCL25^+^ cTECs, possibly contributing to the formation of the B‐like area in TM (Figure 3l).

### 2.5 Functional Diversity of T3 Subtype Infiltrating Mature T Cells

**Figure 4:**
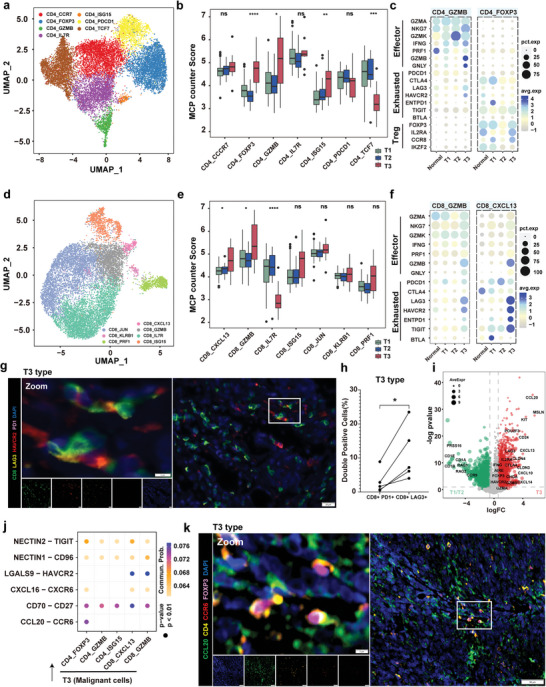
Functional diversity of T3 subtype infiltrating mature T cells. a) UMAP plot showing 7 CD4^+^ subpopulations. b) Box plot showing the infiltration score by MCP_counter of the 7 clusters of CD4^+^ T cells shows different states in the T1, T2, and T3 subtypes. ns, *P* > 0.05; *P < 0.05; **P < 0.01; ***P < 0.001; and ****P < 0.0001, as determined by one‐way ANOVA. c) Dot plots showing the expression levels of Treg, exhausted, and effector T cell marker genes across the CD4_GZMB and CD4_FOXP3 clusters in patients from normal tissues and the three TET subtypes. d) UMAP plot showing 7 CD8^+^ subpopulations. e) Box plot showing the infiltration score by MCP_counter of the 7 clusters of CD8^+^ T cells shows different states in the T1, T2, and T3 subtypes. ns, *P* > 0.05; *P < 0.05; and ****P < 0.0001, as determined by one‐way ANOVA. f) Dot plots showing the expression levels of exhausted and effector T cell marker genes across the CD8_GZMB and CD8_CXCL13 clusters in patients from normal tissue and the three TET subtypes. g) mIF staining images showing CD8 (green), LAG3 (pale yellow), HAVCR2 (red), PD1 (pink), and DAPI (nuclei; blue) in tissue from a T3 subtype patient. Scale bar, 20 µm. h) Quantification of percentage of PD1+ CD8+ T cells and LAG3+ CD8+ T cells in the tissue section of 5 patients with T3 subtype. *P < 0.05; as determined by paired t‐test i) Volcano plots showing the differentially expressed genes between the T1/T2 and T3 subtypes. j) Dot plot showing the indicated ligand‐receptor pairs between malignant cells in the T3 subtype and T cell subpopulations. P values were determined using a one‐sided permutation test. k) mIF staining images showing CCL20 (green), CD4 (yellow), CCR6 (red), FOXP3 (pink), and DAPI (nuclei; blue) in tissue from a T3 subtype patient. Scale bar, 100 µm; inset, 5 µm.

T3 subtype‐related single‐cell populations clearly originated from patients with TC, which distinguishes this group from the T1 and T2 subtypes, and exhibited fewer infiltrating immature T cells and an abundance of infiltrating mature CD4^+^ and CD8^+^ T cells (Figure 2e and Figure 3d; Figure S7d, Supporting Information). Although pembrolizumab has been recommended as a second‐line treatment for TC, immune‐related adverse events occur frequently.^[^ ^25^, ^26^, ^27^ ^]^ To gain additional insight into the role of these mature immune cells in the T3 subtype, we divided purified mature T cells that only expressed CD4^+^ or CD8^+^ cells into subpopulations according to their unique gene expression profiles and mature TCR (TCRs‐2)^[^ ^28^, ^29^ ^]^ (Figure 3e and **Figure** **4**a,d; Figures S7c and S11a,c, Supporting Information). The regulatory FOXP3^+^ CD4 subpopulation and effector GZMB^+^ CD4 subpopulation exhibited significant infiltration in patients with the T3 subtype compared with the T1 and T2 subtypes (Figure 4b). Markers of T cell exhaustion, as well as some effector markers such as GZMA and NKG7, did not differ significantly between the T3 patients and normal controls; however, the expression of GZMB showed a significant increase in the T3 subtype (Figure 4c; Figure S11b, Supporting Information). Among the CD8 subpopulations, the exhausted CXCL13^+^ CD8 subpopulation and the effector GZMB^+^ CD8 subpopulation significantly infiltrated in the T3 subtype (Figure 4). Additionally, in the T3 subtype, *GZMB* together with *LAG3*, *HAVCR2*, *ENTPD1*, and *TIGIT* were overexpressed in these two CD8^+^ subpopulations, while common immune checkpoints inhibitors (ICIs), such as *PDCD1* and *CTLA4*, were not found to be significantly overexpressed (Figure 4f; Figure S11d, Supporting Information). Consistent with the single‐cell expression data, the TCGA‐THYM dataset also indicated that the T3 subtype was infiltrated with T cells overexpressing *GZMB*, *GNLY*, and *IFNG* (Figure S11e, Supporting Information). CD8/PD1/LAG3/HAVCR2 staining underscored that the T3 subtype‐infiltrating CD8^+^ T cells expressed *LAG3* and *HAVCR2*, but not *PD1* (Figure 4g). Moreover, the proportion of LAG3^+^ CD8^+^ T cells in the patient with T3 subtype was significantly higher than that of PD1^+^ CD8^+^ T cells (P < 0.05; Figure 4h)

The biomarkers identified in the T3 subtype include MSLN and CD117 (KIT), which have been reported in TC and other advanced tumor cells,^[^ ^30^, ^31^, ^32^ ^]^ and CCL20 which has not been reported in TC (Figure 4i; Figure S12a, Supporting Information). Based on the above findings of T3 subtyped‐associated malignant cells and infiltrating mature T cell populations, we identified several enriched ligand‐receptor pairs, including NECTIN2‐TIGIT, NECTIN1‐CD96, LGALS9‐HAVCR2, CXCL16‐CXCR6, CD70‐CD27, and CCL20‐CCR6; notably CCL20^+^ malignant cells uniquely recruited CCR6^+^ FOXP3^+^ regulatory T cells, suggesting immune suppression (Figure 4j; Figure S12b, Supporting Information). In the TCGA pan‐cancer datasets, THYM patients with the T3 subtype exhibited the highest expression of *CCL20*, as well as the highest CCL20‐CCR6 ligand‐receptor pair score (Figure S12c, Supporting Information). In addition, among all TET patients, there was a strong positive correlation between the expression level of *CCR6*, the Treg cell infiltration score, and the expression of FOXP3, the key TF of Treg cells (Figure S12d, Supporting Information). Furthermore, mIF assays demonstrated that CCL20^+^ malignant cells were closely connected with CCR6^+^ FOXP3^+^ CD4^+^ Treg cells (Figure 4k). Collectively, these findings reveal that mature T cells are immunosuppressed and exhausted in patients with the T3 subtype. The abundant infiltration of mature T cells and the unique CCL20‐CCR6 pathway provide potential targets for ICI treatment.

### 2.6 A Macrophage‐Based Metabolic Pattern Promotes the T3 Subtype Malignant Phenotype

**Figure 5:**
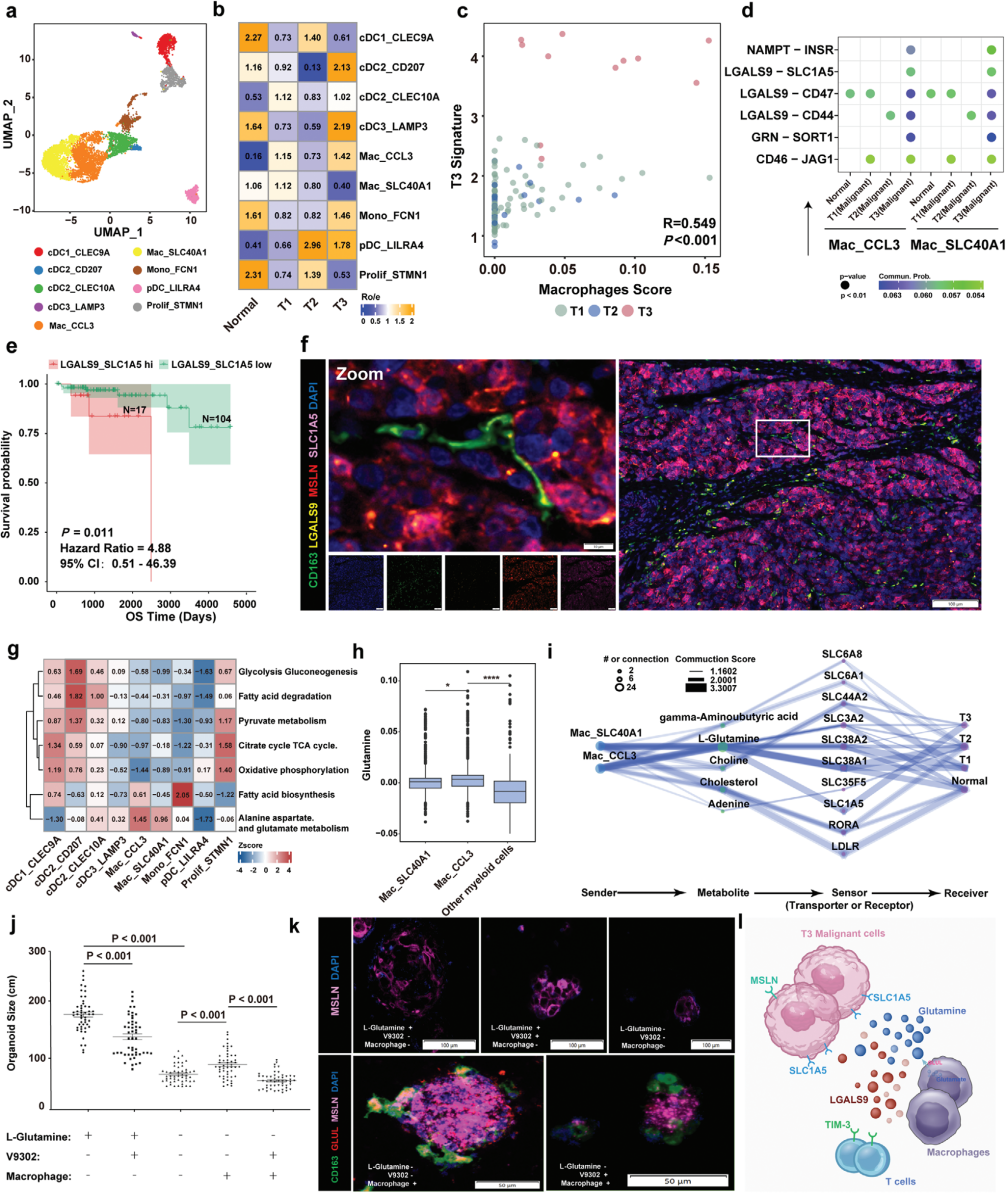
A MAC‐based metabolic pattern promotes the T3 subtype malignant phenotype. a) UMAP plot showing 9 myeloid subpopulations. b) Heatmap showing the subtype distribution of different myeloid cells, as estimated by Ro/e score. c) Scatter plot showing the correlation between the T3 signature score and the MAC score. The T3 signature score was calculated using MCP_counter with the indicated genes (logFC > 0.58 and *P* < 0.05) in the T3 subtype. The MAC score was calculated using MCP_counter with MAC signature genes. The color of the dots represents different subtypes. d) Dot plot showing the selected ligand‐receptor pairs between cells in Mac_CCL3, Mac_SLC40A1, and epithelial cells in the different subtypes. P values were determined using a one‐sided permutation test. e) Kaplan‐Meier estimation of overall survival time in TETs according to the ligand‐receptor pair expression levels of LGALS9_SLC1A5. HRs and 95% CIs were calculated based on a Cox regression model (two‐sided P value by log‐rank test). f) mIF staining images showing CD163 (green), LGALS9 (yellow), MSLN (red), SLC1A5 (pink), and DAPI (nuclei; blue) in tissue from a T3 subtype patient, Scale bar, 100 µm; inset, 10 µm. g) Heatmap showing the metabolic pathways of 9 myeloid cell subtypes in TETs, showing that CCL3^+^ MACs had the highest alanine aspartate and glutamate metabolism metabolic activity. The color intensity represents the scaled metabolic score calculated by GSEA. h) Box plot showing the enrichment level of glutamine in 5 malignant cell subtypes. P values were calculated using a two‐sided paired Wilcoxon signed‐rank test. *P < 0.05 and ****P < 0.0001. i) Diagram showing the information flow of metabolite‐sensor communication from MACs to malignant cells through metabolites and sensors. The dot size represents the number of connections. The lines connect the sender, metabolite, sensor, and receiver. The color of the line indicates the ‐log10 (P value) and the width of line represents the communication score. j) Average circumferences of TET organoids in Extended Data Fig 14c on day 14. Each symbol represents a single organoid. k) mIF staining images showing CD163 (green), GLUL (red), MSLN (pink), and DAPI (nuclei; blue) in organoids. Scale bar, 100 µm. l) Schematic illustration of the cell‐cell communication between MACs and malignant epithelial cells.

Myeloid cells play a central role in regulating the balance between T‐cell immunity and tolerance to tumor antigens.^[^ ^33^ ^]^ We therefore subclustered 7871 myeloid cells and identified nine subpopulations, including five clusters of dendritic cells (DCs), one cluster of monocytes, one cluster of proliferative myeloid cells and two clusters of MACs (**Figure** **5**a, Figure S13a, Supporting Information). Notably, CCL3^+^ MACs and SLC40A1^+^ MACs showed an opposing infiltration relationship between the T3 subtype and the other groups (Figure 5b). Using deconvolution analysis of bulk expression data, we observed a strong positive correlation between the T3 subtype score and the MACs score, with the CCL3^+^ MAC infiltration level increased in the T3 subtype (Figure 5c; Figure S13b, Supporting Information). We next examined the differentially expressed genes between CCL3^+^ MACs and SLC40A1^+^ MACs. Several cytokines associated with cell aging and senescence‐associated secretory phenotype (SASP),^[^ ^34^ ^]^ such as *CXCL8*, *CXCL2*, *TNF*, and *IL1B*, were significantly upregulated in the CCL3^+^ MACs (Figure S13c, Supporting Information).

To further elucidate the impact of MACs on malignant cells, we explored potential ligand‐receptor pairs and found NAMPT‐INSR, LGALS9‐SLC1A5, LGALS9‐CD47, LGALS9‐CD44, GRN‐SORT1, and CD46‐JAG1 are all significantly enriched in cell‐cell communication (Figure 5d; Figure S13d, Supporting Information). Of these, LGALS9‐SLC1A5 has recently been reported to be a stable feature associated with live tumor aggressiveness.^[^ ^35^ ^]^ Therefore, we evaluated the association between the LGALS9‐SLC1A5 interaction score and patient survival time in the TCGA‐THYM dataset. Both the OS and PFI of all TET patients with high scores were significantly lower than those with low scores (Figure 5e; Figure S13e, Supporting Information). The expression levels of *LGALS9* and *SLC1A5* in the T3 subtype were significantly higher than in the T1 and T2 subtypes in the TCGA dataset (Figure S13f, Supporting Information). MSLN was co‐expressed with SLC1A5 in T3 subtype malignant epithelial cells, while infiltrating CD163^+^ MACs positively co‐expressed LGALS9 (Figure 5f). In addition, we also found that CCL3^+^ MACs can recruit exhausted CXCL13^+^ CD8 T cells via the LGALS9‐HAVCR2 ligand‐receptor interaction (Figure S13g, Supporting Information). These findings indicate that CCL3^+^ MACs may play a role in the malignant behavior of TC.

SLC1A5 is known for its role in glutamine transport, but its high expression and its interaction with MACs in TC are still unknown.^[^ ^36^, ^37^ ^]^ Interestingly, fatty acid synthesis, and alanine, aspartate, and glutamate metabolism were abnormally hyper‐activated in CCL3^+^ MACs (Figure 5g). The expression levels of *GLUL*, which encodes an enzyme that converts glutamate to glutamine and glutamine metabolites, were significantly upregulated in CCL3^+^ MACs (Figure 5h; Figure S14a, Supporting Information). Using MEBOCOST to examine the metabolite‐sensor communications, we found that among all metabolite‐sensor partners between MACs and malignant cells, the communication scores of L‐glutamine and its transporter, *SLC1A5*, in the T3 subtype were substantially higher than in the T1 and T2 subtypes (Figure 5i; Figure S14b, Supporting Information).

We next established an in‐vitro TET organoid model, in which the median size of TC‐derived organoids cultured in the presence of glutamine and MACs was significantly greater than the other groups. When glutamine was absent, the median size of MACs‐depleted organoids was significantly lower than the MACs‐supplemented group (Figure 5j‐k; Figure S14c, Supporting Information). Moreover, in the presence of MACs and without glutamine, the median size of organoids treated with V‐9302, an inhibitor of SLC1A5, was significantly lower than that of the untreated group. The GLUL assay was positive when organoids were co‐cultured with MACs under glutamine‐depleted culture conditions (Figure 5j‐k; Figure S14c, Supporting Information). In addition, we also detected a positive correlation between LGALS9‐SLC1A5 ligand‐receptor interaction and alanine, aspartate, and glutamate metabolism pattern by the RNA‐seq data in TCGA‐THYM database (Figure S14d, Supporting Information). These findings suggest glutamine produced by MACs did not support organoid growth when SLC1A5 was inhibited, and this MAC‐based metabolic pattern can promote the malignant progression of thymic carcinoma cells (Figure 5l).

### 2.7 Identification of a Rare Abnormal FOXI1^+^ CFTR^−^ Ionocytes in TETs

**Figure 6:**
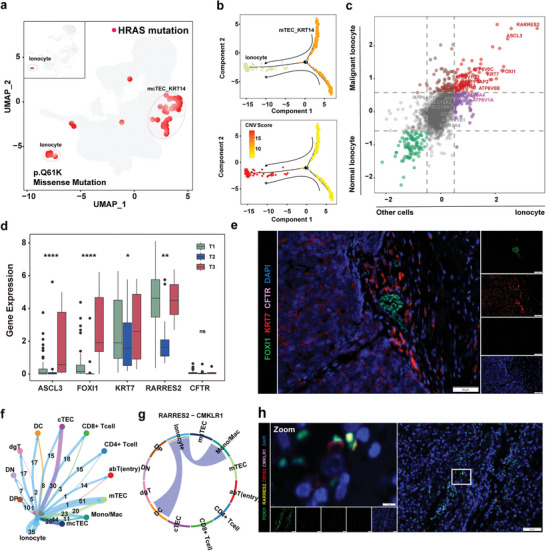
Identification of a rare abnormal FOXI1^+^ CFTR^−^ ionocyte in TETs. a) UMAP showing all cells with the HRAS missense mutation (p.Q61K) and highlighting the cells defined as ionocytes. b) Pseudotime‐ordered analysis of cells in the mcTEC_KRT14 cluster and ionocytes, as inferred by Monocle2. Cell subtypes are labeled in different colors (top) and pseudotime (bottom). c) Scatter plot of the differentially expressed genes from the ionocyte cluster and the other clusters, or malignant ionocyte cells and normal ionocyte cells. The genes upregulated in the ionocyte cluster and malignant ionocyte cells (red) and downregulated in the ionocyte cluster and malignant ionocyte cells (green) are highlighted. Genes log2FC > 0.58 or < −0.58 were considered significant. d) Box plots showing the expression levels of ASCL3, FOXI1, KRT7, RARRES2, and CFTR in T1, T2, and T3 subtype patients. ns, *P* > 0.05; *P < 0.05; **P < 0.01; ***P < 0.001; and ****P < 0.0001, as determined using a one‐way ANOVA test. e) mIF staining images showing FOXI1 (green), KRT7 (red), CFTR (pink), and DAPI (nuclei; blue) in the TET tissues (scale bar, 50 µm). f) Network of crosstalk between ionocytes and other cell types. g) Circos plot showing the RARRES2‐CMKLR1 interactions among the indicated cell types. The strings are directional and represent interactions determined based on the expression of RARRES2 by one cell type and expression of CMKLR1 by another cell type. The thickness of each string corresponds to the amount of the interaction pairs. h) mIF staining images showing FOXI1 (green), RARRES2 (pale yellow), CD163 (red), CMKLR1 (pink), and DAPI (nuclei; blue) in the indicated TET tissues (scale bar, 50 µm).

The ionocyte was recently described as a novel type of epithelial cell, identified in the single‐cell atlas of normal renal, lung, and thymus tissues.^[^ ^38^, ^39^ ^]^ Here, we identified a rare ionocyte population carrying the patient MA2 and KRT14^+^ mcTEC‐deveried HARS mutations (**Figure** **6**a). The CNV score of ionocytes was greater than that of KRT14^+^ mcTECs, and the two subpopulations exhibit a close evolutionary relationship (Figure 6b). Analysis of the differentially expressed genes between ionocytes and other epithelial cells subtypes revealed a significant upregulation of *KRT7*, *FOXI1*, *ASCL3*, *RARRES2*, and *TMEM61* in ionocytes (Figure 6c; Figure S15a‐b, Supporting Information). Of note, the major TF in normal ionocytes,^[^ ^38^ ^]^ *CFTR*, exhibited low expression in TET‐associated ionocytes. We validated the biomarkers associated with ionocytes, such as ASCL3, FOXI1, KRT7, RARRES2, and CFTR, in both bulk expression data and by fluorescent IHC staining (Figure 6d‐e). And we also found *ASCL3*, *FOXI1*, *KRT7*, *RARRES2* upregulated in malignant ionocytes (Figure S15c, Supporting Information). These verified ionocytes exhibited an enrichment of the proton‐secreting V‐ATPase, sensory perception of chemical stimulus, and monovalent inorganic cation homeostasis pathways (Figure S15d‐e, Supporting Information). The solute‐linked cotransporter, *SLC4A11*, is a widely known H^+^ transporter^[^ ^40^ ^]^ that was significantly upregulated in ionocytes (Figure S15f, Supporting Information). Members of the branchial Cl^−^ SLC26 anion transporter gene family were not upregulated in ionocytes; however, we did observe upregulation of some members of the SLC12 family of electroneutral cation‐coupled chloride cotransporters, such as *SLC12A2*, *SLC12A4*, *SLC12A7*, in ionocytes (Figure S15g‐h, Supporting Information). The enrichment of these unique ion channels is speculated to be involved in shaping the tumor microenvironment.

Among all identified cell subpopulations in TETs, abnormal ionocytes exhibit a wide range of cross‐talk ability with other cell subpopulations (Figure 6f). Notably, one of the most significantly upregulated genes of ionocytes, RARRES2, can interact with CMKLR1 expressed in MACs (Figure 6g). The CMKLR1‐RARRES2 ligand‐receptor pair has been reported to significantly influence the polarization of MACs.^[^ ^41^ ^]^ CMKLR1 was also only specifically expressed on MACs in TETs (Figure S15i, Supporting Information). FOXI1/RARESS2/CD163/CMKLR1 staining revealed that FOXI1^+^ RATESS2^+^ ionocytes are intimately associated with CD163^+^ CMKLR1^+^ MACs (Figure 6h). Overall, these data are suggestive of an interactive immune environment in TETs that correlates with abnormal FOXI1^+^ CFTR^−^ ionocyte infiltration.

## 3 Discussion

The heterogeneity and pathological characteristics of rare tumors are major challenges in oncology. Constructing a three‐dimensional view from tens of thousands of single‐cell sequencing data from tumors has facilitated identifying changes in aberrant cell subpopulations during cancer occurrence, progression, and drug resistance.^[^ ^42^, ^43^ ^]^ Here, we applied the single‐cell atlas strategy to characterize TETs with different pathological types. Our analysis highlights the complex cellular ecosystem that shapes the unique environment of TETs and an in‐vivo aberrant MAC metabolic cross‐talk program associated with malignant progression. Our study provides a paradigm for understanding intra‐tumor heterogeneity and treatment strategies in TETs. This approach is applicable to other rare malignancies.

Among our key findings, a KRT14^+^ mcTEC subpopulation is a key feature of TM, and is distributed in different proportions among the various subtypes of TM. This cell subpopulation highly expresses myoid‐associated genes, such as MYL9, FN1, and related TFs, which is consistent with the cell morphology of a myoid spindle shape.^[^ ^44^, ^45^, ^46^ ^]^ Moreover, we characterized the expression of KRTs in malignant and non‐malignant thymocyte subpopulations to find KRT14 as the most specific keratin in the abnormal epithelial cells of TM. KRT14^+^ mcTECs were not seen in the embryonic thymus.^[^ ^47^ ^]^ Prior studies have linked the evolutionary relationship of progenitor cell subpopulations and medullary myoid cell subpopulations in the normal thymus, raising the possibility that a program error in the differentiation of the progenitor cells is the cause of TM with a KRT14^+^ mcTEC phenotype.^[^ ^47^, ^48^ ^]^

A lymphocyte‐rich B‐like area (B‐like area) is a histopathological feature of TM, and is also the main factor confusing classification.^[^ ^49^ ^]^ Since the KRT14^+^ spindle cell is the typical phenotype of TM, we divided TETs into T1 (KRT14^+^), T2 (KRT14^−^), and T3 subtypes. The single‐cell molecular profile of immature lymphocytes in each subtype revealed some novel insights into this phenomenon. First, a large proportion of immature T cells was present in the T1 subtype, where the B‐like areas also exhibited expression of KRT14, suggesting that the AB‐ and A‐type TMs may be the same subtype.^[^ ^18^ ^]^ Second, the T2 subtype, with a greater abundance of immature lymphocytes, was accompanied by the PRSS16^+^ cTEC subpopulation; however, no significant abnormal CNV was found in this cell subpopulation. Third, immature lymphocytes in the T1 and T2 subtypes exhibited abnormal TCR hyper‐rearrangement status and programmed development errors. Notably, the loss of medullary CCL21^+^ cell function in the T2 subtype let to a lack of guidance of DP or abT cells into the medullary region for maturation.^[^ ^50^ ^]^ This underlie the imbalance between thymic cortical and medullary degeneration in TM with a lymphocyte‐rich phenotype.

The cell subpopulation composition of the T3 subtype was significantly different from that of the T1 and T2 subtypes, showing remarkably greater CNV levels, and specific markers, such as MSLN, which was recently reported as a potential target for CAR‐T treatment^[^ ^51^ ^]^ Additionally, SLC1A5, a glutamine transporter, was first observed to be highly expressed in the malignant epithelial cell population of TC. Another intriguing finding in our study is that T3 subtype‐associated MACs can communicate with malignant cells through the LGALS9‐SLC1A5 ligand‐receptor interaction. Although the existence and importance of glutamine in TETs has been described elsewhere, the production of glutamine in the local tumor microenvironment is not yet clear. Using a predictive metabolite‐sensor algorithm and in‐vitro organoid culture, our findings identify a basic mechanism whereby a metabolic pattern of tumor proliferation for glutamine is supplied by MACs. It has also been hypothesized that the LGALS9/TIM‐3 signaling axis can protect cancer from cytotoxic killing by T cells.^[^ ^52^ ^]^ It remains unclear whether a key role for MACs exists in TC, or if the spectrum extends only to the polarization state. Regardless, we defined a specific MAC‐based‐metabolic pattern in cancer patients that can guide future studies of this process in designing clinical trials or in combination with immunotherapy.

A rare abnormal ionocyte subpopulation co‐expressing KRT7, FOXI1, ASCL3, and multiple subunits of V‐ATPase, but low‐expression of CFTR, was identified in our single‐cell atlas and extended bulk RNA‐seq dataset from TETs. The common mutation and evolutionary relationship suggest that these ionocytes originated from the KRT14^+^ mcTEC subpopulation. Several observations indicate that CFTR‐rich ionocytes play a role in luminal pH regulation during airway epithelial development.^[^ ^38^, ^39^, ^53^ ^]^ Interestingly, these CFTR‐poor malignant ionocytes significantly upregulated the expression of SLC4A11, which is an electrogenic H transporter activated by NH_3_ and alkaline pH.^[^ ^40^, ^54^, ^55^ ^]^ Interestingly, glutamine was found to be highly expressed in TC‐associated MACs, where it is an important donor of NH3.^[^ ^55^ ^]^ Furthermore, the RARRES2‐CMKLR1 ligand‐receptor pair was identified to mediate the connection between ionocytes and MACs. These findings suggested a potential function of “energy dialogue” between ionocytes and MACs via glutamine metabolism.

In summary, our study provides important insights into TET biology, while our single‐cell data analysis of malignant, immune, and ionocyte cells complements the overall atlas of these tumors. From the perspective of single‐cell composition and bulk expression data, we describe KRT14^+^ cell‐based TET subtypes and the molecular impacts of the immature/mature T cell‐accompanied TET subtypes. Finally, the in‐vivo MACs‐based‐metabolic pattern may be a more general mechanism of tumor adaptation to the environment. Although further studies of the proposed “energy dialogue” are warranted, blocking the MAC‐based metabolic pattern may present a novel strategy for the treatment of thymic tumors.

## 4 Experimental Section

### Data Availability

The scRNA‐seq, TCR sequencing, and WES data generated in the present study have been deposited in the Genome Sequence Archive in BIG Data Center, Beijing Institute of Genomics, Chinese Academy of Sciences under accession number HRA004536 (publicly accessible at https://ngdc.cncb.ac.cn/gsa‐human). The public scRNA‐seq datasets used in this study are available in the Genome Sequence Archive under the accession number HRA002334. For analysis of the normal thymus, Jong‐Eun Park et al.’s dataset was downloaded from ArrayExpress (accession number E‐MTAB‐8581)^[^ ^7^ ^]^ and Jhoanne L. Bautista et al.’s dataset was downloaded from the Gene Expression Omnibus (GEO) database under accession number GSE147520.^[^ ^10^ ^]^ Transcriptomic data and clinical information from The Cancer Genome Atlas (TCGA) THYM cohort were downloaded from the UCSC Xena data portal (https://xenabrowser.net). The public bulk RNA‐seq datasets (GSE29695) was also included from GEO.

### Clinical Sample Collection and Single‐Cell Preparation

Seven patients who were diagnosed with pathological TETs were enrolled in this study (NCT03197389). None of the patients were treated with chemotherapy, radiotherapy, or any other anti‐tumor treatment prior to tumor resection. The primary goal of the study was to evaluate the molecular characteristics of morphologically distinct malignant cells from different TETs subtypes.

Surgically extracted tumor and adjacent tissues were collected and divided into multiple samples, and preserved using MACS tissue storage solution (Miltenyi Biotec, #130‐100‐008) or 4% paraformaldehyde (Sigma‐Aldrich). Twenty‐five TET tissue samples from the Jiangxi Cancer Hospital (Jiangxi, China) were used to create a tissue microarray. The pathological diagnosis was applied by two pathologists (X and Y). The study was approved by the research and ethical committee of the Zhejiang Cancer Hospital (no. IRB2023758), the Jiangxi Cancer Hospital (no. 2021ky014). Informed consent was obtained from all participants and was in compliance with the Declaration of Helsinki (2013 version).

To obtain a single‐cell suspension, fresh thymic tissue was mechanically and enzymatically dissociated. Tissues were dissected into ≈4 mm cubes and collected in Roswell Park Memorial Institute (RPMI) 1640 Medium (Thermo Fisher, #11 875 093). The tissues were then digested with the gentleMACS Tumor Dissociation Kit (Miltenyi Biotec, #130‐095‐0929) and gentleMACS Dissociator (Miltenyi Biotec, #130‐093‐235) for 60 min at 37 °C. The dissociated cells were passed through a 70 µm cell strainer (BD Falcon, #352 350) and centrifuged. The cell pellets were washed and resuspended in phosphate‐buffered saline (PBS) containing 0.2% fetal bovine serum (FBS). The cells were then counted and diluted to ≈1000 live cells mL^−1^.

### Single‐Cell RNA‐Seq and Single‐Cell TCR‐Seq Library Preparation and Sequencing

Single‐cell RNA‐seq libraries were generated using a Chromium Next GEM Single Cell 5′ Kit v2 (10x Genomics, #1 000 265) according to the manufacturer's protocol. In brief, Gel bead in EMulsion (GEM) was generated by mixing a single‐cell suspension with barcoded single‐cell 5′ Gel Beads and partitioning oil on a microfluidic chip using a 10x Chromium Controller. The GEM was reverse transcribed and barcoded, and then pooled together for cDNA amplification. For single‐cell RNA‐seq libraries, amplified cDNA was fragmented, end‐repaired, amplified, and indexed following the 10x Genomics 5ʹ gene expressing protocol. For single‐cell TCR‐seq, TCR V(D)J‐targeted enrichment was performed using a Chromium Single Cell Human TCR Amplification Kit (10x Genomics, #1 000 252). Amplified TCR transcripts were then fragmented, end‐repaired, amplified, and indexed. All single‐cell 5′ gene expression libraries and single‐cell TCR‐seq were sequenced using an Illumina Novaseq 6000 platform in 150 bp paired‐end mode.

### Thymic Epithelial Tumor Tissue Processing and Organoid Culture

Tumor‐isolated single‐cells were incubated in Advanced DMEM/F12 medium (Gibco) with 1x Glutamax, 10 mM HEPES, 2 mg mL^−1^ collagenase (Worthington), 1x P/S and 1x Primocin (Invivogen) at 37 °C for 1–2 h with intermittent rotation. The digested suspensions were repeatedly triturated by pipetting and passing them through 70 µm cell strainers (BD Falcon). The strained cells were centrifuged at 400 rcf for 5 min, the pellet was resuspended in 10 mL Advanced DMEM/F12 medium, and the sample was centrifuged again at 400 rcf. The washed pellet was resuspended in 100 µL Th tumor culture medium (Advanced DMEM/F12culture medium supplemented with 50 ng mL^−1^ human EGF [Peprotech], 20 ng mL^−1^ bFGF [Peprotech], 20 ng mL^−1^ Fgf10 [Sinobiological], 50 ng mL^−1^ Wnt3a [Biogenous], 200 ng mL^−1^ R‐spondin [Sinobiological], 100 ng mL^−1^ Noggin [Sinobiological], 50 ng mL^−1^ Afamin [Sinobiological], 10 ng mL^−1^ Igf‐1 [Peprotech], 10 ng mL^−1^ IL‐2 [Peprotech], 500 ng mL^−1^ A‐8301 [Sellecks], B27 [Gibco], 10 µM Y27632 [Selleck], 1x P/S, and 1x Primocin). Half of the pellet was cryopreserved for co‐culture. The remaining pellet was resuspended in cold Matrigel (Corning) at a 1:2 ratio and 20 uL drops of the Matrigel‐cell suspension were allowed to solidify on prewarmed 48‐well suspension culture plates at 37 °C for 30 min. Upon completed gelation, 250 ul Th tumor organoid medium was added to each well and the plates were transferred to tissue culture incubators. The medium was changed every 3 days, and the organoids were passaged every 2–4 weeks. For passaging, organoids were digested in 0.5 mL TrypLE and incubated for 2–5 min at 37 °C. The pellets were washed with PBS and centrifuged at 400 rcf for 5 min. The pellets were then resuspended in Matrigel (1:2) and reseeded at 1:1–1:4 ratios to allow the formation of new organoids.

To perform co‐culture of organoids with macrophages, we selected organoids from tumors highly infiltrated with macrophages based on single‐cell RNA sequencing. A cryopreserved macrophage cell mixture was thawed and mixed with dissociated tumor organoids from the same original tumors at a 20:1 macrophage:organoid cell ratio. Co‐cultures were performed, and the medium was refreshed every three days. Organoid size was measured under a microscope.

### Immunohistochemistry and Immunofluorescence Staining and Imaging

Tumor samples were fixed in 4% paraformaldehyde, washed with PBS, embedded in paraffin, and sectioned onto microscope slides. Prior to the immune hybridization reaction, the slides were placed in an oven for 40 min at 65 °C and then deparaffinized and rehydrated through xylene and graded alcohol solutions. Antigen retrieval was performed by heating the slides to 125 °C in 10 mM sodium citrate (pH = 6) for 8 min, and the samples were blocked in 3% H_2_O_2_ and 3% bovine serum albumin (Sigma‐Aldrich, # 9048‐46‐8) with 0.1% Triton X‐100 (Sigma‐Aldrich, # 9036‐19‐5) at room temperature. The tissue was incubated with primary antibodies overnight at 4 °C (the antibody details are provided in Table S6, Supporting Information). For immunohistochemistry, staining with a biotinylated secondary antibody (Abcam, # ab64257) was performed for 1 h at room temperature, and the samples were counterstained with hematoxylin. For immunofluorescence, multiplexed fluorescence was performed with the Tyramide Signal Amplification method using Opal fluorophore packs (Akoya Biosciences, Opal 520 # FP1487001KT, Opal 570 # FP1488001KT, Opal 620 # FP1495001KT, Opal 690 # FP1497001KT) according to the instructions provided by the manufacturer. Images were acquired on a Pannoramic SCAN digital scanner (3DHISTECH, Hungary) or Olympus VS200 Slide Scanner (Olympus, Japan). The positive cells were auto‐calculated by HALO image analysis software (Indica Lab). All the fluorescence images with high resolution were presented in the Supporting Information.

### Single‐Cell RNA Sequencing Data Analysis

A Cell Ranger Single‐Cell toolkit was applied to align reads for each sample based on the human reference genome GRCh38 (https://cf.10xgenomics.com/supp/cell‐exp/refdata‐gex‐GRCh38‐2020‐A.tar.gz). Single‐cell downstream analysis was based on the Seurat R package.^[^ ^56^ ^]^ Further quality control was applied to cells based on the following thresholds: 1) the number of expressed genes was greater than 150 and less than 6000; 2) the cells contained less than 10% mitochondrial RNA. The DoubletFinder^[^ ^57^ ^]^ R package was applied to remove potential doublets. The filtered gene expression matrix for each sample was normalized and scaled by the “NormalizeData” and “ScaleData” functions in Seurat. The Harmony R package was used to adjust batch effects between different patients and integrate the gene expression matrices of all samples.^[^ ^58^ ^]^ Finally, 36 601 genes were identified and assessed 159 607 cells from 20 samples. The principal component analysis (PCA) was performed on the corrected expression matrix using highly variable genes (HVGs) identified by the “FindVariableGenes” function. Next, the “RunPCA” function was used to perform the PCA and the “FindNeighbors” function was used to construct a K‐nearest‐neighbor graph. The most representative principal components were used to determine different cell types with the “FindCluster” function. The cell types were annotated and 8 clusters were identified based on expression of the following marker genes: CD79A and MS4A1 for B cells, VWF and PECAM1 for endothelial cells, KRT8 and KRT17 for epithelial cells, COL1A1 and COL6A1 for fibroblasts, MS4A2 and CPA3 for mast cells, CD14 and FCGR3A for myeloid cells, TNFRSF17 and IGHG1 for plasma B cells, and CD3D and CD3E for T cells. All epithelial cells and myeloid cells were re‐clustered unsupervised to obtain functional subclusters. The unsupervised clustering analysis was conducted on all T cells and used the expression levels of CD34, CD38, CD3E, CD3D, CD3G, CD4, CD8A, CD8B, TRDC, and TRGC2 to establish a more detailed T cell evolution process. The unsupervised clustering was performed on mature CD4^+^ T cells or CD8^+^ T cells and identified subpopulation functions based on highly expressed genes.

### Differential Expression Analysis

To identify differentially expressed genes for each cell subtype, the “wilcoxauc” functions from the presto package were used with default parameters. The expression differences with *P* < 0.05 and log2(fold change, FC) > 0.3 were considered differentially expressed genes.

### Epithelial Cell Characterization

Fourteen epithelial cell subtypes were identified and annotated based on the expression of marker genes. These cells included cortical thymic epithelial cells (mTEC, PRSS16, and PSMB11),^[^ ^59^ ^]^ intermediate thymic epithelial cells (mcTEC, CCL19, and KRT14),^[^ ^13^ ^]^ medullary thymic epithelial cells (mTEC, CLDN4, and CLDN3),^[^ ^60^ ^]^ enteroendocrine cells (CHGB and CHGA),^[^ ^8^ ^]^ ionocyte cells (FOXI1 and ASCL3),^[^ ^61^ ^]^ and myoid cells (MYOG and MYL5).^[^ ^62^ ^]^ The “irGSEA” R package (https://github.com/chuiqin/irGSEA/) was used to score individual cells, using a multi‐genomic enrichment approach, to generate a multi‐genomic enrichment scoring matrix with the Hallmark or Kyoto Encyclopedia of Genes and Genomes (KEGG) pathways being derived. Based on previous research on thymic epithelial cell development, the cTEC, mTEC, early progenitor, and postnatal signatures were identified.^[^ ^60^ ^]^

### Copy Number Variation Calling

The inferCNV R package (inferCNV of the Trinity CTAT Project, provided at https://github.com/broadinstitute/inferCNV) was used to infer the large‐scale chromosomal copy number variations of each cell. Other parameters were set to default.

### Tissue Preference of Each Cell Subtype

The ratio of observed to expected cell numbers was calculated in each cell subtype (Ro/e) to quantify the preference of each cell subtype across tissues, as previously described.^[^ ^63^ ^]^ In brief, the expected cell numbers of each cell subtype in each tissue were obtained from the Chi‐square test, and Ro/e > 1 for a cell subtype in a tissue indicated preference of this cell subtype in this tissue.

### Genomic Instability Estimation

To estimate the genomic instability of each malignant cell, we used the genomicInstability R package, which uses the aREA algorithm to quantify the enrichment of sets of contiguous genes (loci‐blocks), on the gene expression profiles to estimate the Genomic Instability Score (GIS) for each analyzed cell.

### Cell Developmental Trajectory Analysis

RNA velocity analysis was conducted using velocyto^[^ ^64^ ^]^ and scVelo.^[^ ^65^ ^]^ The 10 × velocyto pipeline was used to count spliced and non‐spliced reads for each sample from cellranger‐generated BAM files. To predict the root and terminal states of the underlying Markov process, the respective scVelo function was applied. The python package, PAGA,^[^ ^66^ ^]^ was also used to verify the pseudotime between each epithelial cell subtype. The single‐cell trajectory analysis of myofibroblast cell subtypes was performed using Monocle2^[^ ^67^ ^]^ with DDR‐Tree and default parameters.

### Identification of Master Transcription Factors

Master transcription factors that regulate differentially expressed genes in epithelial cells were analyzed using the plugin iRegulon^[^ ^68^ ^]^ in Cytoscape network.^[^ ^69^ ^]^ iRegulon used > 9000 known position weight matrices from various sources and different species. Candidate binding TFs were identified using a “motif2TF” procedure. Predicted master transcription factors were ranked according to the normalized enrichment score.

### Cell‐Cell Interaction Analysis

CellChat^[^ ^70^ ^]^ was used with default parameters to identify significant ligand‐receptor pairs within primary TET samples. All categories of ligand‐receptor interactions were used in the database for the analysis. Finally, the “netVisual_bubble” function was applied to visualize communication probabilities by ligand‐receptor pairs in different directions.

### Single‐Cell Flux Estimation and Cell Metabolite Prediction

Scfea^[^ ^71^ ^]^ tools were used, which utilizes a graph neural network model, to estimate cell‐wise metabolic flux from the scRNA‐seq data. The “module gene m168” was chosen as the moduleGene file, “cmMat c70 m168” as the stoichiometry file, and the parameter “sc imputation = True”.

### Cell‐Cell Metabolic Communication

MEBOCOST,^[^ ^72^ ^]^ a Python‐based computational tool was used to infer metabolite‐mediated cell‐cell communication events. The cutoff was set to 0.25 and other parameters were set to default.

### Whole Exome Sequencing (WES) Data Processing

The WES data was aligned by BWA mem software with the genome reference hg38, and then duplicates were removed using sambamba markdup, realign, and recal with GATK RealignerTargetCreator, IndelRealigner, BaseRecalibrator tools, call somatical variation with Mutect2, and variation filter with GATK FilterMutectCalls and SelectVariants tools. Finally, we annotates these variation using annovar software.

### Single‐Cell Mutation Analysis

The downstream BAM file was used from cellranger, a single‐cell quantification program, and the vcf file obtained from the mutect2 program in GATK software to identify somatic mutations in the WES analysis. The cellsnp‐lite software^[^ ^73^ ^]^ was used to determine the mutations in each single cell that have the same genomic positions as identified in the WES analysis. The parameter settings used were ‐minMAF 0.1 and –minCOUNT 20.

### TCR Analysis

Full‐length TCR V(D)J segments were enriched from amplified cDNA from 5′ libraries via PCR amplification using a Chromium Single‐Cell V(D)J Enrichment kit according to the manufacturer's protocol (10x Genomics). The TCR sequences for each single T cell were assembled using the Cell Ranger vdj pipeline (v3.1.0), leading to the identification of the CDR3 sequence and the rearranged TCR gene. Analysis was performed using Loupe V(D)J Browser v.2.0.1 (10x Genomics). In brief, a TCR diversity metric, containing clonotype frequency and barcode information, was obtained. T cells containing TCRs with two α and/or two β chains were identified. Multiple T cells containing the same TCR form exclude the possibility of doublets. Four TCR forms, 1α1β, 1α2β, 2α1β, and 2α2β, were thus considered in the analysis. The same form of TCR was considered as one class, and was assigned a unique TCR name. We visualized the TCR count and TCR frequency statistics in T1, T2, and T3 samples using the scRepertoire package.^[^ ^74^ ^]^

### Estimation of the Corresponding Cell Fractions in TCGA‐THYM RNA‐Seq Data

A computational R package Microenvironment Cell Populations‐counter (MCP‐counter)^[^ ^75^ ^]^ was used to estimate the cell fractions of epithelial clusters, CD4^+^ T cell clusters, and CD8^+^ T cell clusters from the TCGA‐THYM RNA‐seq data. The LGALS9‐SLC1A5 or CCL20‐CCR6 interaction network scores were the product of multiplying the expression levels of LGALS9 with SLC1A5, or CCL20 with CCR6, respectively.

### Cell Subtype Similarity Analysis

The following steps were used to evaluate the similarity of cell subtypes:^[^ ^1^ ^]^ Identify 1000 most variable genes across different cell subtypes,^[^ ^2^ ^]^ calculate the mean value of the 1000 most variable genes in each cell subtype, and^[^ ^3^ ^]^ cluster the hierarchy, using the distance defined by (1‐Pearson correlation coefficient)/2.

### Statistical Analysis

We chose to perform a Spearman correlation coefficient test to conduct a correlation analysis of the expression levels of two genes in the TCGA or GEO data. The Kaplan‐Meier method was used to estimate OS or PFS, with the log‐rank test used to compare the Kaplan‐Meier curves. A two‐sided P value of less than 0.05 was considered significant. All sample sizes were large enough to ensure proper statistical analysis. Statistical analyses were performed using GraphPad Prism (GraphPad Software, Inc.). P values < 0.05 were considered statistically significant. All analyses were one‐tailed t‐tests or Wilcoxon tests (paired or unpaired depending on the experiments). The number of replicates (n), number of independent experiments performed, and P values for each experiment are reported in the corresponding figure legends.

## Conflict of Interest

The authors declare no conflict of interest.

## Author Contributions

X.L., C.W., Y.H., and Q.L. contributed equally to this work. A.Z., W.‐M.M., and Z.‐X.Z. designed the study. A.Z. and W.‐M.M. procured financial support. C.‐C.W., Y.‐Y.H., C.Y., J.‐H.Y., Y.‐Z.G., Q.‐L.L., and W.‐H.S. collected and prepared the samples. X.L., Y.‐Y.H., C.Y., J.‐H.Y., L.‐H.D., G.‐Y.H., W.‐H.S., and M.J. collected the data and performed the methodology. X.L., Y.‐Y.H., J.‐H.Y., L.‐H.D., G.‐Y.H., and M.J. performed the statistical analyses. X.L., Y.‐Y.H., J.‐H.Y., L.‐H.D., G.‐Y.H., M.J., A.Z., and Z.‐X.Z. interpreted the data. X.L. and A.Z. wrote the original draft. All other authors contributed to the data discussion and interpretation processes. All authors revised and reviewed the manuscript and approved the final version.

## Supporting information



Supporting Information

## Data Availability

The data that support the findings of this study are available from the corresponding author upon reasonable request.

## References

[1] J. E. Visvader , Nature 2011, 469, 314. 21248838 10.1038/nature09781

[2] D. Suster , S. Suster , Adv Anat Pathol 2023, 31, 22. 37702296 10.1097/PAP.0000000000000412

[3] K. Togashi , Y. Hosaka , M. Saito , K. Sato , H. Usuda , I. Emura , Kyobu Geka 2007, 60, 187. 17352134

[4] V. Hoffacker , A. Schultz , J. J. Tiesinga , R. Gold , B. Schalke , W. Nix , R. Kiefer , H. K. Mu?ller‐Hermelink , A. Marx , Blood 2000, 96, 3872. 11090072

[5] Q. Jia , H. Chu , Z. Jin , H. Long , B. Zhu , Signal Transduct Target Ther 2022, 7, 145. 35504878 10.1038/s41392-022-00990-4 PMC9065032

[6] Y. Xu , G. H. Su , D. Ma , Y. Xiao , Z. M. Shao , Y. Z. Jiang , Signal Transduct Target Ther 2021, 6, 312. 34417437 10.1038/s41392-021-00729-7 PMC8377461

[7] J. E. Park , R. A. Botting , C. Dominguez Conde , D. M. Popescu , M. Lavaert , D. J. Kunz , I. Goh , E. Stephenson , R. Ragazzini , E. Tuck , A. Wilbrey‐Clark , K. Roberts , V. R. Kedlian , J. R. Ferdinand , X. He , S. Webb , D. Maunder , N. Vandamme , K. T. Mahbubani , K. Polanski , L. Mamanova , L. Bolt , D. Crossland , F. de Rita , A. Fuller , A. Filby , G. Reynolds , D. Dixon , K. Saeb‐Parsy , S. Lisgo , et al., Science 2020, 367, aay3224. 10.1126/science.aay3224 PMC7611066 32079746

[8] C. Bornstein , S. Nevo , A. Giladi , N. Kadouri , M. Pouzolles , F. Gerbe , E. David , A. Machado , A. Chuprin , B. Tóth , O. Goldberg , S. Itzkovitz , N. Taylor , P. Jay , V. S. Zimmermann , J. Abramson , I. Amit , Nature 2018, 559, 622. 30022162 10.1038/s41586-018-0346-1

[9] R. Ragazzini , S. Boeing , L. Zanieri , M. Green , G. D'Agostino , K. Bartolovic , A. Agua‐Doce , M. Greco , S. A. Watson , A. Batsivari , L. Ariza‐McNaughton , A. Gjinovci , D. Scoville , A. Nam , A. C. Hayday , D. Bonnet , P. Bonfanti , Dev. Cell 2023, 58, 2428. 37652013 10.1016/j.devcel.2023.08.017 PMC10957394

[10] J. L. Bautista , N. T. Cramer , C. N. Miller , J. Chavez , D. I. Berrios , L. E. Byrnes , J. Germino , V. Ntranos , J. B. Sneddon , T. D. Burt , J. M. Gardner , C. J. Ye , M. S. Anderson , A. V. Parent , Nat. Commun. 2021, 12, 1096. 33597545 10.1038/s41467-021-21346-6 PMC7889611

[11] A. Marx , D. Belharazem , D.e‐H. Lee , Z. V. Popovic , C. Reißfelder , B. Schalke , S. Schölch , P. Ströbel , C.‐A. Weis , Y. Yamada , Virchows Arch 2021, 478, 101. 33674910 10.1007/s00428-021-03068-8 PMC7966134

[12] A. F. Artal‐Cortes , J. Verdun‐Aguilar , D. Marquez‐Medina , J Thorac Oncol 2023, 18, 136. 36682839 10.1016/j.jtho.2022.11.029

[13] A. Nusser , Sagar , J. B. Swann , B. Krauth , D. Diekhoff , L. Calderon , C. Happe , D. Grun , T. Boehm , Nature 2022, 606, 165. 35614226 10.1038/s41586-022-04752-8 PMC9159946

[14] M. Radovich , C. R. Pickering , I. Felau , G. Ha , H. Zhang , H. Jo , K. A. Hoadley , P. Anur , J. Zhang , M. McLellan , R. Bowlby , T. Matthew , L. Danilova , A. M. Hegde , J. Kim , M. D. M. Leiserson , G. Sethi , C. Lu , M. Ryan , X. Su , A. D. Cherniack , G. Robertson , R. Akbani , P. Spellman , J. N. Weinstein , D. N. Hayes , B. Raphael , T. Lichtenberg , K. Leraas , J. C. Zenklusen , et al., Cancer Cell 2018, 33, 244. 29438696 10.1016/j.ccell.2018.01.003 PMC5994906

[15] D. I. Suster , A. Craig Mackinnon , M. DiStasio , M. K. Basu , G. Pihan , S. Suster , Mod Pathol 2022, 35, 244875. 10.1038/s41379-022-01013-x 35145198

[16] M. Tsang , K. Quesnel , K. Vincent , J. Hutchenreuther , L. M. Postovit , A. Leask , Am J Pathol 2020, 190, 206. 31610176 10.1016/j.ajpath.2019.09.006

[17] C.‐A. Weis , X. Yao , Y. Deng , F. C. Detterbeck , M. Marino , A. G. Nicholson , J. Huang , P. Ströbel , A. Antonicelli , A. Marx , J Thorac Oncol 2015, 10, 367. 25616178 10.1097/JTO.0000000000000393 PMC4318643

[18] K. Wells , A. Lamrca , G. Papaxoinis , A. Wallace , A. M. Quinn , Y. Summers , D. Nonaka , J Clin Pathol 2023, 76, 463. 35039450 10.1136/jclinpath-2021-207837

[19] M. The?veniau‐Ruissy , M. Dandonneau , K. Mesbah , O. Ghez , M.‐G.`V.e Mattei , L. Miquerol , R. G. Kelly , Circ. Res. 2008, 103, 142. 18583714 10.1161/CIRCRESAHA.108.172189

[20] E. C. Zook , P. A. Krishack , S. Zhang , N. J. Zeleznik‐Le , A. B. Firulli , P. L. Witte , P. T. Le , Blood 2011, 118, 5723. 21908422 10.1182/blood-2011-03-342097 PMC3228493

[21] M. A. McGargill , D. Mayerova , H. E. Stefanski , B. Koehn , E. A. Parke , S. C. Jameson , A. Panoskaltsis‐Mortari , K. A. Hogquist , J. Immunol. 2002, 169, 2141. 12165543 10.4049/jimmunol.169.4.2141

[22] P. M. Maciocia , P. A. Wawrzyniecka , B. Philip , I. Ricciardelli , A. U. Akarca , S. C. Onuoha , M. Legut , D. K. Cole , A. K. Sewell , G. Gritti , J. Somja , M. A. Piris , K. S. Peggs , D. C. Linch , T. Marafioti , M. A. Pule , Nat. Med. 2017, 23, 1416. 29131157 10.1038/nm.4444

[23] A. R. Glabinski , B. Bielecki , S. O'Bryant , K. Selmaj , R. M. Ransohoff , J Autoimmun 2002, 19, 175. 12473238 10.1006/jaut.2002.0613

[24] H. Kurobe , C. Liu , T. Ueno , F. Saito , I. Ohigashi , N. Seach , R. Arakaki , Y. Hayashi , T. Kitagawa , M. Lipp , R. L. Boyd , Y. Takahama , Immunity 2006, 24, 165. 16473829 10.1016/j.immuni.2005.12.011

[25] G. Giaccone , C. Kim , J Thorac Oncol 2021, 16, 483. 33248322 10.1016/j.jtho.2020.11.003

[26] T. Zander , S. Aebi , A. C. Rast , A. Zander , R. Winterhalder , C. Brand , J. Diebold , O. Gautschi , J Thorac Oncol 2016, 11, e147. 27498287 10.1016/j.jtho.2016.07.018

[27] Y.‐Q. Ao , J. Gao , S. Wang , J.‐H. Jiang , J. Deng , H.‐K. Wang , B. Xu , J.‐Y. Ding , Mol Cancer 2023, 22, 70. 37055838 10.1186/s12943-023-01772-4 PMC10099901

[28] R. N. Germain , Nat. Rev. Immunol. 2002, 2, 309. 12033737 10.1038/nri798

[29] B. J. Laidlaw , J. E. Craft , S. M. Kaech , Nat. Rev. Immunol. 2016, 16, 102. 26781939 10.1038/nri.2015.10 PMC4860014

[30] V. Chen , S. Umemura , Y. Han , R. Raman , R. Tucker , J. Chahine , I.n‐K. Kim , C. Schatz , S. Zitzmann‐Kolbe , A. Sommer , M. Onda , T. Lee , Y. He , G. Giaccone , Br. J. Cancer 2022, 126, 754. 34876673 10.1038/s41416-021-01658-6 PMC8888701

[31] I. Petrini , P. A. Zucali , H. S. Lee , M. A. Pineda , P. S. Meltzer , B. Walter‐Rodriguez , M. Roncalli , A. Santoro , Y. Wang , G. Giaccone , J Thorac Oncol 2010, 5, 1447. 20651610 10.1097/JTO.0b013e3181e96e30 PMC7328988

[32] C. C. Pan , P. C. Chen , H. Chiang , J Pathol 2004, 202, 375. 14991904 10.1002/path.1514

[33] M. L. Broz , M. Binnewies , B. Boldajipour , A. E. Nelson , J. L. Pollack , D. J. Erle , A. Barczak , M. D. Rosenblum , A. Daud , D. L. Barber , S. Amigorena , L. J. van't Veer , A. I. Sperling , D. M. Wolf , M. F. Krummel , Cancer Cell 2014, 26, 638. 25446897 10.1016/j.ccell.2014.09.007 PMC4254577

[34] E. Sikora , A. Bielak‐Zmijewska , G. Mosieniak , Ageing Res. Rev. 2021, 71, 101458. 34500043 10.1016/j.arr.2021.101458

[35] L. Ma , S. Heinrich , L. Wang , F. L. Keggenhoff , S. Khatib , M. Forgues , M. Kelly , S. M. Hewitt , A. Saif , J. M. Hernandez , D. Mabry , R. Kloeckner , T. F. Greten , J. Chaisaingmongkol , M. Ruchirawat , J. U. Marquardt , X. W. Wang , Nat. Commun. 2022, 13, 7533. 36476645 10.1038/s41467-022-35291-5 PMC9729309

[36] H. C. Yoo , S. J. Park , M. Nam , J. Kang , K. Kim , J. H. Yeo , J.‐K.i Kim , Y. Heo , H. S. Lee , M. Y. Lee , C. W. Lee , J. S. Kang , Y.‐H. Kim , J. Lee , J. Choi , G.‐S. Hwang , S. Bang , J. M. Han , Cell Metab. 2020, 31, 267. 31866442 10.1016/j.cmet.2019.11.020

[37] M. Shang , F. Cappellesso , R. Amorim , J. Serneels , F. Virga , G. Eelen , S. Carobbio , M. Y. Rincon , P. Maechler , K. De Bock , P.‐C. Ho , M. Sandri , B. Ghesquière , P. Carmeliet , M. Di Matteo , E. Berardi , M. Mazzone , Nature 2020, 587, 626. 33116312 10.1038/s41586-020-2857-9 PMC7116844

[38] L. W. Plasschaert , R. Zilionis , R. Choo‐Wing , V. Savova , J. Knehr , G. Roma , A. M. Klein , A. B. Jaffe , Nature 2018, 560, 377. 30069046 10.1038/s41586-018-0394-6 PMC6108322

[39] C. P. Casellas , C. Pleguezuelos‐Manzano , M. B. Rookmaaker , M. C. Verhaar , H. Clevers , Sci. Rep. 2023, 13, 3516. 36864051 10.1038/s41598-023-30603-1 PMC9981729

[40] J. A. Bonanno , R. Shyam , M. Choi , D. G. Ogando , Cells 2022, 11, 197. 35053313 10.3390/cells11020197 PMC8773465

[41] J. Qi , H. Sun , Y. Zhang , Z. Wang , Z. Xun , Z. Li , X. Ding , R. Bao , L. Hong , W. Jia , F. Fang , H. Liu , L. Chen , J. Zhong , D. Zou , L. Liu , L. Han , F. Ginhoux , Y. Liu , Y. Ye , B. Su , Nat. Commun. 2022, 13, 1971742. 10.1038/s41467-022-29366-6 PMC8976074 35365629

[42] X. Xu , M. C. Farach‐Carson , X. Jia , Biotechnol. Adv. 2014, 32, 1256. 25116894 10.1016/j.biotechadv.2014.07.009 PMC4171250

[43] D. Rodenhizer , T. Dean , B. Xu , D. Cojocari , A. P. McGuigan , Nat. Protoc. 2018, 13, 1917. 30190554 10.1038/s41596-018-0022-9

[44] E. Infante , A. Stannard , S. J. Board , P. Rico‐Lastres , E. Rostkova , A. E. M. Beedle , A. Lezamiz , Y. J. Wang , S. Gulaidi Breen , F. Panagaki , V. Sundar Rajan , C. Shanahan , P. Roca‐Cusachs , S. Garcia‐Manyes , Nat. Phys. 2019, 15, 973. 37484710 10.1038/s41567-019-0551-3 PMC7614795

[45] S. L. Dunn , J. Soul , S. Anand , J. M. Schwartz , R. P. Boot‐Handford , T. E. Hardingham , Osteoarthritis Cartilage 2016, 24, 1431. 26973327 10.1016/j.joca.2016.03.007 PMC4989048

[46] C. Cao , Y. Cai , Y. Li , T. Li , J. Zhang , Z. Hu , J. Zhang , Poult Sci 2023, 102, 103122. 37832186 10.1016/j.psj.2023.103122 PMC10568565

[47] Z. Xin , M. Lin , Z. Hao , D.i Chen , Y. Chen , X. Chen , X. Xu , J. Li , D. Wu , Y. Chai , P. Wu , Nat. Commun. 2022, 13, 5463. 36115836 10.1038/s41467-022-33170-7 PMC9482639

[48] M. Capone , R. K. Lees , D. Finke , B. Ernst , J. P. Meerwijk , H. R. MacDonald , Eur. J. Immunol. 2003, 33, 1471. 12778464 10.1002/eji.200323754

[49] D. Suster , A. C. Mackinnon , G. Pihan , R. Everts , S. Suster , Am J Surg Pathol 2022, 46, 603. 35034040 10.1097/PAS.0000000000001855

[50] K. D. James , D. F. Legler , V. Purvanov , I. Ohigashi , Y. Takahama , S. M. Parnell , A. J. White , W. E. Jenkinson , G. Anderson , Blood Adv 2021, 5, 99. 33570638 10.1182/bloodadvances.2020003192 PMC7805325

[51] T. Tokatlian , G. E. Asuelime , J. Y. Mock , B. DiAndreth , S. Sharma , D. Toledo Warshaviak , M. E Daris , K. Bolanos , B. L Luna , M. S Naradikian , K. Deshmukh , A. E Hamburger , A. Kamb , J Immunother Cancer 2022, 10, e003826. 35091455 10.1136/jitc-2021-003826 PMC8804709

[52] R. Yang , L. Sun , C.‐F. Li , Y.u‐H. Wang , W. Xia , B. Liu , Y.u‐Y.i Chu , L. Bover , L. Vien , M.‐C. Hung , J. Biol. Chem. 2022, 298, e003826101821. 10.1016/j.jbc.2022.101821 PMC9006662 35283189

[53] L. Lei , S. Traore , G. S. Romano Ibarra , P. H. Karp , T. Rehman , D. K. Meyerholz , J. Zabner , D. A. Stoltz , P. L. Sinn , M. J. Welsh , P. B. McCray Jr. , I. M. Thornell , J. Clin. Invest. 2023, 133, JCI171268. 10.1172/JCI171268 PMC10575720 37581935

[54] Y. Lu , P. Zuo , H. Chen , H. Shan , W. Wang , Z. Dai , H.e Xu , Y. Chen , L. Liang , D. Ding , Y. Jin , Y. Yin , Nat. Commun. 2023, 14, 6157. 37788993 10.1038/s41467-023-41924-0 PMC10547724

[55] D. G. Ogando , M. Choi , R. Shyam , S. Li , J. A. Bonanno , Redox Biol. 2019, 26, 101260. 31254733 10.1016/j.redox.2019.101260 PMC6604051

[56] A. Butler , P. Hoffman , P. Smibert , E. Papalexi , R. Satija , Nat. Biotechnol. 2018, 36, 411. 29608179 10.1038/nbt.4096 PMC6700744

[57] C. S. McGinnis , L. M. Murrow , Z. J. Gartner , Cell Syst 2019, 8, 329 . 30954475 10.1016/j.cels.2019.03.003 PMC6853612

[58] I. Korsunsky , N. Millard , J. Fan , K. Slowikowski , F. Zhang , K. Wei , Y. Baglaenko , M. Brenner , P. o‐R. u. Loh , S. Raychaudhuri , Nat. Methods 2019, 16, 1289. 31740819 10.1038/s41592-019-0619-0 PMC6884693

[59] T. Boehm , Eur. J. Immunol. 2009, 39, 944. 19350578 10.1002/eji.200939315

[60] Y. Kawai , Y. Hamazaki , H. Fujita , A. Fujita , T. Sato , M. Furuse , T. Fujimoto , A. M. Jetten , Y. Agata , N. Minato , Proc Natl Acad Sci U S A 2011, 108, 4075. 21325057 10.1073/pnas.1014178108 PMC3054041

[61] Y. Sato , D. Kim , M. J. Turner , Y. Luo , S. S. Z. Zaidi , D. Y. Thomas , J. W. Hanrahan , Am. J. Respir. Cell Mol. Biol. 2023, 69, 281. 36952679 10.1165/rcmb.2022-0241OC

[62] B.o Hu , K. Simon‐Keller , S. Küffer , P. Ströbel , T. Braun , A. Marx , S. Porubsky , Exp Neurol 2016, 277, 76. 26708556 10.1016/j.expneurol.2015.12.010

[63] J. He , X. Xiong , H. Yang , D. Li , X. Liu , S. Li , S. Liao , S. Chen , X. Wen , K. Yu , L. Fu , X. Dong , K. Zhu , X. Xia , T. Kang , C. Bian , X. Li , H. Liu , P. Ding , X. Zhang , Z. Liu , W. Li , Z. Zuo , P. Zhou , Cell Res. 2022, 32, 530. 35165422 10.1038/s41422-022-00627-9 PMC9160085

[64] G. La Manno , R. Soldatov , A. Zeisel , E. Braun , H. Hochgerner , V. Petukhov , K. Lidschreiber , M. E. Kastriti , P. Lönnerberg , A. Furlan , J. Fan , L. E. Borm , Z. Liu , D. van Bruggen , J. Guo , X. He , R. Barker , E. Sundström , G. Castelo‐Branco , P. Cramer , I. Adameyko , S. Linnarsson , P. V. Kharchenko , Nature 2018, 560, 494. 30089906 10.1038/s41586-018-0414-6 PMC6130801

[65] V. Bergen , M. Lange , S. Peidli , F. A. Wolf , F. J. Theis , Nat. Biotechnol. 2020, 38, 1408. 32747759 10.1038/s41587-020-0591-3

[66] F. A. Wolf , F. K. Hamey , M. Plass , J. Solana , J. S. Dahlin , B. Göttgens , N. Rajewsky , L. Simon , F. J. Theis , Genome Biol. 2019, 20, 59. 30890159 10.1186/s13059-019-1663-x PMC6425583

[67] X. Qiu , Q.i Mao , Y. Tang , L.i Wang , R. Chawla , H. A. Pliner , C. Trapnell , Nat. Methods 2017, 14, 979. 28825705 10.1038/nmeth.4402 PMC5764547

[68] R.'s Janky , A. Verfaillie , H. Imrichová , B. Van de Sande , L. Standaert , V. Christiaens , G. Hulselmans , K. Herten , M. Naval Sanchez , D. Potier , D. Svetlichnyy , Z. Kalender Atak , M. Fiers , J.‐C. Marine , S. Aerts , PLoS Comput. Biol. 2014, 10, e1003731. 25058159 10.1371/journal.pcbi.1003731 PMC4109854

[69] P. Shannon , A. Markiel , O. Ozier , N. S. Baliga , J. T. Wang , D. Ramage , N. Amin , B. Schwikowski , T. Ideker , Genome Res. 2003, 13, 2498. 14597658 10.1101/gr.1239303 PMC403769

[70] S. Jin , C. F. Guerrero‐Juarez , L. Zhang , I. Chang , R. Ramos , C.‐H. Kuan , P. Myung , M. V. Plikus , Q. Nie , Nat. Commun. 2021, 12, 1088. 33597522 10.1038/s41467-021-21246-9 PMC7889871

[71] N. Alghamdi , W. Chang , P. Dang , X. Lu , C. Wan , S. Gampala , Z. Huang , J. Wang , Q. Ma , Y. Zang , M. Fishel , S. Cao , C. Zhang , Genome Res. 2021, 31, 1867. 34301623 10.1101/gr.271205.120 PMC8494226

[72] K. Cui , X. Gao , B. Wang , H. Wu , K. Arulsamy , Y. Dong , Y. Xiao , X. Jiang , M. V. Malovichko , K. Li , Q. Peng , Y. W. Lu , B.o Zhu , R. Zheng , S. Wong , D. B. Cowan , M. Linton , S. Srivastava , J. Shi , K. Chen , H. Chen , Circ. Res. 2023, 132, e22. 36444722 10.1161/CIRCRESAHA.122.321723 PMC9822875

[73] X. Huang , Y. Huang , Bioinformatics 2021, 37, 4569. 33963851 10.1093/bioinformatics/btab358

[74] N. Borcherding , N. L. Bormann , G. Kraus , F1000Res 2020, 9, 47. 32789006 10.12688/f1000research.22139.1 PMC7400693

[75] E. Becht , N. A. Giraldo , L. Lacroix , B. Buttard , N. Elarouci , F. Petitprez , J. Selves , P. Laurent‐Puig , C. Sautès‐Fridman , W. H. Fridman , A. de Reyniès , Genome Biol. 2016, 17, 218. 27765066 10.1186/s13059-016-1070-5 PMC5073889

