# Supplementary material for: Abnormal Cellular Populations Shape Thymic Epithelial Tumor Heterogeneity and Anti‐Tumor by Blocking Metabolic Interactions in Organoids
Source: Adv Sci (Weinh). 2024 Sep 11;11(42):2406653. doi: 10.1002/advs.202406653 (PMC11558144; doi:10.1002/advs.202406653)
Supplement: Supplementary file 1 — Supporting Information [file ADVS-11-2406653-s001.docx]

**Supporting Information**

**Abnormal cellular populations shape thymic epithelial tumor heterogeneity and anti-tumor by blocking metabolic interactions in organoids**

Xuefei Liu^1,2,3,^^*^, Changchun Wang^4,*^, Yueyu Huang^1,*^, Qiaoli Lv^5,*^, Chang Yu^6^, Jianghua Ying^7^, Lianhui Duan^2^, Yangzhong Guo^5^, Guanyin Huang^2^, Wenhui Shen^1^, Ming Jiang^8,9^,

Weimin Mao^10,5,†^, Zhixiang Zuo^11,†^, An Zhao^1,5,†,‡^

- **Supplementary** **Figures and Legends**
- **Supplementary Tables**
- **High-resolution H&E, IHC and mIF images for Figures**

**Supplementary** **Figures and Legends**

**
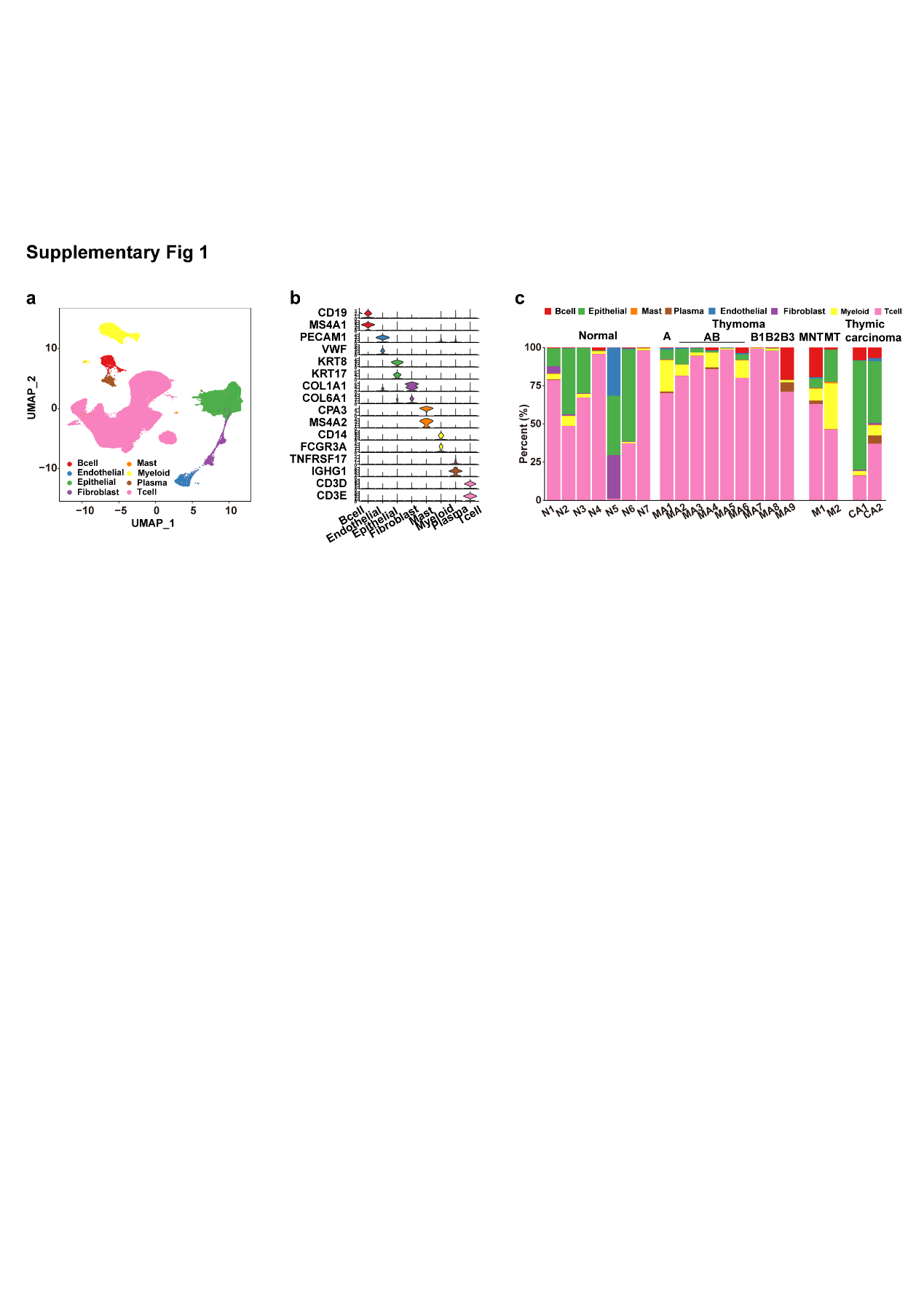
**

**Figure S1. Single-cell transcriptional profiling of thymus reveals cellular heterogeneity.**

a, UMAP of 159,607 cells analyzed by scRNA-seq across all samples. Clusters are annotated using canonical markers.

b, Violin plot showing the expression levels of the selected markers in the indicated cell subtypes.

c, Bar plot showing the relative proportions of cell subtypes across each patient identified by WHO classification.

**
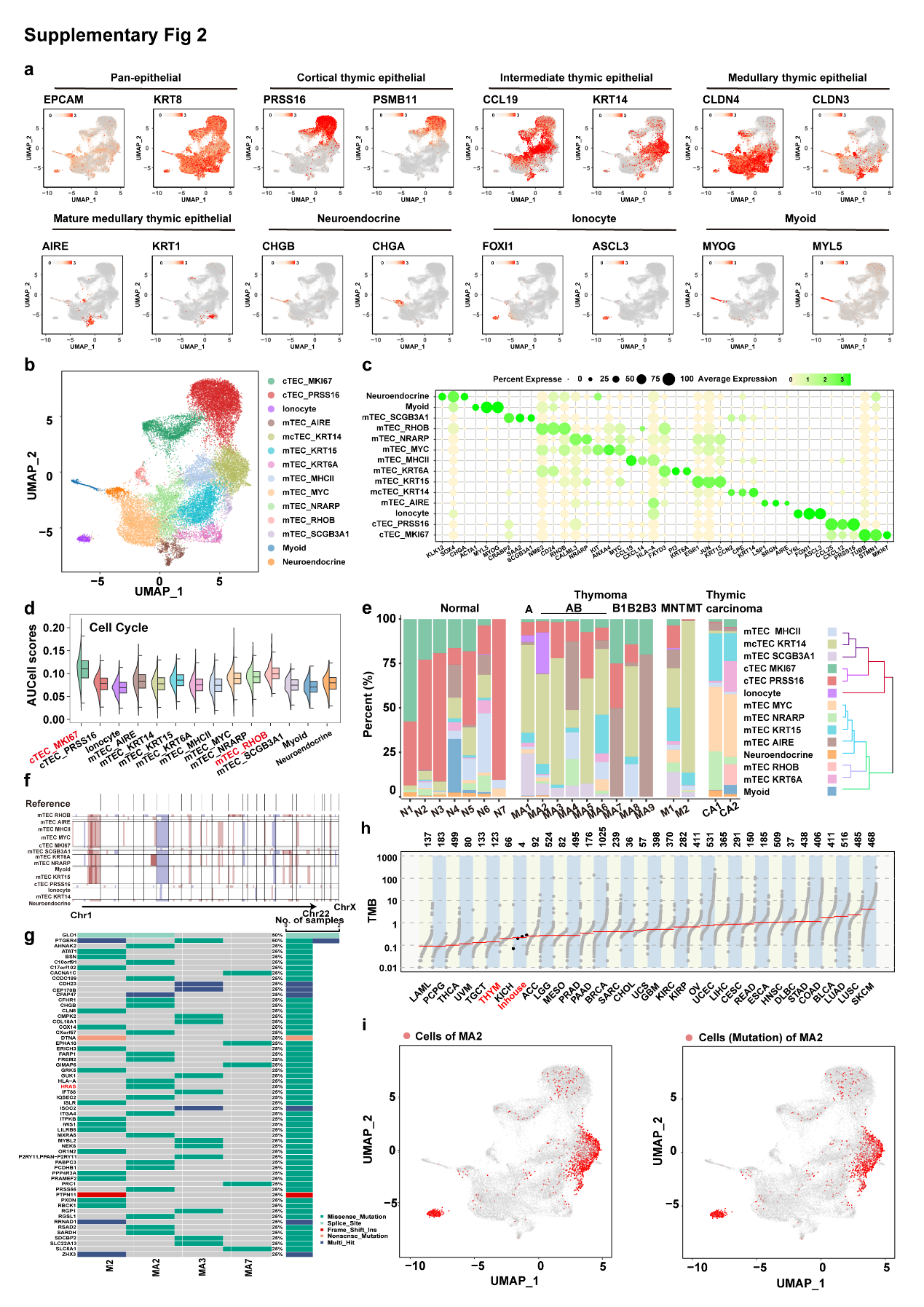
**

**Figure S2. Characterization of epithelial cells in normal and tumoral thymus**

a, UMAP plot showing the expression levels of marker genes, defined for all cell types.

b, UMAP plot showing 14 epithelial cell subpopulations.

c, The expression levels of the selected markers in different epithelial cell subtypes. Dot size indicates the fraction of expressing cells and the colors represent normalized gene expression levels.

d, Box plot showing the cell cycle scores among the 14 epithelial cell subtypes. Cell cycle scores were calculated using AUCell.

e, Bar plot showing the relative proportions of epithelial cell subtypes across each patient identified by WHO classification.

f, Heatmap showing large-scale CNVs for individual cells (rows) from tumor tissue. Non-malignant cells from normal tissue were used as references (top), with large-scale CNVs observed in malignant cells (bottom). Colors indicate the log2CNV ratio. Red: amplifications; blue: deletions.

g, Waterfall plot of the distribution of mutations found in four patients by whole-exome sequencing (WES).

h, Distribution of the mutational burden for each cohort and each tumor type of the in-house cohort and the TCGA cohort.

i, UMAP plot showing cells from MA2 patients (left panel); the cells have the same sites identified by the MA2 WES data.

**
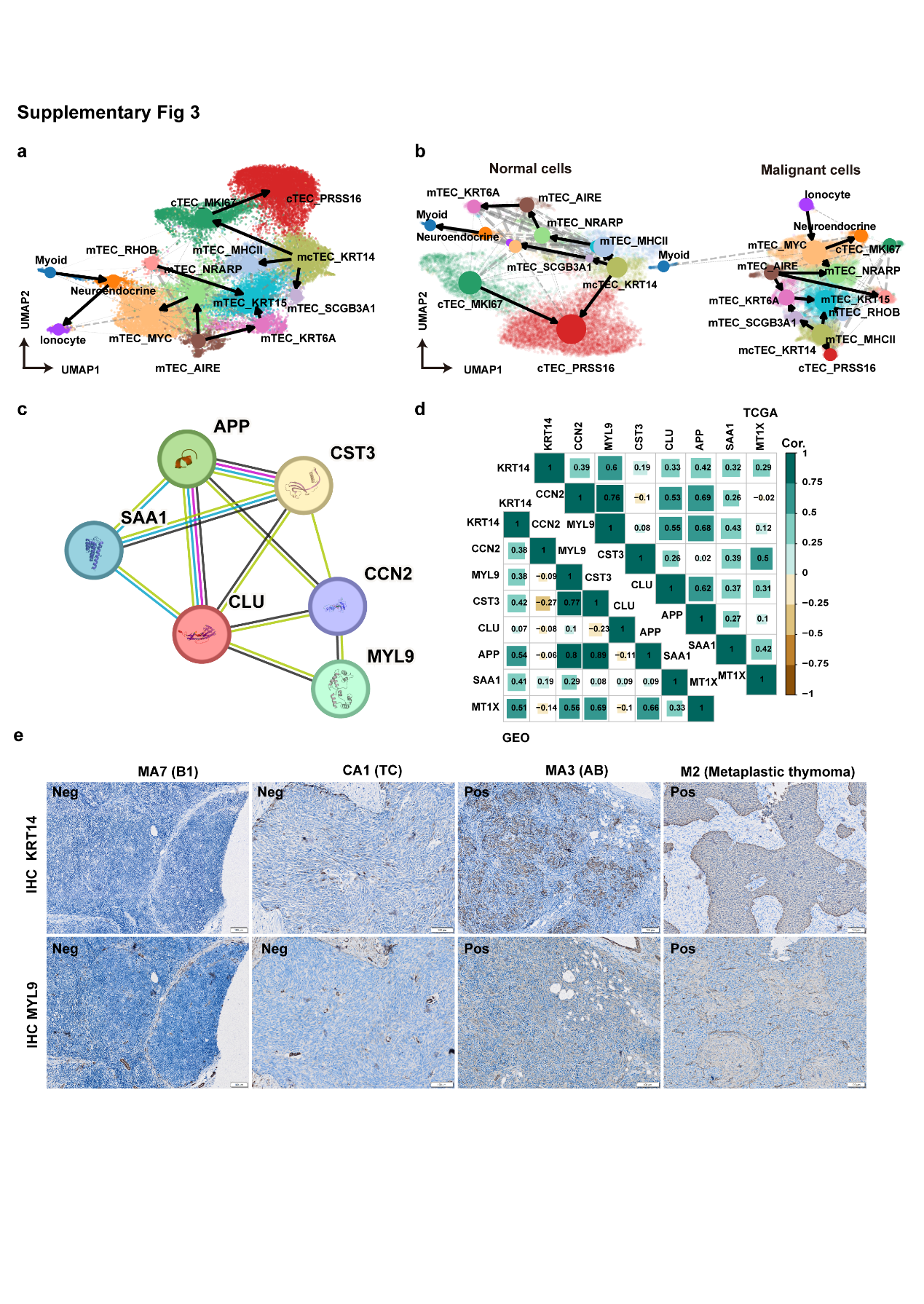
**

**Figure S3.** **Characterization of thymic epithelial progenitor cells**

a, UMAP showing the RNA velocity and PAGA of 14 epithelial cell subtypes of all cells.

b, UMAP showing the RNA velocity and PAGA of 14 epithelial cell subtypes of normal cells (left panel) and mailgnant cells (right panel).

c, APP, MYL9, CCN2, CLU, CST3, and SAA1 in a protein-protein interaction (PPI) network.

d, Heatmap showing the correlation of the indicated genes in the TCGA-THYM and GEO datasets.

e, IHC staining images showing KRT14 and MYL9 expression in patients MA7, CA1, MA3, and M2.

**
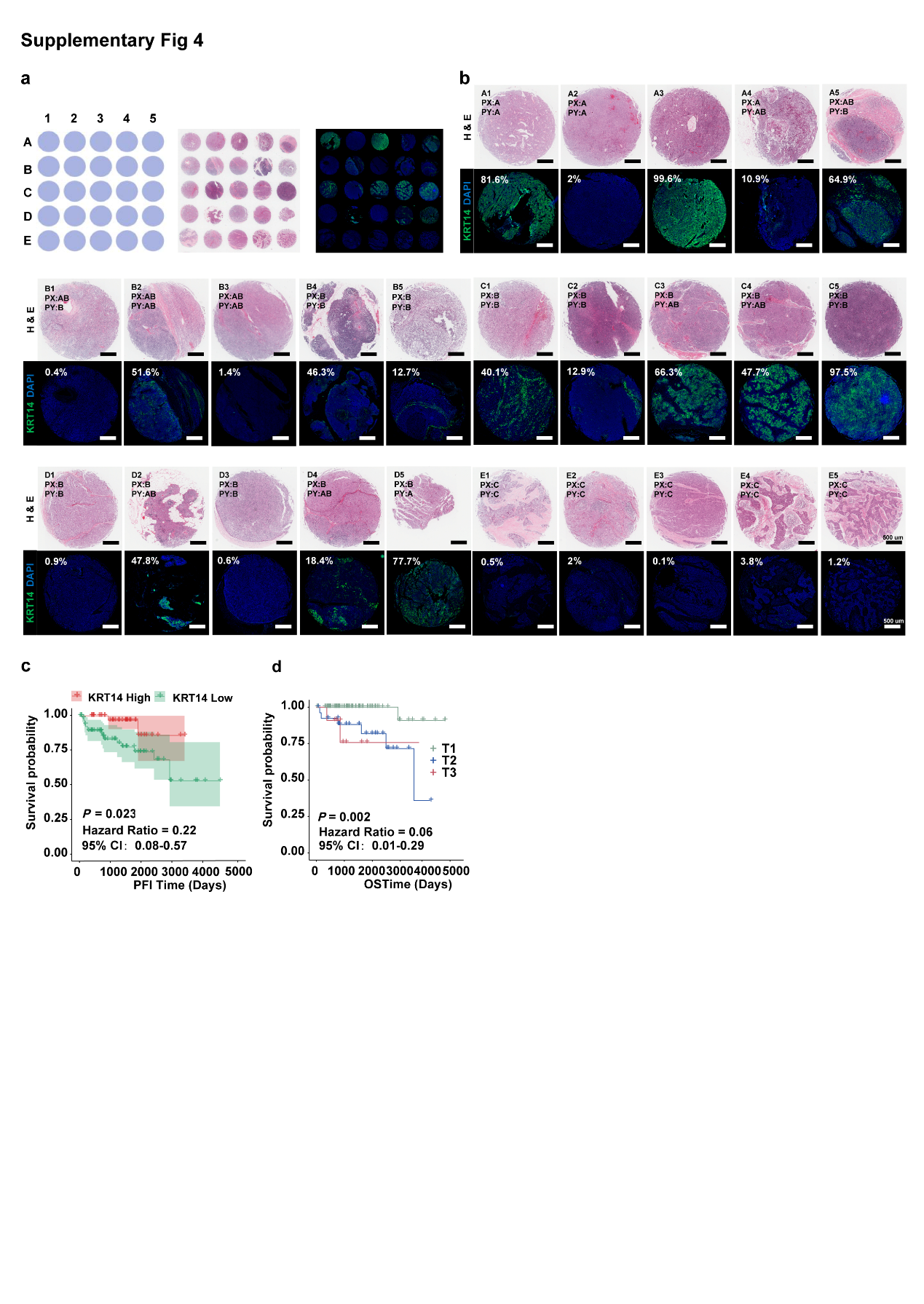
**

**Figure S4. Immunohistochemistry analysis of KRT14 expression in TET tissue**

a-b, mIF staining of KRT14 expression in 25 TM patients, KRT14 (green), and DAPI (blue). Scale bars, 200 μm.

c, Kaplan-Meier estimation of progression-free interval (PFI) and disease-specific survival (DSS) time in TM patients based on the expression levels of KRT14.

d, Kaplan-Meier estimation of overall survival time in TM patients according to the different subtypes. HRs and 95% CIs were calculated based on a Cox regression model (two-sided P value by log-rang test)

**
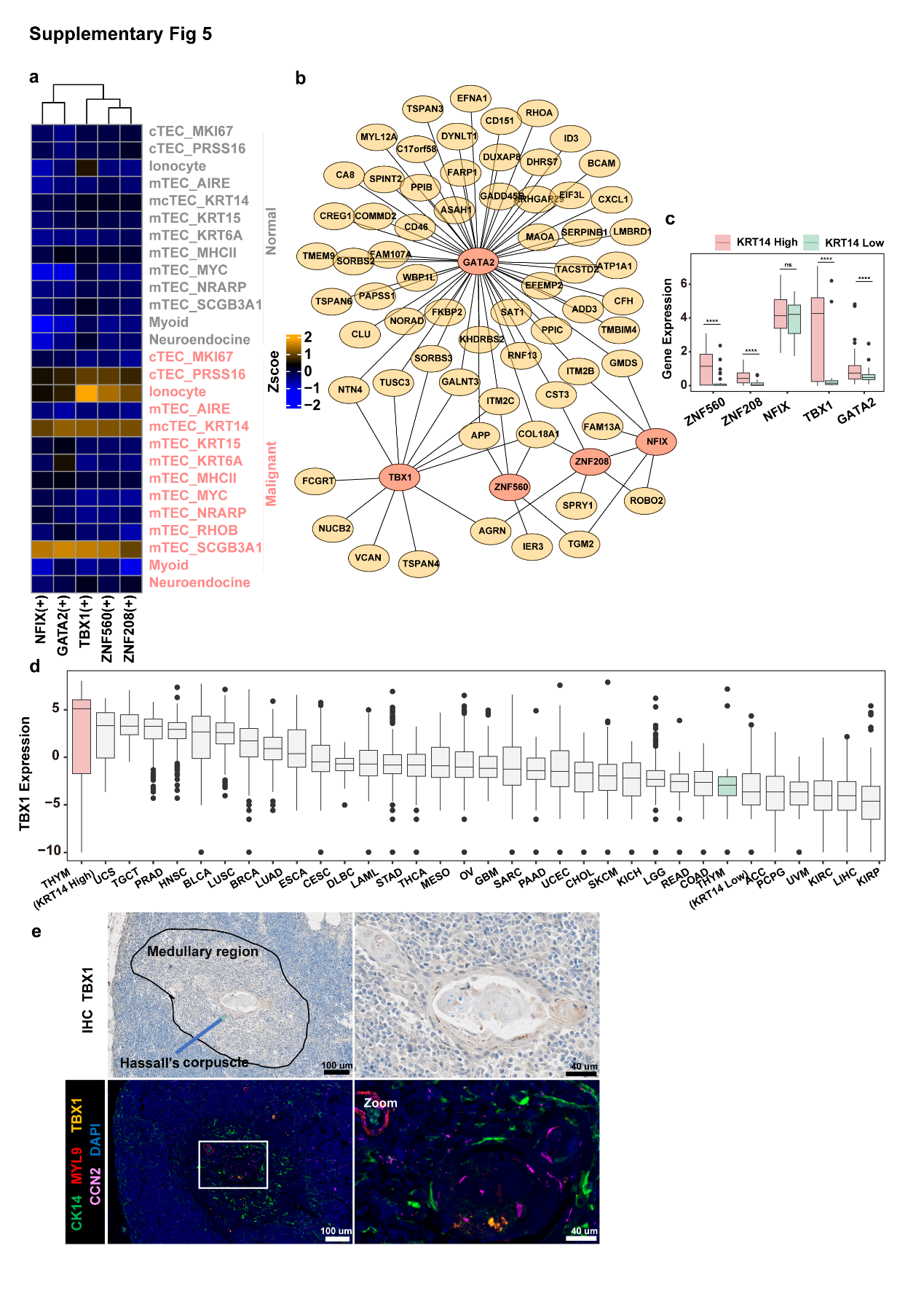
**

**Figure S5. Transcription patterns and master TFs of thymic epithelial progenitor cells**

a, Heatmap showing the activity of regulatory TFs across 12 malignant or normal epithelial cell subtypes.

b, Regulatory network showing master TFs (NFIX, GATA2, TBX1, ZNF560, and ZNF208) and their target genes in epithelial cells.

c, Box plot showing the expression levels of NFIX, GATA2, TBX1, ZNF560, and ZNF208 in high- and low- KRT14 groups. P values were calculated using a two-sided paired Wilcoxon signed-rank test. ns, *P* > 0.05 and ****, *P* < 0.0001.

d, Box plot showing TBX1 expression in 33 cancer types in the TCGA dataset.

e, H&E staining images showing TBX1 in the medulla region, and white Hassall’s corpuscle structure. mIF staining indicates the KRT14 (green), MYL9 (red), TBX1 (orange), CCN2 (pink), and DAPI (nuclei; blue) in the TET tissues. Scale bar, 50 μm; inset, 40 μm.

**
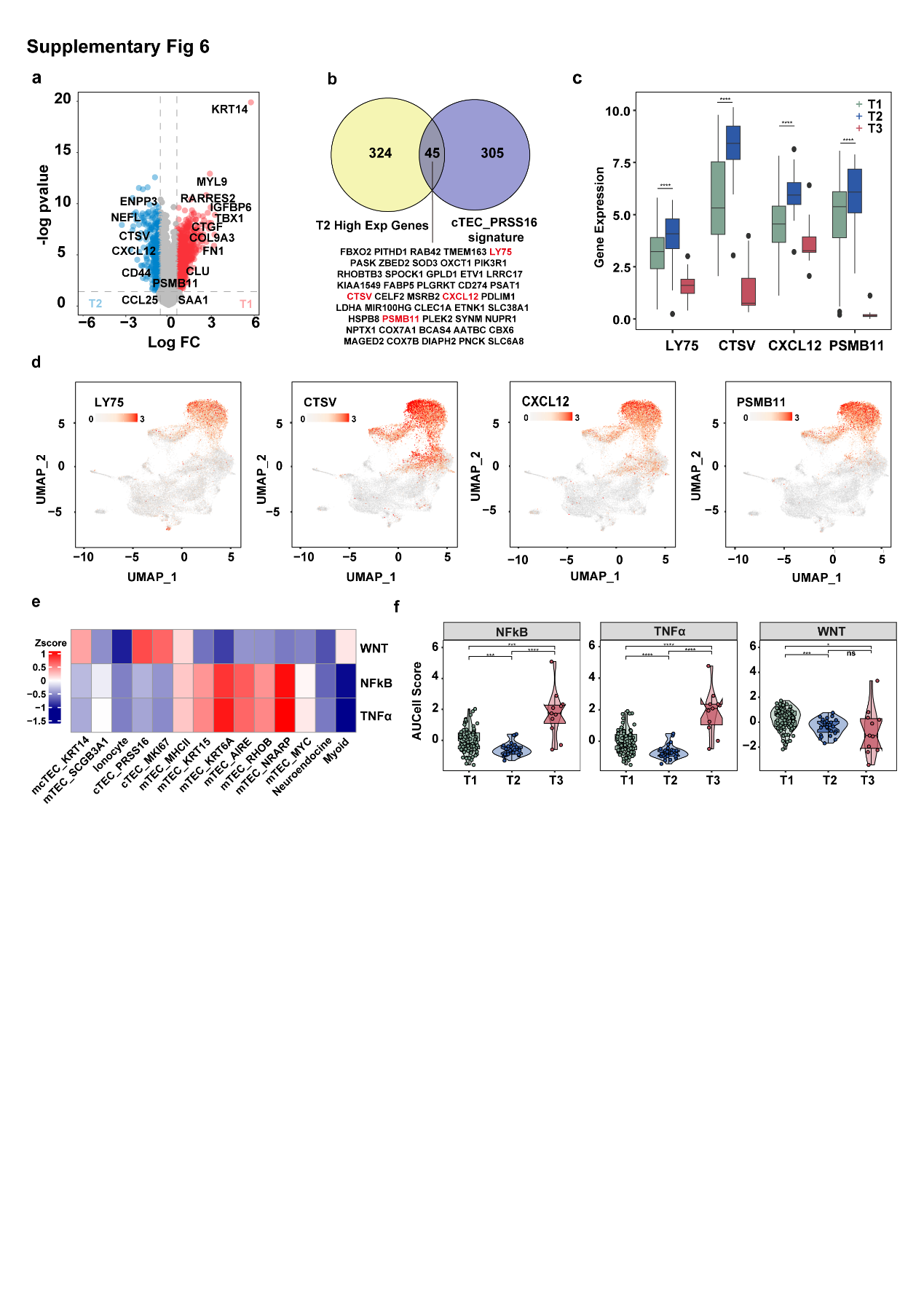
**

**Figure S6. Characterization of the T2 subtype**

a, Volcano plots showing differentially expressed genes between the T1 and T2 subtypes.

b, Venn diagram showing the overlapping genes in the T2 high expression genes and in the cTEC_PRSS16 cluster.

c, Box plot showing the expression levels of LY75, CTSV, CXCL12, and PSMB11 in the indicated subtypes in the TCGA dataset. P values were calculated using a two-sided paired Wilcoxon signed-rank test. ****, *P* < 0.0001.

d, UMAP plot showing the expression levels of LY75, CTSV, CXCL12, and PSMB11, as defined for all epithelial cells.

e, Heatmap showing the WNT, NFκB, and TNFα pathways in the 14 epithelial cell subtypes in TETs. The color intensity represents the scaled pathway enriched score, as calculated by AUCell.

f, Box plot showing the enriched score of WNT, NFκB, and TNFα pathways in the indicated subtypes in the TCGA dataset. The enriched score was calculated using AUCell.

**
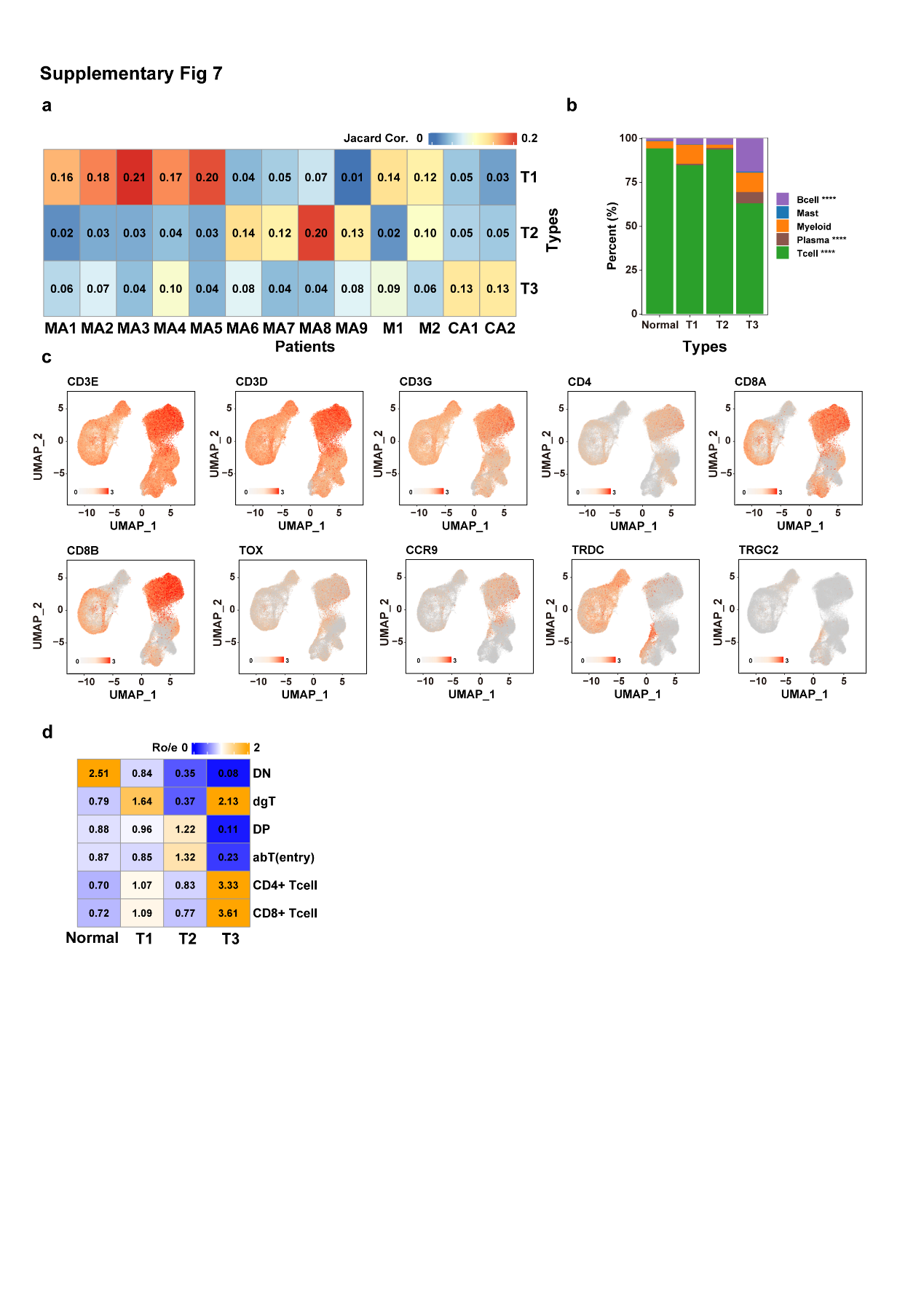
**

**Figure S7. Landscape of T cells in the normal and tumoral thymus**

a, Heatmap showing the Jaccard correlation between the indicated subtypes and each patient. The different expression genes in the three subtypes were used to characterize the subtypes. The differentially expressed genes from each patient’s malignant epithelial cells were used to characterize each patient.

b, Bar plot showing the percentage of immune cells in normal tissue and the T1, T2, and T3 subtypes. Chi square analysis calculated the infiltration differences of various immune subgroups in the T3 subtype compared to other patients. ****, *P* < 0.0001.

c, UMAP plot showing the expression levels of the selected markers in all cells.

d, Heatmap showing the subtype distribution of different T cells, as estimated by the Ro/e score.

**
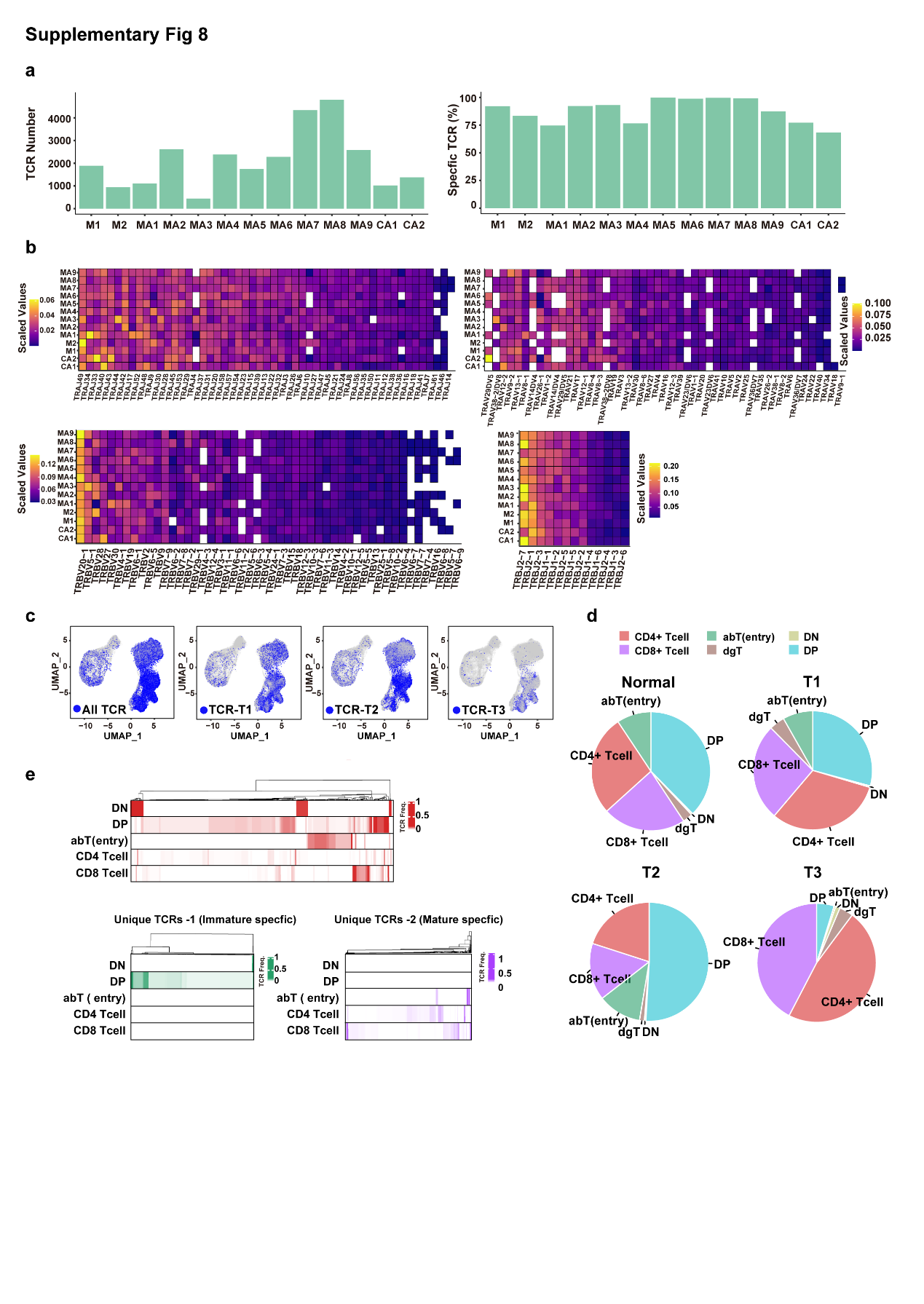
**

**Figure S8.** **VJ gene usage and pairing and TCR diversity in TETs**

a, Bar chart showing the number of TCRs and percent of specific TCRs in each patient.

b, Heatmap showing the scaled value of each TCRβ V and J gene segment present in the total T cells from each patient, normalized per column. The gene segments are positioned according to their genomic location.

c, UMAP showing the all cells, the T1 subtype cells, the T2 subtype cells, and the T3 subtype cells, according to their TCR sequence.

d, Pie charts showing the proportion of the different cell subtypes across normal tissues and the T1, T2, and T3 subtypes. Analysis was performed only on cells with TCR sequences.

e, Heatmap showing the frequency of each TCR in the five T cell development states. All TCRs in the expanded T cells from tumors are presented. The columns represent the different clonotypes, while the rows represent the T cell developmental states. The color key indicates the frequency of each TCR in every state. All TCRs clustered into shared-TCRs (TCR shared in immature cells and mature cells), unique TCRs-1 (TCRs only found in immature cells) and unique TCRs-2 (TCRs only found in mature cells).

**
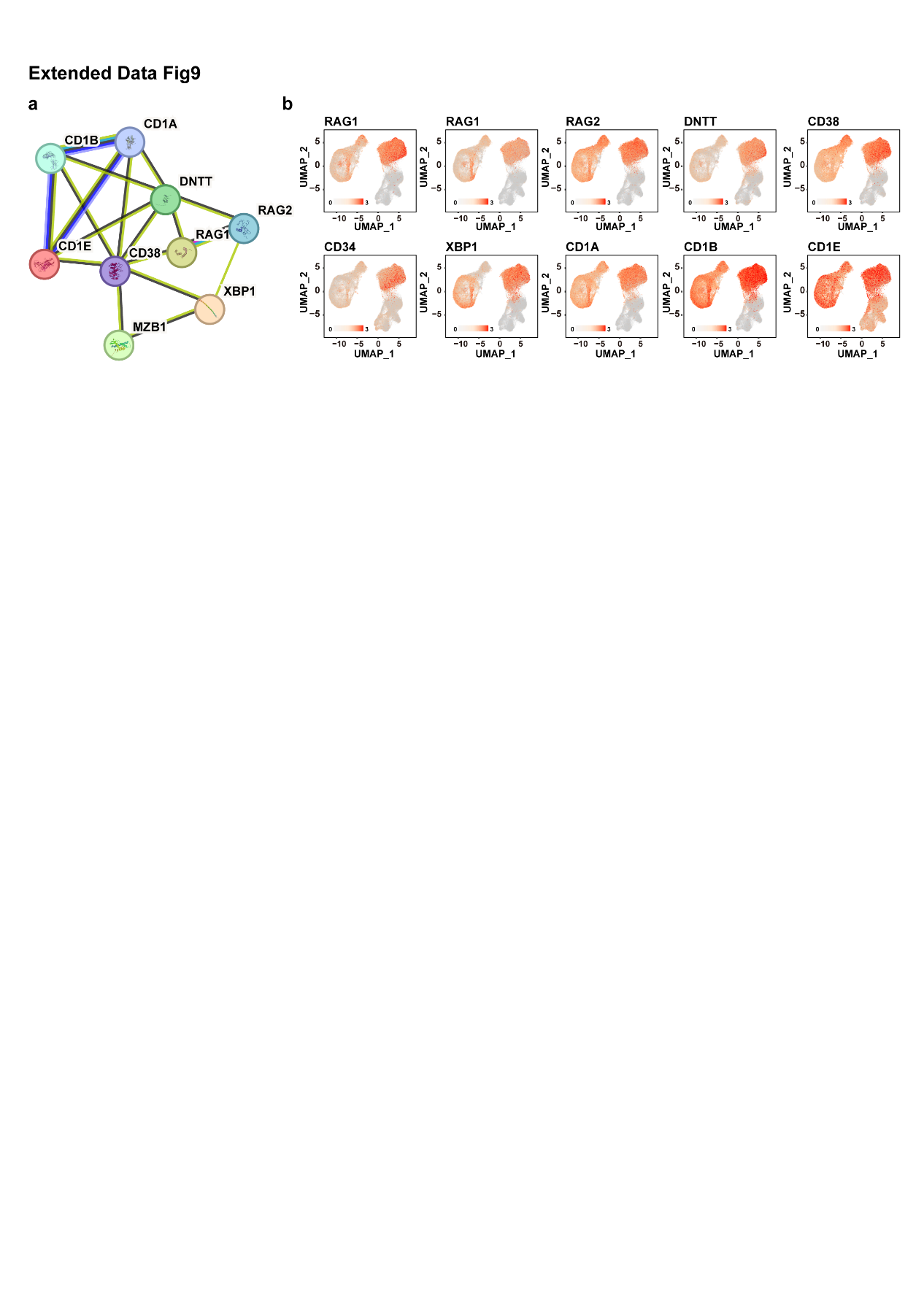
**

**Figure S9.** **Genes involved in TCR rearrangement**

a, Upregulated genes in TCR rearrangement in a protein-protein interaction (PPI) network.

b, UMAP plot showing the expression levels of the genes upregulated in TCR rearrangement in all T cells.

**
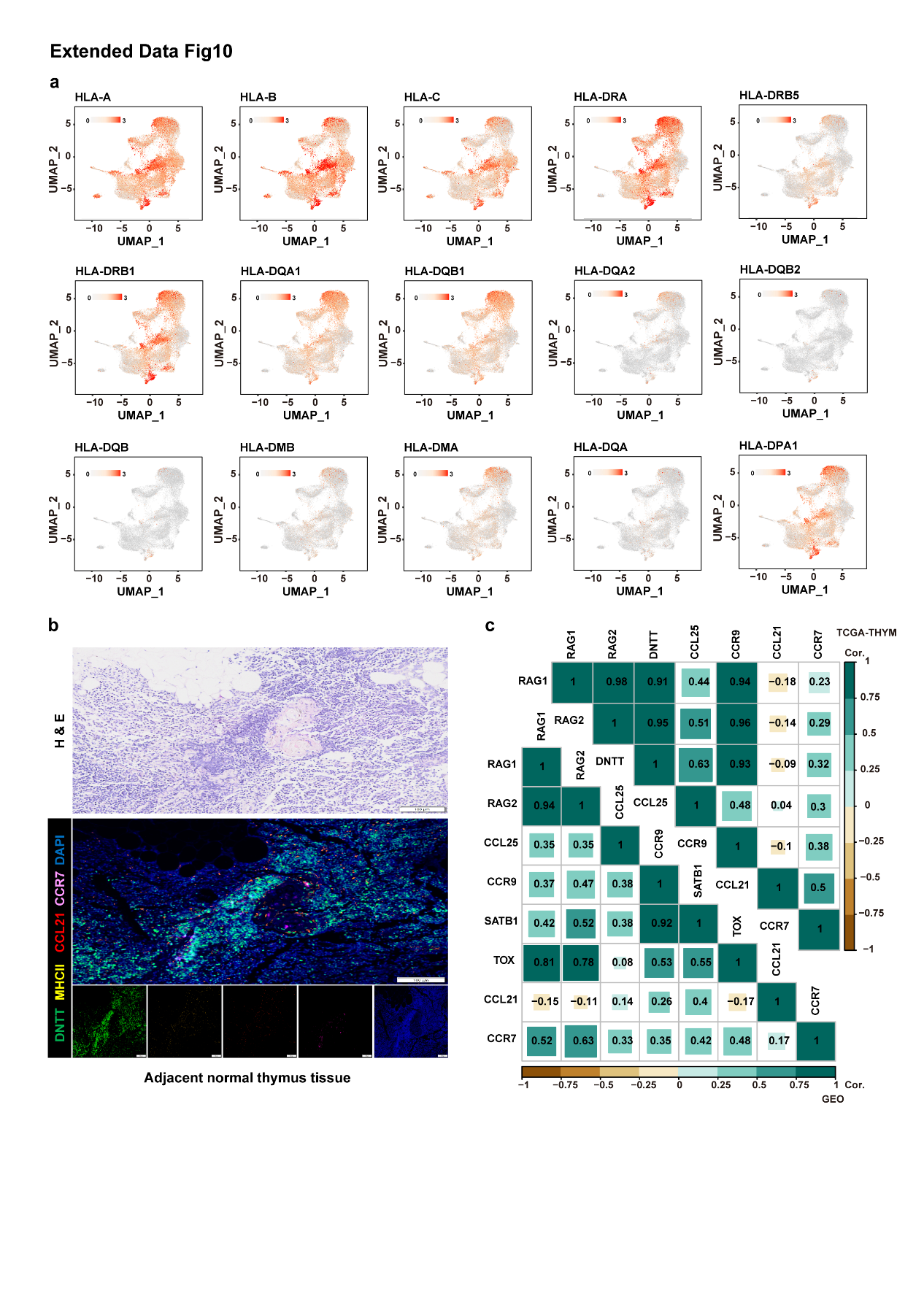
**

**Figure S10. Loss of CCL21^+^ mTEC in TM**

a, UMAP plot showing the expression levels of MHC genes in all epithelial cells.

b, H&E-stained sections showing the morphology of the cortical region and medullary region (top). mIF staining showing DNTT (green), MHCII (yellow), CCL21 (red), CCR7 (purple), and DAPI (nuclei; blue) in adjacent normal thymus tissue (bottom). Scale bar, 100 μm.

c, Heatmap showing the correlation of selected genes in the TCGA-THYM and GEO datasets.

**
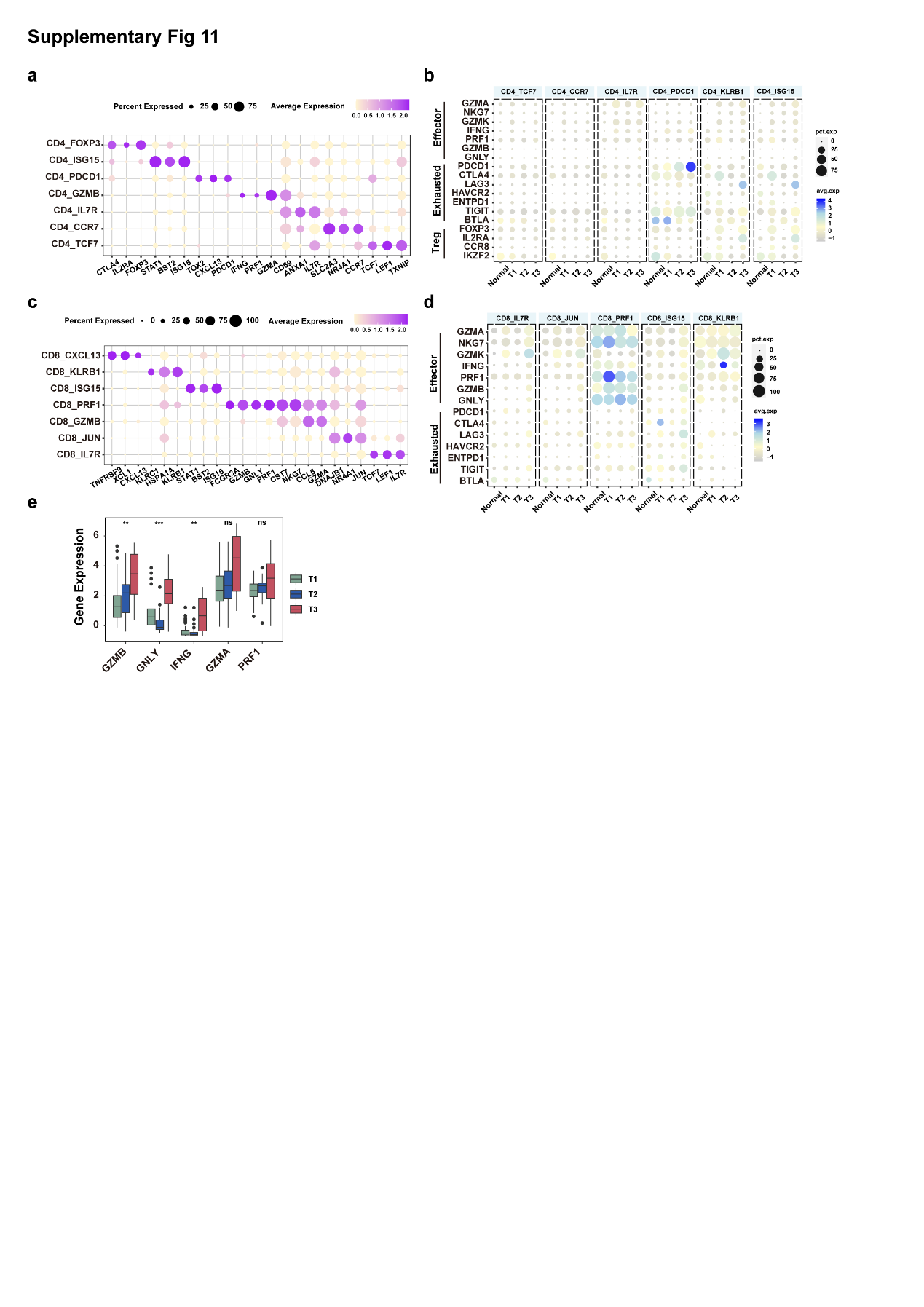
**

**Figure S11. TET Immune cell landscape**

a, Expression levels of the selected markers in different CD4^+^ T cell subpopulations. Dot size indicates the fraction of expressing cells and the colors represent the normalized gene expression levels.

b, Dot plots showing the expression levels in Tregs and exhausted and effector T cell marker genes across the CD4_TCF7, CD4_CCR7, CD4_IL7R, CD4_PDCD1 CD4_KLRB1, and CD4_ISG15 clusters in normal patient tissue and the three subtypes.

c, Expression levels of the selected markers in different CD8^+^ T cell subpopulations. Dot size indicates the fraction of expressing cells and the colors represent normalized gene expression levels.

d, Dot plots showing the expression level of exhausted and effector T cell markers gene across CD8_IL7R, CD8_JUN, CD8_PRF1, CD8_ISG15 and CD8_KLRB1 clusters in patients from normal and three subtypes

e, Box plots showing the expression levels of GZMB, GNLY, INFG, GZMA, and PRF1 in T1, T2, and T3 subtype patients. ns, *P* > 0.05; **, *P* < 0.01; and ***, *P* < 0.001, as determined by one-way ANOVA.

**
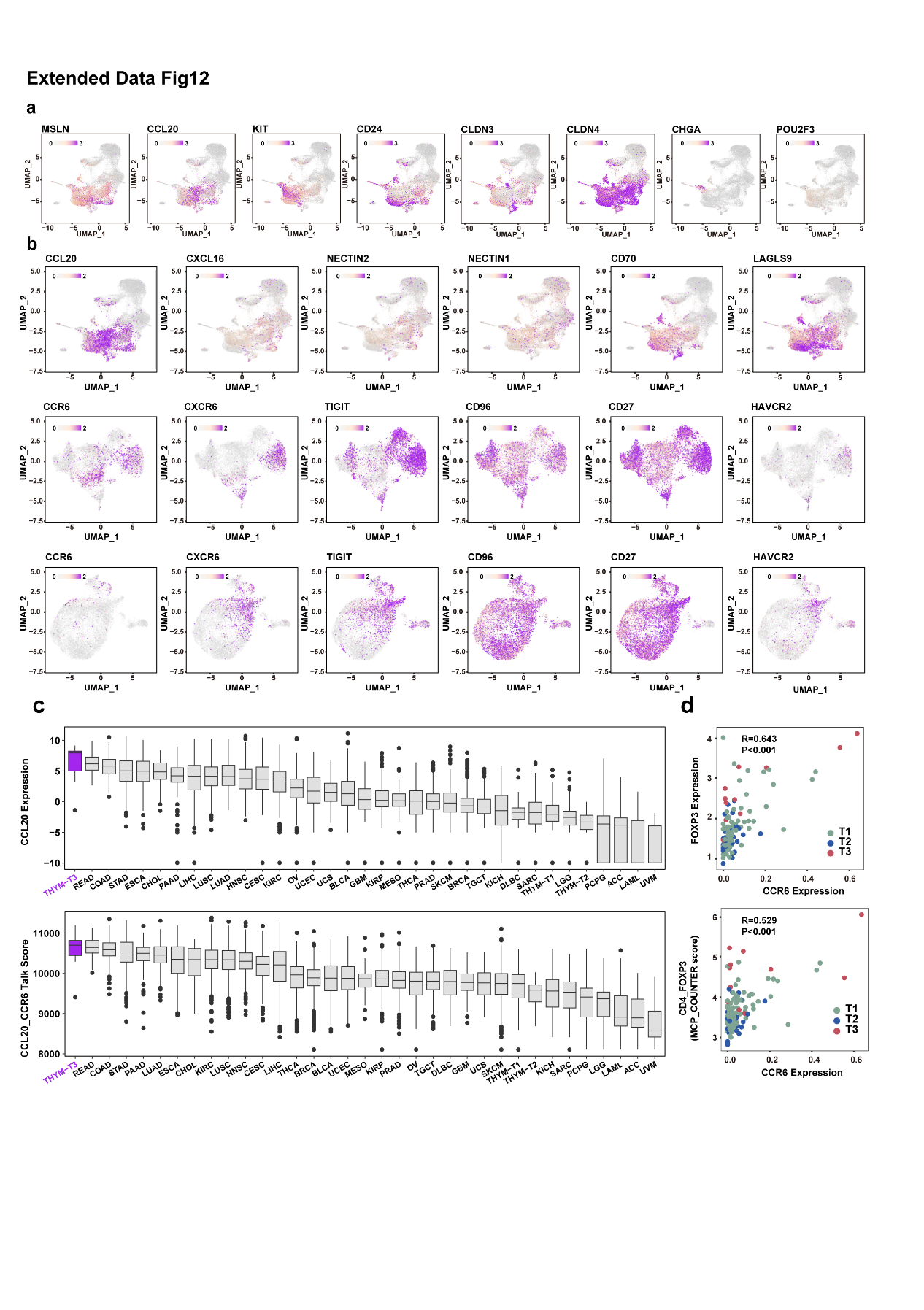
**

**Figure S12. The CCL20-CCR6 ligand-receptor pair promotes malignant cell recruitment of Treg cells in the T3 subtype.**

a, UMAP plot showing the expression levels of 8 most common upregulated signatures in T3 patients from the TCGA dataset in all epithelial cells.

b, UMAP plot showing the expression levels of ligand-receptor pairs specifically involved in cell-cell communication between malignant cells in T3 subtypes and T cells in all epithelial cells, all CD4^+^ T cells, and all CD8^+^ T cells.

c, Box plot showing CCL20 expression (top) and the CCL20-CCR6 crosstalk score (bottom) in 32 cancer types and three different subtypes in the TCGA-THYM dataset.

d, Scatter plot showing the correlation between CCR6 expression and FOXP3 expression in the TCGA dataset (top). Scatter plot showing the correlation between CCR6 expression and the CD4_FOXP3 infiltration score in the TCGA dataset (bottom). CD4_FOXP3 infiltration fraction was calculated using MCP_counter with the differentially expressed genes (logFC > 0.58 and *P* value < 0.05) in the CD4_FOXP3 subpopulation.


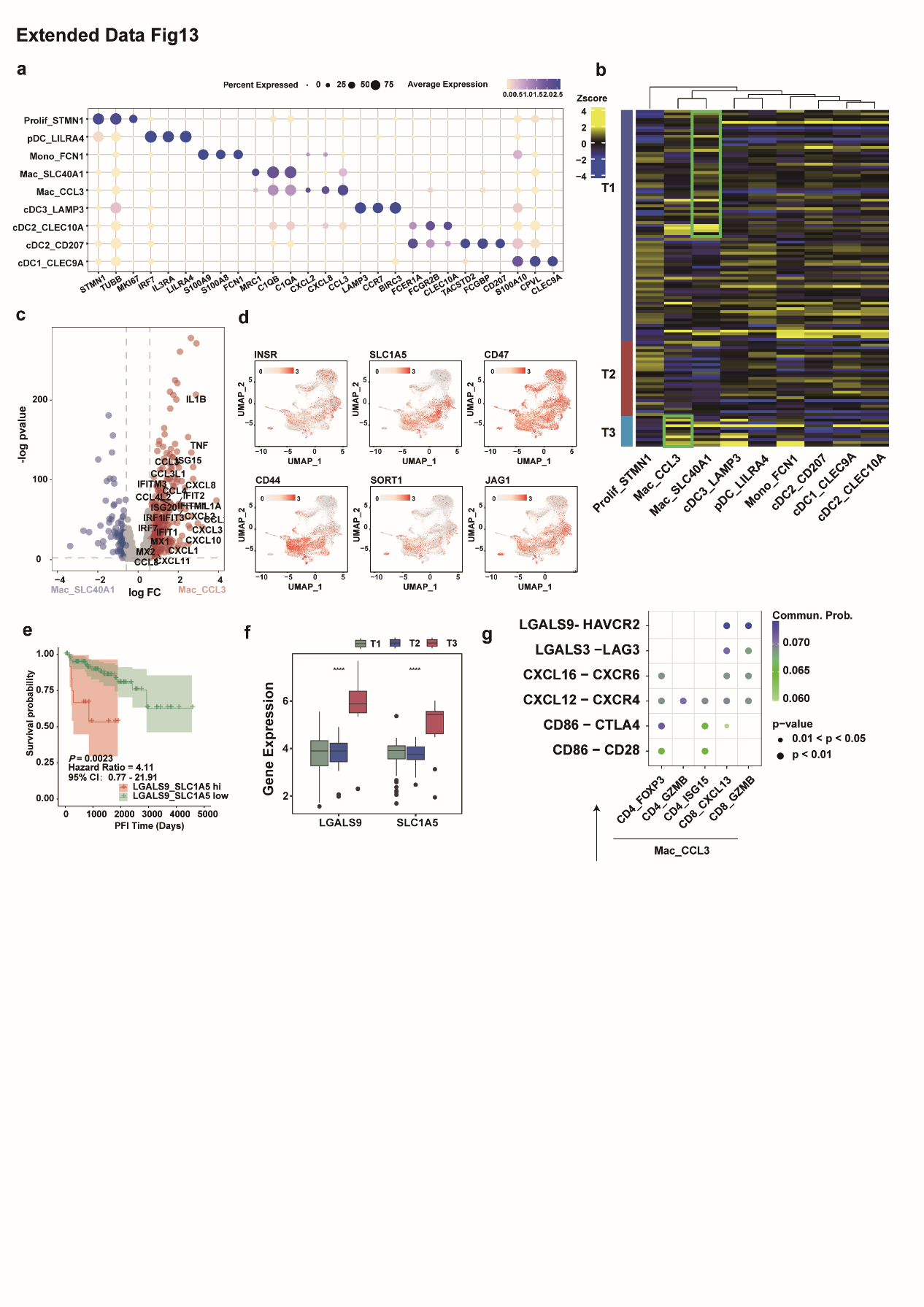


**Figure S13. The LGALS9-SLC1A5 ligand-receptor pair in cell-cell communication between malignant cells and myeloid cells**

a, Expression levels of the selected markers in different myeloid subpopulations. Dot size indicates the fraction of expressing cells and the color represents normalized gene expression levels.

b, Heatmap showing the infiltration score of each myeloid subpopulation in the three TET subtypes in the TCGA dataset. Each subpopulation’s infiltration score was calculated using MCP_counter with the differentially expressed genes (logFC > 0.58 and *P* values < 0.05) in each subcluster.

c, Volcano plot showing the differentially expressed genes between the Mac_SLC40A1 and Mac_CCL3 subpopulations.

d, UMAP plot showing the expression levels of INSR, SLC1A5, CD47, CD44, SORT1, and JAG1 in all epithelial cells.

e, Kaplan-Meier estimation of PFI time in TETs by the expression level of the LGALS9_SLC1A5 ligand-receptor pair. HRs and 95% CIs were calculated based on a Cox regression model (two-sided P value by log-rank test).

f, Box plots showing the expression level of LGALS9 and SLC1A5 in T1, T2 and T3 subtype patients. *****P* < 0.0001 were determined by one-way ANOVA test.

g, Dot plot showing the selected ligand-receptor pairs between cells in Mac_CCL3 and 5 T3 enriched T cell subpopulations. P values were determined using a one-sided permutation test.

**
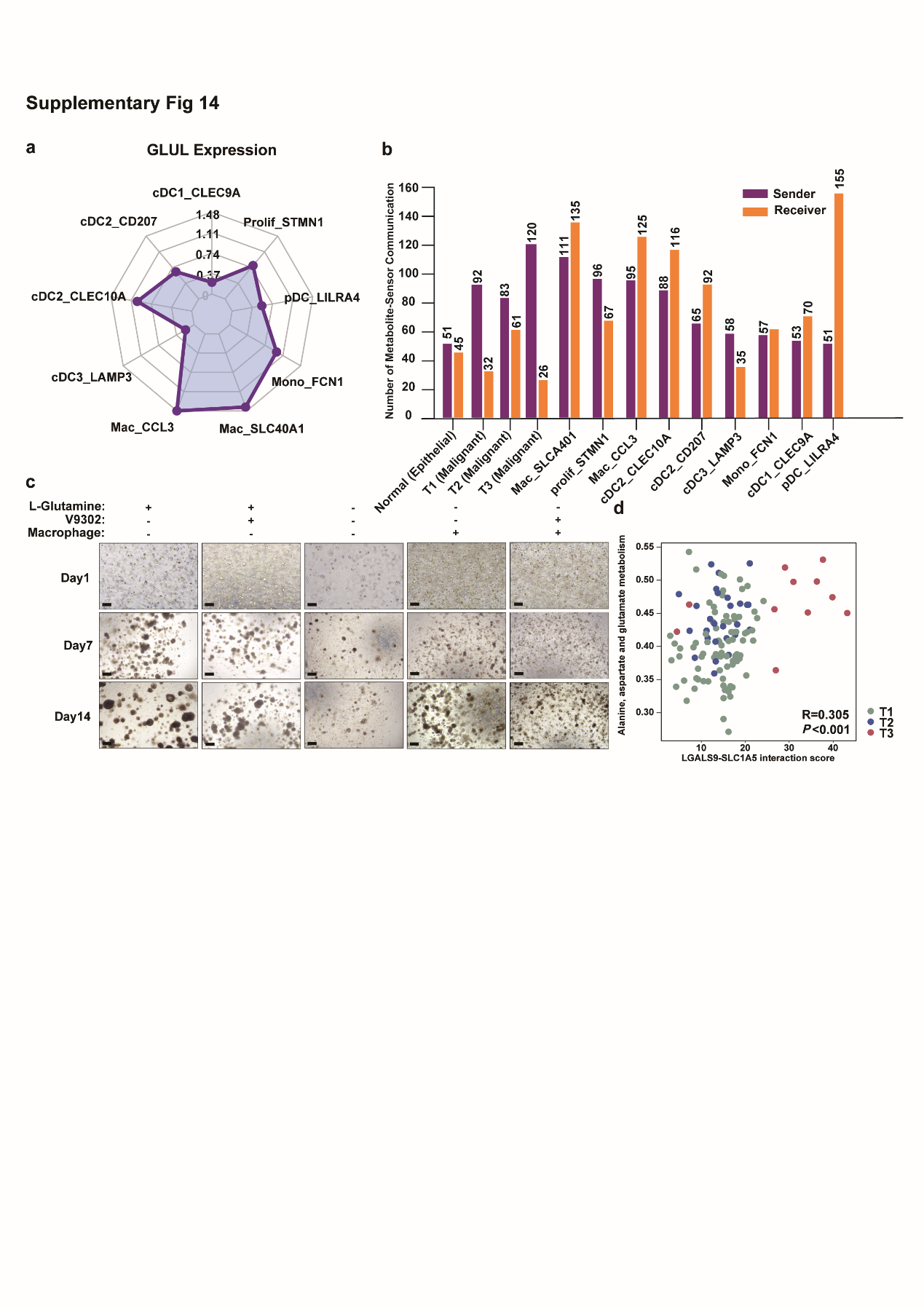
**

**Figure S14.** **Macrophage released glutamine provides energy for malignant cells**

a, Radar plots showing the expression levels of GLUL in 9 myeloid subpopulations.

b, Bar graph showing the sender-receiver scores, as determined using MEBOCOST. The myeloid subpopulations and cancer cell subtypes are along the x-axis is the while the y-axis is the communication score. The orange and purple bars indicate the number of communications for sender and receiver cells, respectively.

c, Representative brightfield microscopy images of TM organoids treated with V-9302, a glutamine transporter ASCT2 (SLC1A5) inhibitor. Scale bar, 100 µm.

d, Scatter plot showing the correlation between the LGALS9-SLC1A5 interaction score and the Alanine, aspartate and glutamate metabolism score. The Alanine, aspartate and glutamate metabolism score was calculated using ssGSEA with this pathway’s genes (logFC > 0.58 and P < 0.05).

**
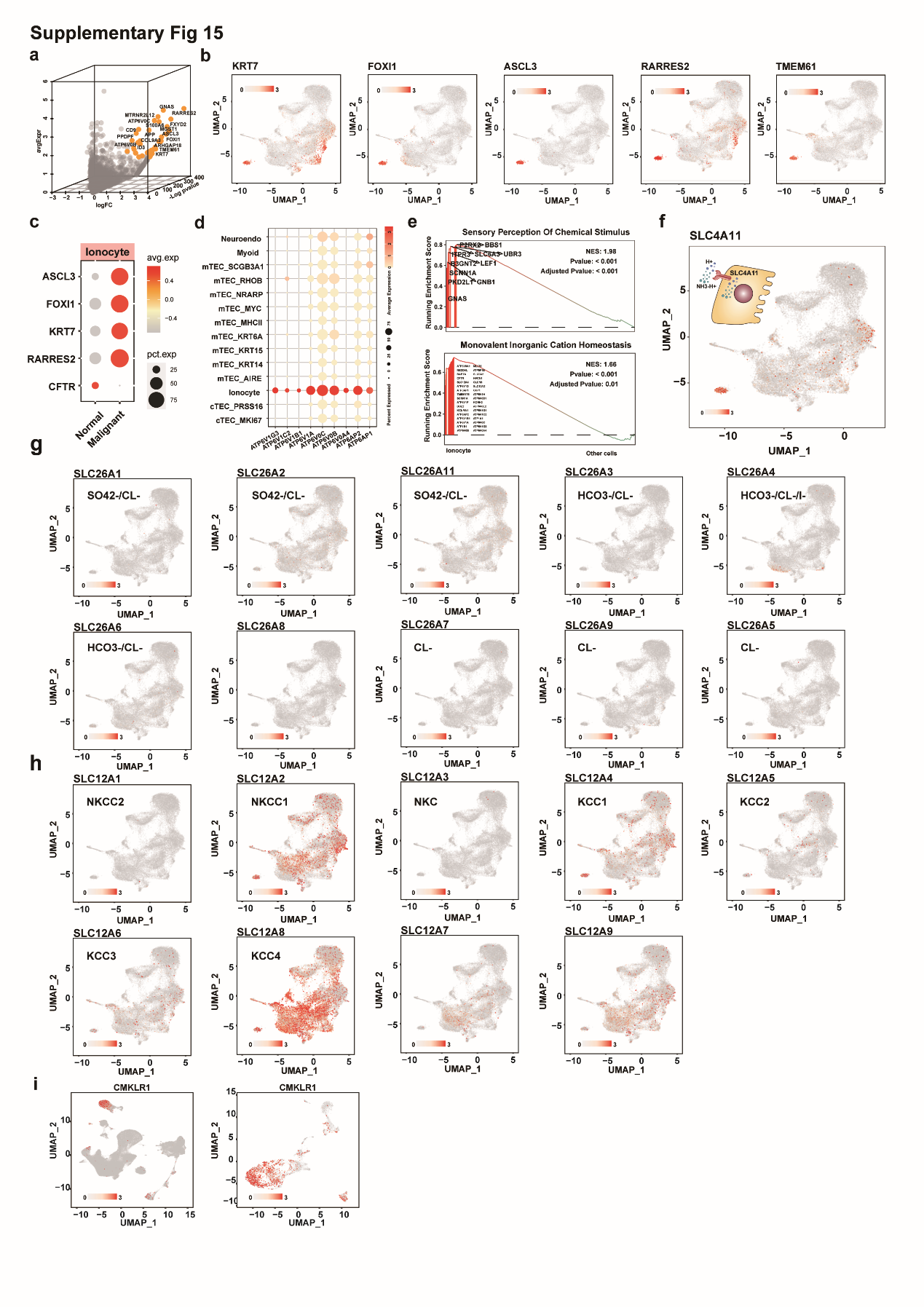
**

**Figure S15. Ionocytes with upregulated SLC4A11 have H^+^ transporter functions.**

a, Three-dimensional plot showing the genes elevated expression in ionocytes.

b, UMAP plot showing the expression levels of KRT7, FOXI1, ASCL3, RARRES2, and TMEM61 in all epithelial cells.

c, The expression level of ASCL3, FOXI1, KRT7, RARRES2, and CFTR in normal ionocytes and malignant ionocytes. Dot size indicates the fraction of expressing cells and the color represents normalized expression levels.

d, ATPase gene expression levels in the indicated epithelial cell subpopulations. Dot size indicates the fraction of expressing cells and the colors represent normalized gene expression levels.

e, Gene set enrichment analysis showing the upregulation of chemical stimulus sensory perception and monovalent inorganic cation homeostasis pathways in ionocytes. The normalized enrichment score (NES) and false discovery rate (FDR) are included.

f, UMAP plot showing the expression levels of SLC4A11 in all epithelial cells.

g-h, UMAP plot showing the expression levels of SLC26 anion transporter family (f) and SLC12 family (g) genes in all epithelial cells.

i, UMAP plot showing the expression levels of CMKLR1 in all cells and all myeloid cells.

**Supplementary Tables**

Table S1. Clinical characteristics of patients for single-cell RNA sequencing

| **Sample** | **Dataset** | **Sorting** | **Tissue** | **Age** | **Gender** | **TCR** | **Site** | **WTO** | **Masaoka** |
| --- | --- | --- | --- | --- | --- | --- | --- | --- | --- |
| N1 | Jong-Eun Park et al | All | Normal | 9w | Female | × | thymus | Normal | Normal |
| N2 | Jong-Eun Park et al | All | Normal | 7W | Female | × | thymus | Normal | Normal |
| N3 | Jong-Eun Park et al | All | Normal | 8W | Male | × | thymus | Normal | Normal |
| N4 | Inhouse | All | Normal | Unknown | Male | × | thymus | Normal | Normal |
| N5 | Jhoanne L. Bautista et al | CD45- | Normal | 19W | Unknown | × | thymus | Normal | Normal |
| N6 | Jong-Eun Park et al | All | Normal | 3M | Male | × | thymus | Normal | Normal |
| N7 (T7 Peritumor) | Inhouse | All | Normal | 67 | Female | × | thymus | Normal | Normal |
| MA1 | Zhongwei Xin et al | All | Primary Tumor | 72 | Female | √ | thymus | A | I |
| MA2 | Inhouse | All | Primary Tumor | 64 | Female | √ | thymus | AB | Unknown |
| MA3 | Inhouse | All | Primary Tumor | 26 | Female | √ | thymus | AB | Unknown |
| MA4 | Inhouse | All | Primary Tumor | 57 | male | √ | thymus | AB | Unknown |
| MA5 | Zhongwei Xin et al | All | Primary Tumor | 56 | Male | √ | thymus | AB | I |
| MA6 | Zhongwei Xin et al | All | Primary Tumor | 55 | Male | √ | thymus | AB | I |
| MA7 | Inhouse | All | Primary Tumor | 67 | Female | √ | thymus | B1 | III |
| MA8 | Inhouse | All | Primary Tumor | 46 | Male | √ | thymus | B2 | IA |
| MA9 | Zhongwei Xin et al | All | Primary Tumor | 44 | Male | √ | thymus | B3 | III |
| M1 | Zhongwei Xin et al | All | Primary Tumor | 67 | Female | √ | thymus | MNT | IIA |
| M2 | Inhouse | All | Primary Tumor | 51 | Female | √ | thymus | Metaplastic thymoma | II |
| CA1 | Inhouse | All | Primary Tumor | 44 | Male | √ | thymus | TC | cT1N0M0 |
| CA2 | Zhongwei Xin et al | All | Primary Tumor | 69 | Male | √ | thymus | TC | III |

Table S2. Somatic mutations detected in the tumor tissues from four patients by whole-exome sequencing.

| Start_Position | End_Position | Variant_Classification | Variant_Type | Reference_Allele | Tumor_Seq_Allele1 | Tumor_Seq_Allele2 | Ref_allele_depth | Alt_allele_depth | VAF | CDS_Change | Protein_Change | Tumor_Sample_Barcode |
| --- | --- | --- | --- | --- | --- | --- | --- | --- | --- | --- | --- | --- |
| 2563346 | 2563346 | 3'UTR | SNP | G | G | A | 80 | 4 | 0.04 | NA | NA | MA2 |
| 10948083 | 10948083 | Silent | SNP | T | T | A | 197 | 5 | 0.02 | c.A2052T#GGA->GGT#G->G# | p.G684G | MA2 |
| 16619939 | 16619939 | RNA | SNP | C | C | T | 17 | 4 | 0.19 | NA | NA | MA2 |
| 16630060 | 16630060 | RNA | SNP | C | C | T | 37 | 12 | 0.24 | NA | NA | MA2 |
| 16633495 | 16633495 | IGR | SNP | C | C | T | 46 | 15 | 0.24 | NA | NA | MA2 |
| 23323811 | 23323811 | Intron | SNP | A | A | T | 177 | 8 | 0.04 | NA | NA | MA2 |
| 32673444 | 32673444 | Intron | SNP | T | T | C | 106 | 9 | 0.07 | NA | NA | MA2 |
| 43579088 | 43579088 | Intron | SNP | G | G | A | 232 | 5 | 0.02 | NA | NA | MA2 |
| 52041719 | 52041719 | Intron | SNP | T | T | C | 50 | 52 | 0.5 | NA | NA | MA2 |
| 76699920 | 76699920 | IGR | SNP | G | G | C | 104 | 17 | 0.14 | NA | NA | MA2 |
| 76700171 | 76700171 | IGR | SNP | C | C | T | 205 | 7 | 0.03 | NA | NA | MA2 |
| 94901747 | 94901747 | Silent | SNP | T | T | G | 163 | 6 | 0.03 | c.A285C#GGA->GGC#G->G# | p.G95G | MA2 |
| 1.45E+08 | 1.45E+08 | IGR | SNP | T | T | A | 63 | 7 | 0.1 | NA | NA | MA2 |
| 1.6E+08 | 1.6E+08 | RNA | SNP | C | C | T | 115 | 9 | 0.07 | NA | NA | MA2 |
| 1.6E+08 | 1.6E+08 | RNA | SNP | T | T | C | 114 | 9 | 0.07 | NA | NA | MA2 |
| 1.63E+08 | 1.63E+08 | 5'UTR | SNP | C | C | A | 24 | 37 | 0.6 | NA | NA | MA2 |
| 1.68E+08 | 1.68E+08 | Silent | SNP | G | G | A | 259 | 27 | 0.09 | c.C447T#GTC->GTT#V->V# | p.V149V | MA2 |
| 1.83E+08 | 1.83E+08 | Missense_Mutation | SNP | A | A | G | 229 | 17 | 0.06 | c.A2494G#AAT->GAT#N->D# | p.N832D | MA2 |
| 1.97E+08 | 1.97E+08 | Missense_Mutation | SNP | G | G | C | 87 | 21 | 0.19 | c.G523C#GAA->CAA#E->Q# | p.E175Q | MA2 |
| 2.37E+08 | 2.37E+08 | Intron | DEL | TA | TA | - | 118 | 10 | 0.07 | NA | NA | MA2 |
| 2.37E+08 | 2.37E+08 | Intron | SNP | TT | TT | AA | 118 | 10 | 0.07 | NA | NA | MA2 |
| 6883491 | 6883491 | Missense_Mutation | SNP | G | G | A | 120 | 11 | 0.08 | c.G467A#AGC->AAC#S->N# | p.S156N | MA2 |
| 24077739 | 24077739 | Nonsense_Mutation | SNP | G | G | T | 160 | 6 | 0.03 | c.C642A#TGC->TGA#C->*# | p.C214X | MA2 |
| 25427737 | 25427737 | Intron | SNP | C | C | T | 165 | 13 | 0.07 | NA | NA | MA2 |
| 58252117 | 58252117 | IGR | SNP | T | T | A | 59 | 9 | 0.13 | NA | NA | MA2 |
| 70088799 | 70088799 | Silent | SNP | C | C | G | 86 | 4 | 0.04 | c.C1056G#GGC->GGG#G->G# | p.G352G | MA2 |
| 91941168 | 91941168 | RNA | SNP | C | C | T | 97 | 44 | 0.31 | NA | NA | MA2 |
| 1.06E+08 | 1.06E+08 | IGR | SNP | G | G | A | 70 | 9 | 0.11 | NA | NA | MA2 |
| 1.41E+08 | 1.41E+08 | Intron | SNP | G | G | A | 19 | 10 | 0.34 | NA | NA | MA2 |
| 1.41E+08 | 1.41E+08 | Intron | SNP | C | C | T | 19 | 22 | 0.53 | NA | NA | MA2 |
| 1.48E+08 | 1.48E+08 | nonframeshift substitution | SNP | CC | CC | GG | 335 | 6 | 0.01 | NA | NA | MA2 |
| 1.81E+08 | 1.81E+08 | Missense_Mutation | SNP | A | A | G | 154 | 12 | 0.07 | c.A458G#TAC->TGC#Y->C# | p.Y153C | MA2 |
| 2.02E+08 | 2.02E+08 | Intron | SNP | A | A | G | 38 | 3 | 0.07 | NA | NA | MA2 |
| 2.07E+08 | 2.07E+08 | Intron | SNP | A | A | T | 244 | 8 | 0.03 | NA | NA | MA2 |
| 31532962 | 31532962 | 5'UTR | SNP | C | C | A | 50 | 3 | 0.05 | NA | NA | MA2 |
| 57242296 | 57242296 | Intron | SNP | T | T | A | 92 | 5 | 0.05 | NA | NA | MA2 |
| 61742582 | 61742582 | Intron | SNP | G | G | T | 70 | 9 | 0.11 | NA | NA | MA2 |
| 61742663 | 61742663 | Intron | SNP | C | C | T | 105 | 14 | 0.11 | NA | NA | MA2 |
| 1.56E+08 | 1.56E+08 | IGR | SNP | T | T | C | 33 | 7 | 0.17 | NA | NA | MA2 |
| 1.57E+08 | 1.57E+08 | RNA | SNP | A | A | G | 24 | 6 | 0.2 | NA | NA | MA2 |
| 1.61E+08 | 1.61E+08 | RNA | SNP | A | A | G | 80 | 23 | 0.22 | NA | NA | MA2 |
| 1.61E+08 | 1.61E+08 | RNA | SNP | G | G | C | 94 | 23 | 0.19 | NA | NA | MA2 |
| 1.72E+08 | 1.72E+08 | IGR | SNP | G | G | A | 42 | 6 | 0.12 | NA | NA | MA2 |
| 1.76E+08 | 1.76E+08 | Intron | SNP | A | A | C | 71 | 6 | 0.07 | NA | NA | MA2 |
| 1.87E+08 | 1.87E+08 | IGR | SNP | T | T | C | 399 | 6 | 0.01 | NA | NA | MA2 |
| 53442720 | 53442721 | Intron | SNP | GG | GG | CC | 227 | 9 | 0.03 | NA | NA | MA2 |
| 99948422 | 99948422 | 3'UTR | SNP | G | G | A | 87 | 4 | 0.04 | NA | NA | MA2 |
| 1.73E+08 | 1.73E+08 | 3'UTR | SNP | T | T | A | 71 | 5 | 0.06 | NA | NA | MA2 |
| 40834502 | 40834502 | Silent | SNP | T | T | C | 159 | 6 | 0.03 | c.A108G#AAA->AAG#K->K# | p.K36K | MA2 |
| 55968551 | 55968551 | Intron | SNP | T | T | G | 72 | 7 | 0.08 | NA | NA | MA2 |
| 1.19E+08 | 1.19E+08 | Intron | SNP | G | G | C | 48 | 15 | 0.23 | NA | NA | MA2 |
| 1.19E+08 | 1.19E+08 | Intron | SNP | C | C | A | 54 | 21 | 0.28 | NA | NA | MA2 |
| 1.41E+08 | 1.41E+08 | Missense_Mutation | SNP | C | C | T | 134 | 6 | 0.04 | c.C2386T#CCC->TCC#P->S# | p.P796S | MA2 |
| 1.5E+08 | 1.5E+08 | 3'UTR | SNP | T | T | C | 309 | 14 | 0.04 | NA | NA | MA2 |
| 1.54E+08 | 1.54E+08 | RNA | SNP | G | G | A | 30 | 6 | 0.16 | NA | NA | MA2 |
| 29636335 | 29636335 | IGR | SNP | T | T | G | 63 | 11 | 0.14 | NA | NA | MA2 |
| 29942781 | 29942781 | Missense_Mutation | SNP | T | T | A | 48 | 8 | 0.14 | c.T98A#TTC->TAC#F->Y# | p.F33Y | MA2 |
| 29945567 | 29945567 | 3'UTR | SNP | G | G | T | 410 | 7 | 0.01 | NA | NA | MA2 |
| 29945567 | 29945567 | 3'UTR | SNP | G | G | A | 410 | 7 | 0.01 | NA | NA | MA2 |
| 30486014 | 30486014 | IGR | SNP | T | T | C | 44 | 12 | 0.21 | NA | NA | MA2 |
| 30486025 | 30486025 | IGR | SNP | A | A | G | 45 | 12 | 0.21 | NA | NA | MA2 |
| 31269123 | 31269123 | 3'UTR | SNP | G | G | A | 260 | 9 | 0.03 | NA | NA | MA2 |
| 31354249 | 31354249 | 3'UTR | SNP | C | C | A | 173 | 8 | 0.04 | NA | NA | MA2 |
| 32038419 | 32038419 | 5'UTR | SNP | C | C | T | 39 | 15 | 0.27 | NA | NA | MA2 |
| 32554712 | 32554712 | RNA | SNP | C | C | T | 60 | 11 | 0.15 | NA | NA | MA2 |
| 33069780 | 33069780 | Silent | SNP | C | C | T | 245 | 4 | 0.01 | c.G207A#AAG->AAA#K->K# | p.K69K | MA2 |
| 38677384 | 38677385 | Splice_Site | SNP | CT | CT | AG | 118 | 9 | 0.07 | NA | NA | MA2 |
| 72179939 | 72179939 | Intron | SNP | G | G | A | 171 | 5 | 0.02 | NA | NA | MA2 |
| 1.08E+08 | 1.08E+08 | IGR | SNP | C | C | T | 94 | 5 | 0.05 | NA | NA | MA2 |
| 1.09E+08 | 1.09E+08 | Intron | SNP | G | G | A | 125 | 4 | 0.03 | NA | NA | MA2 |
| 6502162 | 6502162 | Intron | SNP | A | A | T | 143 | 4 | 0.02 | NA | NA | MA2 |
| 56826392 | 56826392 | IGR | SNP | G | G | C | 18 | 7 | 0.28 | NA | NA | MA2 |
| 89334486 | 89334486 | Missense_Mutation | SNP | G | G | C | 220 | 13 | 0.05 | c.G1504C#GGT->CGT#G->R# | p.G502R | MA2 |
| 98386082 | 98386082 | Intron | SNP | G | G | C | 23 | 28 | 0.54 | NA | NA | MA2 |
| 1.01E+08 | 1.01E+08 | Intron | SNP | T | T | C | 79 | 20 | 0.2 | NA | NA | MA2 |
| 1.33E+08 | 1.33E+08 | Intron | SNP | T | T | C | 91 | 11 | 0.1 | NA | NA | MA2 |
| 1.38E+08 | 1.38E+08 | Intron | SNP | T | T | G | 19 | 29 | 0.6 | NA | NA | MA2 |
| 1.4E+08 | 1.4E+08 | Intron | SNP | A | A | C | 49 | 4 | 0.07 | NA | NA | MA2 |
| 1.4E+08 | 1.4E+08 | Intron | SNP | T | T | A | 55 | 4 | 0.06 | NA | NA | MA2 |
| 1.51E+08 | 1.51E+08 | Intron | SNP | TG | TG | CT | 81 | 3 | 0.03 | NA | NA | MA2 |
| 10529530 | 10529530 | Missense_Mutation | SNP | G | G | A | 235 | 5 | 0.02 | c.G178A#GAG->AAG#E->K# | p.E60K | MA2 |
| 12578071 | 12578071 | RNA | SNP | G | G | A | 60 | 15 | 0.2 | NA | NA | MA2 |
| 33625227 | 33625227 | RNA | SNP | C | C | T | 589 | 8 | 0.01 | NA | NA | MA2 |
| 35972046 | 35972046 | IGR | SNP | C | C | A | 187 | 7 | 0.03 | NA | NA | MA2 |
| 36060288 | 36060288 | Intron | SNP | A | A | T | 93 | 5 | 0.05 | NA | NA | MA2 |
| 36060288 | 36060288 | Intron | INS | - | - | T | 93 | 5 | 0.05 | NA | NA | MA2 |
| 98784296 | 98784296 | Intron | SNP | G | G | C | 56 | 50 | 0.47 | NA | NA | MA2 |
| 1.22E+08 | 1.22E+08 | Silent | SNP | C | C | T | 146 | 6 | 0.03 | c.G126A#GAG->GAA#E->E# | p.E42E | MA2 |
| 1.34E+08 | 1.34E+08 | Missense_Mutation | SNP | G | G | A | 46 | 6 | 0.11 | c.C257T#GCC->GTC#A->V# | p.A86V | MA2 |
| 17162250 | 17162250 | Intron | SNP | A | A | T | 147 | 14 | 0.08 | NA | NA | MA2 |
| 75102067 | 75102067 | Intron | SNP | C | C | T | 109 | 7 | 0.06 | NA | NA | MA2 |
| 88362755 | 88362755 | Intron | SNP | C | C | T | 56 | 48 | 0.46 | NA | NA | MA2 |
| 97366783 | 97366783 | Silent | SNP | C | C | T | 368 | 7 | 0.01 | c.G2874A#GAG->GAA#E->E# | p.E958E | MA2 |
| 1.04E+08 | 1.04E+08 | Intron | SNP | T | T | A | 123 | 4 | 0.03 | NA | NA | MA2 |
| 1.32E+08 | 1.32E+08 | Missense_Mutation | SNP | G | G | A | 258 | 7 | 0.02 | c.G287A#CGG->CAG#R->Q# | p.R96Q | MA2 |
| 533875 | 533875 | Missense_Mutation | SNP | G | G | T | 164 | 14 | 0.07 | c.C181A#CAG->AAG#Q->K# | p.Q61K | MA2 |
| 87071686 | 87071686 | Intron | SNP | T | T | G | 25 | 29 | 0.53 | NA | NA | MA2 |
| 94604054 | 94604054 | Silent | SNP | C | C | T | 52 | 54 | 0.5 | c.C1636T#CTG->TTG#L->L# | p.L546L | MA2 |
| 7322548 | 7322548 | Intron | SNP | C | C | A | 172 | 8 | 0.04 | NA | NA | MA2 |
| 8636755 | 8636755 | IGR | SNP | T | T | C | 95 | 4 | 0.04 | NA | NA | MA2 |
| 57244398 | 57244398 | Intron | SNP | T | T | C | 166 | 8 | 0.04 | NA | NA | MA2 |
| 75042219 | 75042219 | 3'UTR | SNP | A | A | T | 196 | 7 | 0.03 | NA | NA | MA2 |
| 1E+08 | 1E+08 | IGR | SNP | T | T | C | 72 | 4 | 0.05 | NA | NA | MA2 |
| 1.1E+08 | 1.1E+08 | 3'UTR | SNP | T | T | A | 173 | 3 | 0.01 | NA | NA | MA2 |
| 1.14E+08 | 1.14E+08 | Silent | SNP | C | C | G | 233 | 7 | 0.02 | c.G672C#TCG->TCC#S->S# | p.S224S | MA2 |
| 1.31E+08 | 1.31E+08 | Silent | SNP | G | G | A | 124 | 5 | 0.03 | c.G90A#GTG->GTA#V->V# | p.V30V | MA2 |
| 1.31E+08 | 1.31E+08 | 3'UTR | SNP | T | T | C | 85 | 9 | 0.09 | NA | NA | MA2 |
| 25097966 | 25097966 | Missense_Mutation | SNP | T | T | C | 179 | 10 | 0.05 | c.T1768C#TAT->CAT#Y->H# | p.Y590H | MA2 |
| 27945443 | 27945443 | IGR | SNP | G | G | A | 96 | 5 | 0.04 | NA | NA | MA2 |
| 38688560 | 38688560 | Missense_Mutation | SNP | C | C | T | 231 | 5 | 0.02 | c.C1216T#CGC->TGC#R->C# | p.R406C | MA2 |
| 98377906 | 98377906 | Missense_Mutation | SNP | C | C | G | 302 | 23 | 0.07 | c.C484G#CAC->GAC#H->D# | p.H162D | MA2 |
| 21924450 | 21924450 | IGR | SNP | C | C | A | 142 | 17 | 0.1 | NA | NA | MA2 |
| 22163321 | 22163321 | IGR | SNP | T | T | C | 162 | 15 | 0.08 | NA | NA | MA2 |
| 22314897 | 22314897 | IGR | SNP | G | G | T | 78 | 4 | 0.04 | NA | NA | MA2 |
| 35767076 | 35767076 | Intron | SNP | A | A | G | 18 | 16 | 0.47 | NA | NA | MA2 |
| 88841240 | 88841240 | Intron | SNP | T | T | A | 172 | 8 | 0.04 | NA | NA | MA2 |
| 90899306 | 90899306 | Intron | SNP | A | A | G | 168 | 13 | 0.07 | NA | NA | MA2 |
| 93613323 | 93613323 | Intron | SNP | T | T | A | 74 | 3 | 0.03 | NA | NA | MA2 |
| 93613324 | 93613325 | Intron | DEL | GA | GA | - | 74 | 3 | 0.03 | NA | NA | MA2 |
| 1.05E+08 | 1.05E+08 | Missense_Mutation | SNP | G | G | T | 320 | 4 | 0.01 | c.C7456A#CTA->ATA#L->I# | p.L2486I | MA2 |
| 1.07E+08 | 1.07E+08 | IGR | SNP | C | C | T | 381 | 50 | 0.11 | NA | NA | MA2 |
| 34495184 | 34495184 | IGR | SNP | C | C | T | 61 | 19 | 0.23 | NA | NA | MA2 |
| 34495681 | 34495682 | IGR | SNP | TA | TA | CG | 85 | 20 | 0.19 | NA | NA | MA2 |
| 34521717 | 34521717 | IGR | SNP | A | A | G | 41 | 19 | 0.31 | NA | NA | MA2 |
| 34943176 | 34943176 | Intron | SNP | C | C | T | 64 | 9 | 0.12 | NA | NA | MA2 |
| 40294453 | 40294453 | Intron | SNP | C | C | A | 147 | 4 | 0.02 | NA | NA | MA2 |
| 94855749 | 94855749 | RNA | SNP | C | C | T | 209 | 10 | 0.04 | NA | NA | MA2 |
| 98942868 | 98942868 | Intron | SNP | G | G | C | 303 | 7 | 0.02 | NA | NA | MA2 |
| 1362911 | 1362911 | Intron | SNP | G | G | C | 240 | 6 | 0.02 | NA | NA | MA2 |
| 1911833 | 1911833 | Silent | SNP | G | G | C | 166 | 3 | 0.01 | c.C786G#CGC->CGG#R->R# | p.R262R | MA2 |
| 26032691 | 26032691 | Intron | SNP | T | T | G | 54 | 13 | 0.19 | NA | NA | MA2 |
| 30761592 | 30761592 | Missense_Mutation | SNP | C | C | T | 161 | 5 | 0.03 | c.G157A#GCG->ACG#A->T# | p.A53T | MA2 |
| 74716105 | 74716105 | Intron | SNP | C | C | T | 31 | 29 | 0.48 | NA | NA | MA2 |
| 1109079 | 1109079 | 5'UTR | SNP | G | G | C | 115 | 4 | 0.03 | NA | NA | MA2 |
| 8493901 | 8493901 | Intron | SNP | A | A | T | 174 | 7 | 0.03 | NA | NA | MA2 |
| 21291768 | 21291768 | Intron | SNP | A | A | C | 49 | 15 | 0.23 | NA | NA | MA2 |
| 38920078 | 38920078 | 3'UTR | SNP | G | G | A | 62 | 4 | 0.06 | NA | NA | MA2 |
| 42169495 | 42169495 | Silent | SNP | G | G | A | 113 | 4 | 0.03 | c.C1572T#AGC->AGT#S->S# | p.S524S | MA2 |
| 43484205 | 43484205 | Intron | SNP | T | T | A | 389 | 7 | 0.01 | NA | NA | MA2 |
| 45586395 | 45586395 | IGR | SNP | G | G | T | 23 | 5 | 0.17 | NA | NA | MA2 |
| 78172138 | 78172138 | 3'UTR | SNP | C | C | T | 118 | 6 | 0.04 | NA | NA | MA2 |
| 81510981 | 81510981 | Silent | SNP | G | G | A | 138 | 6 | 0.04 | c.C930T#GCC->GCT#A->A# | p.A310A | MA2 |
| 81860384 | 81860384 | Silent | SNP | G | G | T | 72 | 6 | 0.07 | c.C88A#CGG->AGG#R->R# | p.R30R | MA2 |
| 7011960 | 7011960 | Intron | SNP | T | T | A | 45 | 26 | 0.36 | NA | NA | MA2 |
| 582775 | 582775 | 3'UTR | SNP | A | A | G | 51 | 8 | 0.13 | NA | NA | MA2 |
| 8488877 | 8488877 | 3'UTR | SNP | G | G | A | 115 | 3 | 0.02 | NA | NA | MA2 |
| 10149341 | 10149342 | Intron | SNP | AC | AC | CA | 116 | 9 | 0.07 | NA | NA | MA2 |
| 17511930 | 17511930 | Silent | SNP | T | T | C | 21 | 4 | 0.16 | c.T258C#GAT->GAC#D->D# | p.D86D | MA2 |
| 18177259 | 18177259 | Silent | SNP | A | A | G | 261 | 11 | 0.04 | c.A603G#CCA->CCG#P->P# | p.P201P | MA2 |
| 23827353 | 23827353 | RNA | SNP | T | T | A | 128 | 14 | 0.09 | NA | NA | MA2 |
| 32979971 | 32979971 | 3'UTR | SNP | G | G | C | 354 | 7 | 0.01 | NA | NA | MA2 |
| 35904967 | 35904967 | Intron | SNP | A | A | G | 224 | 11 | 0.04 | NA | NA | MA2 |
| 35904969 | 35904969 | Intron | SNP | A | A | G | 223 | 12 | 0.05 | NA | NA | MA2 |
| 35907552 | 35907552 | Silent | SNP | G | G | C | 131 | 5 | 0.03 | c.C90G#GGC->GGG#G->G# | p.G30G | MA2 |
| 38561391 | 38561391 | Silent | SNP | C | C | A | 99 | 19 | 0.16 | c.C12546A#CGC->CGA#R->R# | p.R4182R | MA2 |
| 5911576 | 5911576 | 5'UTR | SNP | T | T | C | 37 | 5 | 0.11 | NA | NA | MA2 |
| 5922577 | 5922577 | Missense_Mutation | SNP | G | G | A | 203 | 5 | 0.02 | c.G433A#GAC->AAC#D->N# | p.D145N | MA2 |
| 9337405 | 9337405 | Intron | SNP | G | G | A | 91 | 9 | 0.09 | NA | NA | MA2 |
| 29494941 | 29494941 | RNA | SNP | C | C | T | 90 | 16 | 0.15 | NA | NA | MA2 |
| 57700111 | 57700111 | Intron | SNP | G | G | A | 148 | 13 | 0.08 | NA | NA | MA2 |
| 10416141 | 10416141 | Intron | SNP | C | C | A | 26 | 4 | 0.13 | NA | NA | MA2 |
| 10427093 | 10427093 | Intron | SNP | G | G | C | 33 | 8 | 0.19 | NA | NA | MA2 |
| 10432017 | 10432017 | Intron | SNP | T | T | C | 26 | 4 | 0.13 | NA | NA | MA2 |
| 36486284 | 36486284 | Intron | SNP | G | G | A | 136 | 7 | 0.04 | NA | NA | MA2 |
| 44290951 | 44290951 | Intron | SNP | C | C | T | 193 | 16 | 0.07 | NA | NA | MA2 |
| 19210262 | 19210262 | Intron | SNP | G | G | C | 96 | 8 | 0.07 | NA | NA | MA2 |
| 3310612 | 3310612 | Missense_Mutation | SNP | G | G | C | 94 | 25 | 0.21 | c.C7591G#CTG->GTG#L->V# | p.L2531V | MA2 |
| 5991404 | 5991404 | Intron | SNP | C | C | A | 206 | 9 | 0.04 | NA | NA | MA2 |
| 36047047 | 36047047 | Missense_Mutation | SNP | G | G | C | 80 | 5 | 0.05 | c.G4201C#GAT->CAT#D->H# | p.D1401H | MA2 |
| 36047050 | 36047050 | Missense_Mutation | SNP | G | G | C | 78 | 4 | 0.04 | c.G4204C#GAC->CAC#D->H# | p.D1402H | MA2 |
| 41671415 | 41671415 | Intron | SNP | A | A | T | 168 | 7 | 0.04 | NA | NA | MA2 |
| 53254880 | 53254880 | Missense_Mutation | SNP | C | C | A | 130 | 5 | 0.03 | c.G1051T#GCC->TCC#A->S# | p.A351S | MA2 |
| 70237094 | 70237095 | nonframeshift substitution | SNP | TC | TC | GA | 377 | 9 | 0.02 | NA | NA | MA2 |
| 86085348 | 86085348 | IGR | SNP | A | A | G | 42 | 4 | 0.08 | NA | NA | MA2 |
| 1.07E+08 | 1.07E+08 | Missense_Mutation | SNP | G | G | A | 191 | 5 | 0.02 | c.G2147A#GGT->GAT#G->D# | p.G716D | MA2 |
| 1.16E+08 | 1.16E+08 | IGR | SNP | G | G | A | 155 | 9 | 0.05 | NA | NA | MA2 |
| 1.52E+08 | 1.52E+08 | Silent | SNP | G | G | A | 198 | 19 | 0.08 | c.G1434A#GGG->GGA#G->G# | p.G478G | MA2 |
| 56855353 | 56855353 | IGR | SNP | C | C | A | 37 | 7 | 0.15 | NA | NA | MA2 |
| 2835 | 2835 | RNA | SNP | C | C | T | 373 | 20 | 0.05 | NA | NA | MA2 |
| 3759 | 3759 | 3'Flank | SNP | A | A | G | 460 | 6 | 0.01 | NA | NA | MA2 |
| 6324 | 6324 | IGR | SNP | G | G | A | 338 | 191 | 0.36 | NA | NA | MA2 |
| 8701 | 8701 | IGR | SNP | A | A | G | 645 | 35 | 0.05 | NA | NA | MA2 |
| 9540 | 9540 | IGR | SNP | T | T | C | 402 | 20 | 0.04 | NA | NA | MA2 |
| 10873 | 10873 | IGR | SNP | T | T | C | 686 | 24 | 0.03 | NA | NA | MA2 |
| 15043 | 15043 | IGR | SNP | G | G | A | 424 | 9 | 0.02 | NA | NA | MA2 |
| 15301 | 15301 | IGR | SNP | G | G | A | 495 | 13 | 0.02 | NA | NA | MA2 |
| 15724 | 15724 | IGR | SNP | A | A | G | 566 | 8 | 0.01 | NA | NA | MA2 |
| 2172748 | 2172748 | Intron | SNP | G | G | A | 0 | 2 | 1 | NA | NA | MA7 |
| 2684196 | 2684196 | Intron | SNP | C | C | A | 7 | 4 | 0.36 | NA | NA | MA7 |
| 16605556 | 16605556 | Intron | SNP | T | T | A | 9 | 4 | 0.3 | NA | NA | MA7 |
| 16610460 | 16610460 | Intron | SNP | C | C | A | 10 | 3 | 0.23 | NA | NA | MA7 |
| 16705766 | 16705766 | RNA | SNP | C | C | T | 6 | 8 | 0.57 | NA | NA | MA7 |
| 26544709 | 26544709 | Intron | SNP | C | C | T | 3 | 6 | 0.66 | NA | NA | MA7 |
| 37752882 | 37752882 | Missense_Mutation | SNP | G | G | A | 6 | 2 | 0.25 | c.C1351T#CCC->TCC#P->S# | p.P451S | MA7 |
| 37872996 | 37872996 | Silent | SNP | G | G | A | 238 | 5 | 0.02 | c.C1629T#CCC->CCT#P->P# | p.P543P | MA7 |
| 42788226 | 42788226 | Intron | SNP | C | C | T | 2 | 2 | 0.5 | NA | NA | MA7 |
| 92108026 | 92108026 | Intron | SNP | G | G | C | 223 | 5 | 0.02 | NA | NA | MA7 |
| 1.52E+08 | 1.52E+08 | Intron | SNP | C | C | T | 58 | 7 | 0.1 | NA | NA | MA7 |
| 1.62E+08 | 1.62E+08 | Intron | SNP | A | A | T | 121 | 8 | 0.06 | NA | NA | MA7 |
| 2.03E+08 | 2.03E+08 | Intron | SNP | C | C | G | 5 | 7 | 0.58 | NA | NA | MA7 |
| 24704779 | 24704779 | Intron | SNP | A | A | G | 11 | 4 | 0.26 | NA | NA | MA7 |
| 49024728 | 49024728 | Intron | SNP | A | A | G | 4 | 6 | 0.6 | NA | NA | MA7 |
| 94872584 | 94872584 | RNA | SNP | A | A | T | 47 | 3 | 0.06 | NA | NA | MA7 |
| 1.65E+08 | 1.65E+08 | Intron | SNP | A | A | T | 27 | 3 | 0.1 | NA | NA | MA7 |
| 10396198 | 10396198 | Intron | SNP | C | C | T | 5 | 3 | 0.37 | NA | NA | MA7 |
| 1.25E+08 | 1.25E+08 | 3'UTR | SNP | G | G | A | 2 | 5 | 0.71 | NA | NA | MA7 |
| 3500240 | 3500240 | Intron | SNP | T | T | C | 113 | 4 | 0.03 | NA | NA | MA7 |
| 38021298 | 38021298 | Intron | SNP | C | C | A | 6 | 9 | 0.6 | NA | NA | MA7 |
| 71746334 | 71746334 | Intron | SNP | T | T | A | 187 | 5 | 0.02 | NA | NA | MA7 |
| 10249898 | 10249898 | 5'UTR | SNP | A | A | T | 42 | 7 | 0.14 | NA | NA | MA7 |
| 1.32E+08 | 1.32E+08 | Intron | SNP | G | G | T | 10 | 16 | 0.61 | NA | NA | MA7 |
| 1.74E+08 | 1.74E+08 | IGR | SNP | G | G | C | 10 | 9 | 0.47 | NA | NA | MA7 |
| 1.77E+08 | 1.77E+08 | Intron | SNP | T | T | G | 190 | 6 | 0.03 | NA | NA | MA7 |
| 1.77E+08 | 1.77E+08 | Intron | SNP | C | C | A | 191 | 6 | 0.03 | NA | NA | MA7 |
| 21197681 | 21197682 | Intron | SNP | AG | AG | CT | 48 | 3 | 0.05 | NA | NA | MA7 |
| 44261905 | 44261905 | Intron | INS | - | - | CATGCTGGCAGCTGGTGATTCTTGAGCCCAAGCATGTTCCCAGAGGTGGGAG | 15 | 4 | 0.21 | NA | NA | MA7 |
| 57101398 | 57101398 | Missense_Mutation | SNP | G | G | A | 390 | 4 | 0.01 | c.G982A#GTA->ATA#V->I# | p.V328I | MA7 |
| 57101403 | 57101403 | Silent | SNP | T | T | C | 396 | 4 | 0.01 | c.T987C#TAT->TAC#Y->Y# | p.Y329Y | MA7 |
| 1.38E+08 | 1.38E+08 | Silent | SNP | C | C | G | 94 | 3 | 0.03 | c.C4539G#CGC->CGG#R->R# | p.R1513R | MA7 |
| 1.61E+08 | 1.61E+08 | Intron | SNP | A | A | G | 18 | 4 | 0.18 | NA | NA | MA7 |
| 1.28E+08 | 1.28E+08 | 5'Flank | SNP | T | T | C | 142 | 6 | 0.04 | NA | NA | MA7 |
| 1.44E+08 | 1.44E+08 | 3'Flank | SNP | T | T | G | 244 | 7 | 0.02 | NA | NA | MA7 |
| 1.44E+08 | 1.44E+08 | 3'Flank | SNP | T | T | A | 243 | 7 | 0.02 | NA | NA | MA7 |
| 1.51E+08 | 1.51E+08 | Missense_Mutation | SNP | T | T | G | 205 | 4 | 0.01 | c.A215C#CAC->CCC#H->P# | p.H72P | MA7 |
| 1.51E+08 | 1.51E+08 | Silent | SNP | C | C | A | 210 | 4 | 0.01 | c.G210T#GGG->GGT#G->G# | p.G70G | MA7 |
| 1.52E+08 | 1.52E+08 | Intron | SNP | T | T | C | 21 | 4 | 0.16 | NA | NA | MA7 |
| 4147265 | 4147265 | Intron | SNP | T | T | C | 5 | 4 | 0.44 | NA | NA | MA7 |
| 12551523 | 12551523 | RNA | SNP | C | C | T | 28 | 7 | 0.2 | NA | NA | MA7 |
| 12551550 | 12551550 | RNA | SNP | T | T | C | 26 | 7 | 0.21 | NA | NA | MA7 |
| 12551758 | 12551758 | RNA | SNP | G | G | A | 7 | 3 | 0.3 | NA | NA | MA7 |
| 12554999 | 12554999 | RNA | SNP | G | G | C | 8 | 5 | 0.38 | NA | NA | MA7 |
| 1.2E+08 | 1.2E+08 | Intron | SNP | T | T | A | 203 | 7 | 0.03 | NA | NA | MA7 |
| 1.32E+08 | 1.32E+08 | Intron | SNP | C | C | G | 230 | 6 | 0.02 | NA | NA | MA7 |
| 1.4E+08 | 1.4E+08 | Intron | SNP | G | G | A | 4 | 2 | 0.33 | NA | NA | MA7 |
| 660315 | 660315 | Intron | SNP | C | C | G | 11 | 7 | 0.38 | NA | NA | MA7 |
| 42332963 | 42332963 | IGR | SNP | A | A | C | 8 | 4 | 0.33 | NA | NA | MA7 |
| 87845857 | 87845857 | RNA | SNP | A | A | T | 415 | 6 | 0.01 | NA | NA | MA7 |
| 90041288 | 90041288 | RNA | SNP | C | C | T | 2 | 2 | 0.5 | NA | NA | MA7 |
| 1737230 | 1737230 | 5'UTR | SNP | C | C | T | 60 | 4 | 0.06 | NA | NA | MA7 |
| 45527228 | 45527228 | Intron | SNP | T | T | C | 2 | 3 | 0.6 | NA | NA | MA7 |
| 73806502 | 73806502 | Intron | SNP | A | A | T | 201 | 10 | 0.04 | NA | NA | MA7 |
| 1.17E+08 | 1.17E+08 | Intron | SNP | C | C | G | 87 | 4 | 0.04 | NA | NA | MA7 |
| 78312310 | 78312310 | Intron | SNP | C | C | A | 7 | 9 | 0.56 | NA | NA | MA7 |
| 2677857 | 2677857 | Missense_Mutation | SNP | A | A | T | 136 | 4 | 0.02 | c.A5105T#GAC->GTC#D->V# | p.D1702V | MA7 |
| 18696096 | 18696096 | Intron | SNP | A | A | G | 118 | 5 | 0.04 | NA | NA | MA7 |
| 20752561 | 20752561 | 3'UTR | SNP | T | T | C | 241 | 9 | 0.03 | NA | NA | MA7 |
| 32486536 | 32486536 | Intron | SNP | C | C | A | 12 | 10 | 0.45 | NA | NA | MA7 |
| 64418721 | 64418721 | Intron | SNP | C | C | T | 107 | 3 | 0.02 | NA | NA | MA7 |
| 95966925 | 95966925 | Intron | SNP | T | T | C | 4 | 7 | 0.63 | NA | NA | MA7 |
| 1.32E+08 | 1.32E+08 | Intron | SNP | C | C | T | 12 | 5 | 0.29 | NA | NA | MA7 |
| 27793801 | 27793801 | Silent | SNP | C | C | G | 185 | 4 | 0.02 | c.C648G#GGC->GGG#G->G# | p.G216G | MA7 |
| 40758597 | 40758597 | Intron | SNP | C | C | A | 4 | 11 | 0.73 | NA | NA | MA7 |
| 19167999 | 19167999 | IGR | SNP | C | C | T | 6 | 3 | 0.33 | NA | NA | MA7 |
| 19374187 | 19374187 | IGR | SNP | C | C | T | 18 | 4 | 0.18 | NA | NA | MA7 |
| 19374243 | 19374243 | IGR | SNP | T | T | C | 19 | 6 | 0.24 | NA | NA | MA7 |
| 77406634 | 77406634 | Intron | SNP | C | C | G | 181 | 9 | 0.04 | NA | NA | MA7 |
| 28313474 | 28313474 | Intron | DEL | G | G | - | 7 | 3 | 0.3 | NA | NA | MA7 |
| 39011684 | 39011684 | IGR | SNP | C | C | T | 3 | 4 | 0.57 | NA | NA | MA7 |
| 90981824 | 90981824 | Missense_Mutation | SNP | A | A | G | 339 | 7 | 0.02 | c.T302C#ATT->ACT#I->T# | p.I101T | MA7 |
| 1.01E+08 | 1.01E+08 | Intron | SNP | T | T | C | 1 | 5 | 0.83 | NA | NA | MA7 |
| 1.01E+08 | 1.01E+08 | Intron | SNP | T | T | G | 1 | 5 | 0.83 | NA | NA | MA7 |
| 66932504 | 66932504 | Intron | SNP | T | T | C | 381 | 7 | 0.01 | NA | NA | MA7 |
| 66964818 | 66964819 | Intron | SNP | GG | GG | AA | 170 | 6 | 0.03 | NA | NA | MA7 |
| 67265250 | 67265250 | Intron | SNP | G | G | C | 47 | 3 | 0.06 | NA | NA | MA7 |
| 67831699 | 67831699 | Intron | SNP | G | G | C | 201 | 6 | 0.02 | NA | NA | MA7 |
| 9696803 | 9696803 | Intron | SNP | G | G | A | 2 | 8 | 0.8 | NA | NA | MA7 |
| 21293221 | 21293222 | Intron | SNP | TG | TG | CA | 3 | 3 | 0.5 | NA | NA | MA7 |
| 50976137 | 50976137 | Intron | SNP | A | A | T | 37 | 4 | 0.09 | NA | NA | MA7 |
| 78972895 | 78972895 | Intron | SNP | G | G | T | 103 | 5 | 0.04 | NA | NA | MA7 |
| 36805357 | 36805358 | Intron | SNP | CC | CC | GG | 125 | 3 | 0.02 | NA | NA | MA7 |
| 37440574 | 37440574 | Intron | SNP | G | G | A | 2 | 3 | 0.6 | NA | NA | MA7 |
| 45667566 | 45667566 | Intron | SNP | T | T | G | 7 | 13 | 0.65 | NA | NA | MA7 |
| 64179693 | 64179693 | RNA | SNP | C | C | T | 16 | 3 | 0.15 | NA | NA | MA7 |
| 2136704 | 2136704 | Intron | SNP | C | C | T | 5 | 6 | 0.54 | NA | NA | MA7 |
| 16921458 | 16921458 | Intron | SNP | T | T | A | 2 | 4 | 0.66 | NA | NA | MA7 |
| 35018521 | 35018521 | Intron | SNP | A | A | C | 2 | 3 | 0.6 | NA | NA | MA7 |
| 1476121 | 1476122 | 3'UTR | SNP | CT | CT | AG | 189 | 4 | 0.02 | NA | NA | MA7 |
| 29084016 | 29084016 | RNA | SNP | G | G | A | 20 | 5 | 0.2 | NA | NA | MA7 |
| 30384579 | 30384579 | RNA | SNP | A | A | T | 14 | 3 | 0.17 | NA | NA | MA7 |
| 30392018 | 30392018 | RNA | SNP | C | C | T | 6 | 4 | 0.4 | NA | NA | MA7 |
| 34568719 | 34568719 | Intron | SNP | G | G | C | 2 | 8 | 0.8 | NA | NA | MA7 |
| 5234059 | 5234059 | IGR | SNP | C | C | T | 16 | 4 | 0.2 | NA | NA | MA7 |
| 5239883 | 5239883 | IGR | SNP | A | A | T | 9 | 4 | 0.3 | NA | NA | MA7 |
| 9782429 | 9782429 | RNA | SNP | G | G | C | 5 | 4 | 0.44 | NA | NA | MA7 |
| 10427223 | 10427223 | Intron | SNP | A | A | G | 29 | 4 | 0.12 | NA | NA | MA7 |
| 10430112 | 10430112 | Intron | SNP | A | A | G | 15 | 4 | 0.21 | NA | NA | MA7 |
| 10430892 | 10430892 | Intron | SNP | T | T | C | 15 | 5 | 0.25 | NA | NA | MA7 |
| 10434250 | 10434251 | Intron | SNP | CC | CC | TT | 7 | 4 | 0.36 | NA | NA | MA7 |
| 10447767 | 10447767 | Intron | SNP | G | G | C | 6 | 3 | 0.33 | NA | NA | MA7 |
| 10448260 | 10448260 | Intron | SNP | A | A | G | 1 | 3 | 0.75 | NA | NA | MA7 |
| 10450122 | 10450122 | Intron | SNP | C | C | T | 11 | 3 | 0.21 | NA | NA | MA7 |
| 10463858 | 10463858 | Intron | SNP | G | G | A | 15 | 3 | 0.16 | NA | NA | MA7 |
| 10516986 | 10516986 | IGR | SNP | T | T | C | 4 | 4 | 0.5 | NA | NA | MA7 |
| 10568852 | 10568852 | Intron | SNP | A | A | T | 11 | 3 | 0.21 | NA | NA | MA7 |
| 16429324 | 16429324 | IGR | SNP | C | C | G | 23 | 5 | 0.17 | NA | NA | MA7 |
| 20717352 | 20717352 | Intron | SNP | G | G | C | 15 | 4 | 0.21 | NA | NA | MA7 |
| 32068599 | 32068599 | Missense_Mutation | SNP | C | C | T | 167 | 3 | 0.01 | c.C95T#TCG->TTG#S->L# | p.S32L | MA7 |
| 35336754 | 35336754 | RNA | SNP | T | T | C | 79 | 6 | 0.07 | NA | NA | MA7 |
| 41795393 | 41795393 | Intron | SNP | T | T | C | 188 | 6 | 0.03 | NA | NA | MA7 |
| 43829120 | 43829120 | Missense_Mutation | SNP | C | C | G | 86 | 5 | 0.05 | c.G682C#GAG->CAG#E->Q# | p.E228Q | MA7 |
| 50523791 | 50523791 | Silent | SNP | C | C | T | 256 | 6 | 0.02 | c.G621A#GTG->GTA#V->V# | p.V207V | MA7 |
| 16637195 | 16637195 | IGR | SNP | G | G | A | 36 | 10 | 0.21 | NA | NA | MA3 |
| 22661207 | 22661208 | nonframeshift substitution | SNP | GT | GT | AC | 260 | 10 | 0.03 | NA | NA | MA3 |
| 26546107 | 26546109 | Intron | SNP | ACA | ACA | TGT | 32 | 3 | 0.08 | NA | NA | MA3 |
| 28690205 | 28690205 | Intron | SNP | T | T | G | 87 | 17 | 0.16 | NA | NA | MA3 |
| 41159596 | 41159597 | Intron | SNP | TT | TT | AA | 186 | 5 | 0.02 | NA | NA | MA3 |
| 51402542 | 51402542 | Intron | SNP | A | A | T | 102 | 7 | 0.06 | NA | NA | MA3 |
| 1.14E+08 | 1.14E+08 | Intron | SNP | T | T | A | 54 | 9 | 0.14 | NA | NA | MA3 |
| 1.17E+08 | 1.17E+08 | IGR | SNP | T | T | G | 323 | 4 | 0.01 | NA | NA | MA3 |
| 1.46E+08 | 1.46E+08 | Silent | SNP | G | G | T | 250 | 7 | 0.02 | c.C502A#CGG->AGG#R->R# | p.R168R | MA3 |
| 2.25E+08 | 2.25E+08 | Intron | SNP | A | A | T | 101 | 8 | 0.07 | NA | NA | MA3 |
| 2.27E+08 | 2.27E+08 | IGR | SNP | T | T | C | 53 | 17 | 0.24 | NA | NA | MA3 |
| 2.28E+08 | 2.28E+08 | Missense_Mutation | SNP | T | T | C | 71 | 4 | 0.05 | c.T304C#TAT->CAT#Y->H# | p.Y102H | MA3 |
| 6865053 | 6865053 | Missense_Mutation | SNP | T | T | A | 148 | 5 | 0.03 | c.A644T#GAC->GTC#D->V# | p.D215V | MA3 |
| 15397791 | 15397791 | Intron | SNP | C | C | T | 41 | 16 | 0.28 | NA | NA | MA3 |
| 15397807 | 15397807 | Intron | SNP | G | G | C | 40 | 15 | 0.27 | NA | NA | MA3 |
| 26192225 | 26192225 | Intron | SNP | A | A | C | 74 | 7 | 0.08 | NA | NA | MA3 |
| 36773738 | 36773738 | Intron | SNP | T | T | A | 26 | 27 | 0.5 | NA | NA | MA3 |
| 36773772 | 36773772 | Intron | SNP | C | C | A | 35 | 42 | 0.54 | NA | NA | MA3 |
| 60782817 | 60782817 | Intron | SNP | A | A | T | 147 | 6 | 0.03 | NA | NA | MA3 |
| 1.58E+08 | 1.58E+08 | Intron | SNP | A | A | G | 158 | 17 | 0.09 | NA | NA | MA3 |
| 1.6E+08 | 1.6E+08 | nonframeshift substitution | SNP | AA | AA | TT | 215 | 7 | 0.03 | NA | NA | MA3 |
| 1.66E+08 | 1.66E+08 | RNA | SNP | A | A | T | 87 | 16 | 0.15 | NA | NA | MA3 |
| 31600545 | 31600545 | Intron | SNP | A | A | T | 36 | 40 | 0.52 | NA | NA | MA3 |
| 38277103 | 38277103 | Missense_Mutation | SNP | A | A | T | 72 | 3 | 0.04 | c.A1538T#GAC->GTC#D->V# | p.D513V | MA3 |
| 1.12E+08 | 1.12E+08 | Intron | SNP | T | T | G | 46 | 4 | 0.08 | NA | NA | MA3 |
| 87127164 | 87127164 | Intron | SNP | C | C | T | 110 | 16 | 0.12 | NA | NA | MA3 |
| 1.15E+08 | 1.15E+08 | Silent | SNP | G | G | A | 268 | 5 | 0.01 | c.C1905T#TTC->TTT#F->F# | p.F635F | MA3 |
| 1.28E+08 | 1.28E+08 | Intron | SNP | A | A | T | 71 | 5 | 0.06 | NA | NA | MA3 |
| 834017 | 834017 | Intron | SNP | G | G | A | 77 | 18 | 0.18 | NA | NA | MA3 |
| 40692053 | 40692053 | Missense_Mutation | SNP | T | T | G | 275 | 6 | 0.02 | c.T1142G#ATC->AGC#I->S# | p.I381S | MA3 |
| 1.36E+08 | 1.36E+08 | RNA | SNP | G | G | T | 45 | 4 | 0.08 | NA | NA | MA3 |
| 1.5E+08 | 1.5E+08 | Intron | SNP | G | G | A | 12 | 6 | 0.33 | NA | NA | MA3 |
| 1.77E+08 | 1.77E+08 | Intron | SNP | T | T | G | 131 | 4 | 0.02 | NA | NA | MA3 |
| 32580474 | 32580475 | Intron | SNP | CC | CC | TG | 34 | 7 | 0.17 | NA | NA | MA3 |
| 49517347 | 49517347 | Intron | SNP | C | C | T | 149 | 3 | 0.01 | NA | NA | MA3 |
| 2583749 | 2583749 | Intron | SNP | A | A | T | 17 | 3 | 0.15 | NA | NA | MA3 |
| 1.01E+08 | 1.01E+08 | Intron | SNP | T | T | C | 75 | 20 | 0.21 | NA | NA | MA3 |
| 1.29E+08 | 1.29E+08 | 3'UTR | SNP | T | T | G | 184 | 5 | 0.02 | NA | NA | MA3 |
| 28022020 | 28022020 | 3'UTR | SNP | C | C | T | 88 | 20 | 0.18 | NA | NA | MA3 |
| 34648784 | 34648785 | nonframeshift substitution | SNP | AG | AG | CT | 135 | 3 | 0.02 | NA | NA | MA3 |
| 35752035 | 35752035 | Missense_Mutation | SNP | T | T | A | 234 | 7 | 0.02 | c.T842A#GTG->GAG#V->E# | p.V281E | MA3 |
| 99004977 | 99004977 | Missense_Mutation | SNP | A | A | G | 108 | 4 | 0.03 | c.A1280G#GAG->GGG#E->G# | p.E427G | MA3 |
| 1.24E+08 | 1.24E+08 | Missense_Mutation | SNP | G | G | A | 118 | 4 | 0.03 | c.G280A#GTG->ATG#V->M# | p.V94M | MA3 |
| 1.3E+08 | 1.3E+08 | Intron | SNP | T | T | A | 175 | 4 | 0.02 | NA | NA | MA3 |
| 1.33E+08 | 1.33E+08 | Intron | SNP | A | A | T | 80 | 6 | 0.06 | NA | NA | MA3 |
| 32907206 | 32907207 | Intron | SNP | TG | TG | CA | 70 | 3 | 0.04 | NA | NA | MA3 |
| 71510915 | 71510915 | RNA | SNP | C | C | T | 134 | 6 | 0.04 | NA | NA | MA3 |
| 71810060 | 71810060 | Missense_Mutation | SNP | A | A | G | 126 | 5 | 0.03 | c.A2243G#AAT->AGT#N->S# | p.N748S | MA3 |
| 71810063 | 71810063 | Missense_Mutation | SNP | C | C | T | 126 | 5 | 0.03 | c.C2246T#ACT->ATT#T->I# | p.T749I | MA3 |
| 1.32E+08 | 1.32E+08 | Intron | SNP | G | G | A | 15 | 10 | 0.4 | NA | NA | MA3 |
| 64742112 | 64742113 | frameshift substitution | SNP | TC | TC | GA | 53 | 3 | 0.05 | NA | NA | MA3 |
| 68041270 | 68041270 | 5'UTR | SNP | C | C | A | 65 | 4 | 0.05 | NA | NA | MA3 |
| 1.13E+08 | 1.13E+08 | Intron | SNP | A | A | G | 51 | 36 | 0.41 | NA | NA | MA3 |
| 63145225 | 63145225 | 3'UTR | SNP | G | G | C | 59 | 4 | 0.06 | NA | NA | MA3 |
| 98665549 | 98665549 | Intron | SNP | A | A | T | 122 | 6 | 0.04 | NA | NA | MA3 |
| 20638356 | 20638356 | Missense_Mutation | SNP | A | A | T | 269 | 6 | 0.02 | c.A1354T#AGC->TGC#S->C# | p.S452C | MA3 |
| 32658197 | 32658197 | Intron | SNP | G | G | A | 55 | 49 | 0.47 | NA | NA | MA3 |
| 36329364 | 36329364 | Missense_Mutation | SNP | T | T | C | 142 | 4 | 0.02 | c.A1162G#AGG->GGG#R->G# | p.R388G | MA3 |
| 95590090 | 95590090 | Intron | SNP | T | T | A | 178 | 5 | 0.02 | NA | NA | MA3 |
| 20407973 | 20407973 | Missense_Mutation | SNP | C | C | G | 269 | 5 | 0.01 | c.G467C#AGT->ACT#S->T# | p.S156T | MA3 |
| 1.05E+08 | 1.05E+08 | Missense_Mutation | SNP | G | G | T | 103 | 3 | 0.02 | c.G4490T#CGG->CTG#R->L# | p.R1497L | MA3 |
| 1.05E+08 | 1.05E+08 | Missense_Mutation | SNP | A | A | C | 103 | 3 | 0.02 | c.A4495C#AGC->CGC#S->R# | p.S1499R | MA3 |
| 23364719 | 23364719 | RNA | SNP | A | A | G | 43 | 16 | 0.27 | NA | NA | MA3 |
| 55587981 | 55587981 | Intron | SNP | G | G | C | 49 | 4 | 0.07 | NA | NA | MA3 |
| 1362911 | 1362911 | Intron | SNP | G | G | C | 223 | 5 | 0.02 | NA | NA | MA3 |
| 3551984 | 3551984 | Intron | SNP | T | T | C | 49 | 9 | 0.15 | NA | NA | MA3 |
| 68337592 | 68337592 | Intron | SNP | T | T | A | 123 | 4 | 0.03 | NA | NA | MA3 |
| 5109624 | 5109624 | Missense_Mutation | SNP | T | T | A | 251 | 5 | 0.01 | c.A268T#ACT->TCT#T->S# | p.T90S | MA3 |
| 9905074 | 9905075 | nonframeshift substitution | SNP | AG | AG | CT | 189 | 4 | 0.02 | NA | NA | MA3 |
| 69415053 | 69415053 | Intron | SNP | T | T | C | 171 | 6 | 0.03 | NA | NA | MA3 |
| 69415056 | 69415056 | Intron | SNP | G | G | A | 170 | 7 | 0.03 | NA | NA | MA3 |
| 9996239 | 9996239 | Silent | SNP | A | A | G | 101 | 4 | 0.03 | c.T1446C#GGT->GGC#G->G# | p.G482G | MA3 |
| 10113914 | 10113914 | Missense_Mutation | SNP | G | G | A | 156 | 5 | 0.03 | c.G301A#GCG->ACG#A->T# | p.A101T | MA3 |
| 40744056 | 40744056 | Intron | SNP | C | C | G | 121 | 3 | 0.02 | NA | NA | MA3 |
| 55456466 | 55456466 | Missense_Mutation | SNP | G | G | C | 126 | 4 | 0.03 | c.C21G#AGC->AGG#S->R# | p.S7R | MA3 |
| 55456466 | 55456466 | Frame_Shift_Ins | INS | - | - | ATCCT | 126 | 4 | 0.03 | c.20_21insAGGAT | p.S7fs | MA3 |
| 1312762 | 1312762 | Missense_Mutation | SNP | C | C | T | 140 | 6 | 0.04 | c.G130A#GGG->AGG#G->R# | p.G44R | MA3 |
| 43667293 | 43667293 | Missense_Mutation | SNP | C | C | T | 2 | 2 | 0.5 | c.C10T#CGG->TGG#R->W# | p.R4W | MA3 |
| 10331040 | 10331040 | IGR | SNP | C | C | G | 85 | 16 | 0.15 | NA | NA | MA3 |
| 10331078 | 10331078 | IGR | SNP | G | G | C | 104 | 17 | 0.14 | NA | NA | MA3 |
| 10596238 | 10596238 | Intron | SNP | C | C | T | 55 | 12 | 0.17 | NA | NA | MA3 |
| 10601881 | 10601881 | Intron | SNP | G | G | A | 109 | 24 | 0.18 | NA | NA | MA3 |
| 32614272 | 32614272 | Intron | SNP | G | G | C | 105 | 4 | 0.03 | NA | NA | MA3 |
| 54550973 | 54550974 | nonframeshift substitution | SNP | TG | TG | CA | 171 | 6 | 0.03 | NA | NA | MA3 |
| 70237094 | 70237095 | nonframeshift substitution | SNP | TC | TC | GA | 394 | 10 | 0.02 | NA | NA | MA3 |
| 1.01E+08 | 1.01E+08 | Intron | SNP | C | C | G | 62 | 4 | 0.06 | NA | NA | MA3 |
| 12861721 | 12861721 | Missense_Mutation | SNP | C | C | T | 97 | 50 | 0.34 | c.C1367T#CCC->CTC#P->L# | p.P456L | M2 |
| 15922245 | 15922245 | Intron | SNP | A | A | T | 156 | 7 | 0.04 | NA | NA | M2 |
| 16604762 | 16604762 | Intron | SNP | C | C | T | 123 | 26 | 0.17 | NA | NA | M2 |
| 16630060 | 16630060 | RNA | SNP | C | C | T | 47 | 7 | 0.12 | NA | NA | M2 |
| 16646149 | 16646149 | RNA | SNP | C | C | T | 126 | 55 | 0.3 | NA | NA | M2 |
| 22661207 | 22661208 | nonframeshift substitution | SNP | GT | GT | AC | 394 | 7 | 0.01 | NA | NA | M2 |
| 22895600 | 22895600 | Intron | SNP | T | T | C | 246 | 9 | 0.03 | NA | NA | M2 |
| 22895607 | 22895607 | Intron | SNP | G | G | A | 234 | 8 | 0.03 | NA | NA | M2 |
| 28690206 | 28690206 | Intron | SNP | T | T | G | 97 | 16 | 0.14 | NA | NA | M2 |
| 41159596 | 41159597 | Intron | SNP | TT | TT | AA | 252 | 6 | 0.02 | NA | NA | M2 |
| 51402542 | 51402542 | Intron | SNP | A | A | T | 114 | 6 | 0.05 | NA | NA | M2 |
| 74572645 | 74572645 | Missense_Mutation | SNP | G | G | C | 617 | 7 | 0.01 | c.C3065G#GCC->GGC#A->G# | p.A1022G | M2 |
| 77955191 | 77955192 | Intron | SNP | CT | CT | AG | 99 | 5 | 0.04 | NA | NA | M2 |
| 94926811 | 94926811 | Intron | INS | - | - | CCCGGGGCA | 49 | 3 | 0.05 | NA | NA | M2 |
| 1E+08 | 1E+08 | Missense_Mutation | SNP | T | T | C | 364 | 18 | 0.04 | c.T995C#ATT->ACT#I->T# | p.I332T | M2 |
| 1.09E+08 | 1.09E+08 | 3'UTR | SNP | C | C | G | 195 | 4 | 0.02 | NA | NA | M2 |
| 1.51E+08 | 1.51E+08 | Intron | SNP | T | T | C | 231 | 7 | 0.02 | NA | NA | M2 |
| 1.51E+08 | 1.51E+08 | Intron | SNP | T | T | C | 234 | 7 | 0.02 | NA | NA | M2 |
| 1.57E+08 | 1.57E+08 | Missense_Mutation | SNP | T | T | C | 263 | 7 | 0.02 | c.T761C#CTC->CCC#L->P# | p.L254P | M2 |
| 1.57E+08 | 1.57E+08 | Missense_Mutation | SNP | G | G | C | 242 | 11 | 0.04 | c.G896C#AGT->ACT#S->T# | p.S299T | M2 |
| 1.83E+08 | 1.83E+08 | Intron | SNP | T | T | A | 224 | 5 | 0.02 | NA | NA | M2 |
| 2.27E+08 | 2.27E+08 | Missense_Mutation | SNP | G | G | C | 145 | 4 | 0.02 | c.C527G#TCC->TGC#S->C# | p.S176C | M2 |
| 2.47E+08 | 2.47E+08 | Missense_Mutation | SNP | G | G | C | 294 | 7 | 0.02 | c.C73G#CAG->GAG#Q->E# | p.Q25E | M2 |
| 1487753 | 1487753 | Intron | SNP | T | T | A | 169 | 6 | 0.03 | NA | NA | M2 |
| 1673678 | 1673678 | Missense_Mutation | SNP | G | G | A | 249 | 8 | 0.03 | c.C983T#ACG->ATG#T->M# | p.T328M | M2 |
| 23562561 | 23562562 | Intron | SNP | CT | CT | AG | 66 | 4 | 0.05 | NA | NA | M2 |
| 26440345 | 26440345 | Intron | SNP | G | G | A | 80 | 7 | 0.08 | NA | NA | M2 |
| 1.28E+08 | 1.28E+08 | Missense_Mutation | SNP | C | C | G | 688 | 10 | 0.01 | c.G821C#AGT->ACT#S->T# | p.S274T | M2 |
| 1.44E+08 | 1.44E+08 | Intron | SNP | CA | CA | TG | 95 | 6 | 0.05 | NA | NA | M2 |
| 1.48E+08 | 1.48E+08 | nonframeshift substitution | SNP | CC | CC | GG | 516 | 12 | 0.02 | NA | NA | M2 |
| 1.7E+08 | 1.7E+08 | IGR | SNP | C | C | T | 41 | 33 | 0.44 | NA | NA | M2 |
| 2.07E+08 | 2.07E+08 | Intron | INS | - | - | G | 91 | 13 | 0.12 | NA | NA | M2 |
| 30839317 | 30839317 | Intron | SNP | G | G | T | 98 | 7 | 0.06 | NA | NA | M2 |
| 30839320 | 30839320 | Intron | SNP | A | A | C | 97 | 7 | 0.06 | NA | NA | M2 |
| 38216092 | 38216092 | Intron | SNP | A | A | T | 255 | 8 | 0.03 | NA | NA | M2 |
| 38579547 | 38579547 | Intron | SNP | T | T | C | 65 | 3 | 0.04 | NA | NA | M2 |
| 49642838 | 49642838 | Missense_Mutation | SNP | T | T | C | 292 | 6 | 0.02 | c.T1204C#TCA->CCA#S->P# | p.S402P | M2 |
| 52576495 | 52576496 | Intron | SNP | TT | TT | AA | 125 | 5 | 0.03 | NA | NA | M2 |
| 1.29E+08 | 1.29E+08 | 3'UTR | SNP | G | G | C | 343 | 8 | 0.02 | NA | NA | M2 |
| 1.49E+08 | 1.49E+08 | Intron | SNP | T | T | G | 84 | 9 | 0.09 | NA | NA | M2 |
| 1.49E+08 | 1.49E+08 | Intron | SNP | T | T | G | 85 | 9 | 0.09 | NA | NA | M2 |
| 1.61E+08 | 1.61E+08 | Intron | SNP | A | A | T | 95 | 6 | 0.05 | NA | NA | M2 |
| 3246939 | 3246939 | Intron | SNP | A | A | T | 33 | 3 | 0.08 | NA | NA | M2 |
| 3889833 | 3889833 | IGR | SNP | C | C | T | 74 | 15 | 0.16 | NA | NA | M2 |
| 1.19E+08 | 1.19E+08 | RNA | SNP | C | C | T | 76 | 27 | 0.26 | NA | NA | M2 |
| 1.44E+08 | 1.44E+08 | RNA | SNP | T | T | C | 459 | 5 | 0.01 | NA | NA | M2 |
| 1.47E+08 | 1.47E+08 | 5'UTR | SNP | A | A | T | 127 | 5 | 0.03 | NA | NA | M2 |
| 1.65E+08 | 1.65E+08 | RNA | SNP | A | A | C | 148 | 13 | 0.08 | NA | NA | M2 |
| 1.77E+08 | 1.77E+08 | IGR | SNP | A | A | T | 7 | 8 | 0.53 | NA | NA | M2 |
| 16702920 | 16702920 | Intron | SNP | T | T | A | 239 | 7 | 0.02 | NA | NA | M2 |
| 40692053 | 40692053 | Missense_Mutation | SNP | T | T | G | 406 | 13 | 0.03 | c.T1142G#ATC->AGC#I->S# | p.I381S | M2 |
| 40692064 | 40692064 | Missense_Mutation | SNP | C | C | A | 395 | 9 | 0.02 | c.C1153A#CTG->ATG#L->M# | p.L385M | M2 |
| 55274638 | 55274639 | nonframeshift substitution | SNP | TT | TT | AA | 102 | 6 | 0.05 | NA | NA | M2 |
| 56929009 | 56929009 | Intron | SNP | T | T | A | 86 | 9 | 0.09 | NA | NA | M2 |
| 82058655 | 82058656 | Intron | SNP | AA | AA | TT | 151 | 4 | 0.02 | NA | NA | M2 |
| 1.16E+08 | 1.16E+08 | Intron | SNP | G | G | T | 143 | 14 | 0.08 | NA | NA | M2 |
| 1.5E+08 | 1.5E+08 | nonframeshift substitution | SNP | CC | CC | GG | 239 | 4 | 0.01 | NA | NA | M2 |
| 3850600 | 3850600 | Silent | SNP | G | G | C | 386 | 6 | 0.01 | c.G789C#GGG->GGC#G->G# | p.G263G | M2 |
| 17951438 | 17951438 | Intron | SNP | A | A | T | 90 | 9 | 0.09 | NA | NA | M2 |
| 26463267 | 26463268 | nonframeshift substitution | SNP | TC | TC | GA | 328 | 5 | 0.01 | NA | NA | M2 |
| 29801357 | 29801357 | IGR | SNP | C | C | G | 178 | 6 | 0.03 | NA | NA | M2 |
| 30626941 | 30626941 | Missense_Mutation | SNP | A | A | T | 286 | 5 | 0.01 | c.A56T#GAC->GTC#D->V# | p.D19V | M2 |
| 32097489 | 32097490 | Intron | SNP | TG | TG | CA | 181 | 7 | 0.03 | NA | NA | M2 |
| 36728652 | 36728653 | Intron | SNP | AG | AG | CT | 149 | 4 | 0.02 | NA | NA | M2 |
| 38677384 | 38677385 | Splice_Site | SNP | CT | CT | AG | 132 | 7 | 0.05 | NA | NA | M2 |
| 43139077 | 43139077 | Intron | SNP | A | A | T | 326 | 9 | 0.02 | NA | NA | M2 |
| 69796730 | 69796730 | Intron | SNP | G | G | C | 99 | 8 | 0.07 | NA | NA | M2 |
| 1.09E+08 | 1.09E+08 | Intron | SNP | T | T | A | 185 | 7 | 0.03 | NA | NA | M2 |
| 1.52E+08 | 1.52E+08 | Intron | SNP | C | C | T | 55 | 10 | 0.15 | NA | NA | M2 |
| 1.03E+08 | 1.03E+08 | Intron | SNP | G | G | A | 19 | 10 | 0.34 | NA | NA | M2 |
| 1.5E+08 | 1.5E+08 | Missense_Mutation | SNP | G | G | C | 95 | 4 | 0.04 | c.G532C#GCA->CCA#A->P# | p.A178P | M2 |
| 1.5E+08 | 1.5E+08 | IGR | SNP | A | A | G | 3 | 14 | 0.82 | NA | NA | M2 |
| 1780331 | 1780331 | Missense_Mutation | SNP | T | T | C | 239 | 6 | 0.02 | c.T625C#TAC->CAC#Y->H# | p.Y209H | M2 |
| 62248903 | 62248903 | 5'Flank | SNP | G | G | A | 8 | 8 | 0.5 | NA | NA | M2 |
| 35104550 | 35104550 | 3'UTR | SNP | T | T | A | 139 | 9 | 0.06 | NA | NA | M2 |
| 92277676 | 92277676 | Intron | SNP | A | A | T | 54 | 15 | 0.21 | NA | NA | M2 |
| 1.09E+08 | 1.09E+08 | Intron | SNP | A | A | T | 39 | 40 | 0.5 | NA | NA | M2 |
| 1.23E+08 | 1.23E+08 | Missense_Mutation | SNP | T | T | G | 386 | 10 | 0.02 | c.T558G#TGT->TGG#C->W# | p.C186W | M2 |
| 1.23E+08 | 1.23E+08 | Silent | SNP | C | C | A | 383 | 10 | 0.02 | c.C561A#GCC->GCA#A->A# | p.A187A | M2 |
| 6215041 | 6215041 | Intron | SNP | T | T | G | 63 | 7 | 0.1 | NA | NA | M2 |
| 73806502 | 73806502 | Intron | SNP | A | A | T | 242 | 5 | 0.02 | NA | NA | M2 |
| 1.19E+08 | 1.19E+08 | Missense_Mutation | SNP | T | T | G | 209 | 8 | 0.03 | c.T1342G#TTC->GTC#F->V# | p.F448V | M2 |
| 5634530 | 5634530 | Intron | SNP | C | C | T | 91 | 11 | 0.1 | NA | NA | M2 |
| 8730858 | 8730858 | Silent | SNP | T | T | A | 159 | 6 | 0.03 | c.A432T#GCA->GCT#A->A# | p.A144A | M2 |
| 9066866 | 9066867 | Intron | SNP | GG | GG | CC | 220 | 6 | 0.02 | NA | NA | M2 |
| 12518722 | 12518722 | Intron | SNP | A | A | G | 64 | 5 | 0.07 | NA | NA | M2 |
| 12518727 | 12518727 | Intron | SNP | C | C | G | 64 | 5 | 0.07 | NA | NA | M2 |
| 47477382 | 47477382 | Silent | SNP | A | A | T | 175 | 6 | 0.03 | c.T807A#GCT->GCA#A->A# | p.A269A | M2 |
| 64317193 | 64317193 | Intron | SNP | G | G | A | 296 | 11 | 0.03 | NA | NA | M2 |
| 65646002 | 65646002 | Intron | SNP | G | G | C | 241 | 4 | 0.01 | NA | NA | M2 |
| 66870650 | 66870651 | Intron | SNP | TC | TC | GA | 157 | 7 | 0.04 | NA | NA | M2 |
| 50120174 | 50120174 | Missense_Mutation | SNP | A | A | T | 161 | 6 | 0.03 | c.A131T#CAG->CTG#Q->L# | p.Q44L | M2 |
| 52317821 | 52317821 | Intron | INS | - | - | CTCTGAGGACCTGGCTGCCATGTGGCAGGTCCTCAGAGCC | 62 | 6 | 0.08 | NA | NA | M2 |
| 1.08E+08 | 1.08E+08 | Intron | SNP | T | T | C | 25 | 19 | 0.43 | NA | NA | M2 |
| 1.12E+08 | 1.12E+08 | Frame_Shift_Ins | INS | - | - | CTTCCTGGAGGAGGTGCACCACGACCGGCCCTGCATCCATGATGCTCTCCTGCTTATGGTGCACCTCCTCCAGGAAGTCCAGCACGCCCCCAGGGTCGCTGGG | 185 | 9 | 0.04 | c.1322_1323insCTTCCTGGAGGAGGTGCACCACGACCGGCCCTGCATCCATGATGCTCTCCTGCTTATGGTGCACCTCCTCCAGGAAGTCCAGCACGCCCCCAGGGTCGCTGGG | p.D441fs | M2 |
| 18178423 | 18178423 | IGR | SNP | A | A | G | 267 | 91 | 0.25 | NA | NA | M2 |
| 35162041 | 35162041 | Intron | SNP | A | A | T | 101 | 9 | 0.08 | NA | NA | M2 |
| 98885793 | 98885793 | Silent | SNP | G | G | A | 211 | 11 | 0.04 | c.C2178T#CAC->CAT#H->H# | p.H726H | M2 |
| 31145064 | 31145064 | Intron | SNP | G | G | T | 64 | 8 | 0.11 | NA | NA | M2 |
| 31145067 | 31145067 | Intron | SNP | A | A | C | 64 | 8 | 0.11 | NA | NA | M2 |
| 74555365 | 74555365 | Intron | SNP | G | G | A | 72 | 3 | 0.04 | NA | NA | M2 |
| 74555368 | 74555368 | Intron | SNP | T | T | C | 71 | 3 | 0.04 | NA | NA | M2 |
| 77236761 | 77236761 | Intron | SNP | T | T | A | 86 | 5 | 0.05 | NA | NA | M2 |
| 91481970 | 91481970 | Missense_Mutation | SNP | A | A | T | 285 | 11 | 0.03 | c.T521A#CTG->CAG#L->Q# | p.L174Q | M2 |
| 40037281 | 40037281 | Intron | SNP | A | A | G | 76 | 11 | 0.12 | NA | NA | M2 |
| 52248607 | 52248608 | Intron | DEL | CT | CT | - | 177 | 6 | 0.03 | NA | NA | M2 |
| 52248606 | 52248606 | Intron | SNP | A | A | T | 177 | 6 | 0.03 | NA | NA | M2 |
| 55587981 | 55587981 | Intron | SNP | G | G | C | 68 | 5 | 0.06 | NA | NA | M2 |
| 62256404 | 62256404 | IGR | SNP | C | C | T | 35 | 17 | 0.32 | NA | NA | M2 |
| 63747682 | 63747682 | Intron | SNP | G | G | A | 55 | 4 | 0.06 | NA | NA | M2 |
| 68350864 | 68350864 | Intron | SNP | A | A | T | 93 | 7 | 0.07 | NA | NA | M2 |
| 74175030 | 74175030 | Missense_Mutation | SNP | T | T | G | 375 | 10 | 0.02 | c.T172G#TCA->GCA#S->A# | p.S58A | M2 |
| 1.01E+08 | 1.01E+08 | Intron | SNP | C | C | G | 236 | 7 | 0.02 | NA | NA | M2 |
| 4890406 | 4890406 | Intron | SNP | A | A | T | 218 | 7 | 0.03 | NA | NA | M2 |
| 15130823 | 15130823 | RNA | SNP | T | T | C | 5 | 5 | 0.5 | NA | NA | M2 |
| 23433783 | 23433783 | Intron | SNP | A | A | T | 127 | 6 | 0.04 | NA | NA | M2 |
| 55811015 | 55811015 | Intron | SNP | A | A | C | 504 | 17 | 0.03 | NA | NA | M2 |
| 55816612 | 55816612 | Intron | SNP | T | T | G | 32 | 17 | 0.34 | NA | NA | M2 |
| 87412533 | 87412534 | nonframeshift substitution | SNP | GG | GG | CC | 233 | 9 | 0.03 | NA | NA | M2 |
| 11719194 | 11719195 | Intron | SNP | TG | TG | CA | 114 | 3 | 0.02 | NA | NA | M2 |
| 11719214 | 11719214 | Intron | SNP | A | A | G | 128 | 5 | 0.03 | NA | NA | M2 |
| 14236097 | 14236097 | Intron | SNP | C | C | G | 109 | 25 | 0.18 | NA | NA | M2 |
| 18422556 | 18422556 | RNA | SNP | C | C | A | 45 | 15 | 0.25 | NA | NA | M2 |
| 34579069 | 34579069 | Missense_Mutation | SNP | T | T | G | 311 | 11 | 0.03 | c.A212C#CAC->CCC#H->P# | p.H71P | M2 |
| 35820543 | 35820543 | Intron | SNP | A | A | T | 85 | 5 | 0.05 | NA | NA | M2 |
| 39405461 | 39405461 | 3'UTR | SNP | T | T | A | 79 | 7 | 0.08 | NA | NA | M2 |
| 41177849 | 41177850 | nonframeshift substitution | SNP | CA | CA | AG | 123 | 5 | 0.03 | NA | NA | M2 |
| 41884181 | 41884181 | Intron | SNP | A | A | T | 263 | 7 | 0.02 | NA | NA | M2 |
| 48961006 | 48961006 | Intron | SNP | T | T | A | 178 | 5 | 0.02 | NA | NA | M2 |
| 58206224 | 58206224 | Intron | SNP | C | C | G | 291 | 6 | 0.02 | NA | NA | M2 |
| 59151337 | 59151337 | Intron | SNP | T | T | G | 221 | 6 | 0.02 | NA | NA | M2 |
| 59151342 | 59151342 | Intron | SNP | C | C | A | 226 | 6 | 0.02 | NA | NA | M2 |
| 76101520 | 76101520 | Intron | SNP | G | G | C | 114 | 5 | 0.04 | NA | NA | M2 |
| 34829402 | 34829402 | Nonsense_Mutation | SNP | T | T | A | 270 | 5 | 0.01 | c.T134A#TTA->TAA#L->*# | p.L45X | M2 |
| 12464100 | 12464100 | Silent | SNP | G | G | T | 388 | 9 | 0.02 | c.C1822A#CGA->AGA#R->R# | p.R608R | M2 |
| 21537609 | 21537610 | nonframeshift substitution | SNP | TC | TC | AT | 149 | 8 | 0.05 | NA | NA | M2 |
| 35904967 | 35904967 | Intron | SNP | A | A | G | 261 | 9 | 0.03 | NA | NA | M2 |
| 42632853 | 42632853 | RNA | SNP | G | G | A | 111 | 7 | 0.05 | NA | NA | M2 |
| 49537106 | 49537106 | Silent | SNP | G | G | A | 226 | 7 | 0.03 | c.G519A#CGG->CGA#R->R# | p.R173R | M2 |
| 54250956 | 54250956 | Missense_Mutation | SNP | G | G | T | 39 | 21 | 0.35 | c.C1816A#CCC->ACC#P->T# | p.P606T | M2 |
| 55759189 | 55759190 | Intron | SNP | TA | TA | CT | 126 | 17 | 0.11 | NA | NA | M2 |
| 409934 | 409934 | Missense_Mutation | SNP | G | G | A | 250 | 7 | 0.02 | c.G76A#GTG->ATG#V->M# | p.V26M | M2 |
| 28581884 | 28581884 | RNA | SNP | C | C | T | 84 | 6 | 0.06 | NA | NA | M2 |
| 31476384 | 31476384 | 5'UTR | SNP | A | A | T | 236 | 7 | 0.02 | NA | NA | M2 |
| 41203828 | 41203828 | Nonsense_Mutation | SNP | C | C | T | 506 | 5 | 0 | c.G1089A#TGG->TGA#W->*# | p.W363X | M2 |
| 41203835 | 41203835 | Missense_Mutation | SNP | A | A | G | 501 | 5 | 0 | c.T1082C#ATC->ACC#I->T# | p.I361T | M2 |
| 10330538 | 10330538 | IGR | SNP | C | C | T | 71 | 14 | 0.16 | NA | NA | M2 |
| 10330762 | 10330762 | IGR | SNP | T | T | A | 116 | 17 | 0.12 | NA | NA | M2 |
| 10452560 | 10452560 | Intron | SNP | T | T | C | 45 | 12 | 0.21 | NA | NA | M2 |
| 14009586 | 14009586 | IGR | SNP | T | T | C | 18 | 23 | 0.56 | NA | NA | M2 |
| 16187945 | 16187945 | RNA | SNP | A | A | T | 412 | 9 | 0.02 | NA | NA | M2 |
| 42859885 | 42859885 | Intron | SNP | T | T | A | 74 | 4 | 0.05 | NA | NA | M2 |
| 45515769 | 45515769 | Silent | SNP | G | G | C | 256 | 5 | 0.01 | c.C1545G#GGC->GGG#G->G# | p.G515G | M2 |
| 23998812 | 23998812 | RNA | SNP | A | A | T | 114 | 20 | 0.14 | NA | NA | M2 |
| 37767969 | 37767969 | RNA | SNP | G | G | C | 137 | 6 | 0.04 | NA | NA | M2 |
| 43220580 | 43220580 | Silent | SNP | G | G | A | 214 | 9 | 0.04 | c.C1557T#TGC->TGT#C->C# | p.C519C | M2 |
| 46279993 | 46279993 | Intron | INS | - | - | GGCATGGGCTTGCAT | 149 | 4 | 0.02 | NA | NA | M2 |
| 65531314 | 65531315 | Intron | SNP | CC | CC | GG | 156 | 4 | 0.02 | NA | NA | M2 |
| 95063172 | 95063172 | RNA | SNP | C | C | T | 62 | 7 | 0.1 | NA | NA | M2 |

Table S3. Clinical characteristics of patients for tissue microarray

| Point Site | Gender | Age | WHO+X | WHO+Y |
| --- | --- | --- | --- | --- |
| A01 | Female | 63 | A | A |
| A02 | Male | 63 | A | A |
| A03 | Female | 63 | A | A |
| A04 | Female | 49 | A | AB |
| A05 | Female | 57 | AB | B |
| B01 | Female | 54 | AB | B |
| B02 | Female | 48 | AB | AB |
| B03 | Female | 68 | AB | AB |
| B04 | Female | 23 | B | B |
| B05 | Male | 45 | B | B |
| C01 | Male | 58 | B | B |
| C02 | Male | 48 | B | B |
| C03 | Male | 50 | B | AB |
| C04 | Female | 44 | B | AB |
| C05 | Female | 52 | B | B |
| D01 | Female | 42 | B | B |
| D02 | Female | 51 | B | AB |
| D03 | Male | 47 | B | B |
| D04 | Female | 62 | B | AB |
| D05 | Male | 61 | B | A |
| E01 | Female | 57 | C | C |
| E02 | Female | 53 | C | C |
| E03 | Male | 54 | C | C |
| E04 | Female | 60 | C | C |
| E05 | Male | 59 | C | C |

Table S4.Samples in TCGA dataset included in subsequent analyses

| sample | OS | OS.time | DSS | DSS.time | PFI | PFI.time | WHO Type | KRT14 Expression | Subtype |
| --- | --- | --- | --- | --- | --- | --- | --- | --- | --- |
| TCGA.3G.AB0O.01A | 0 | 2086 | 0 | 2086 | 0 | 2086 | B1 | low | T2 |
| TCGA.3G.AB0Q.01A | 0 | 1799 | 0 | 1799 | 0 | 1799 | A | high | T1 |
| TCGA.3G.AB0T.01A | 1 | 2488 | 1 | 2488 | 1 | 1786 | B1 | low | T2 |
| TCGA.3G.AB14.01A | 1 | 775 | 0 | 775 | 0 | 775 | B2 | low | T2 |
| TCGA.3G.AB19.01A | 1 | 2910 | 0 | 2910 | 1 | 2420 | A | high | T1 |
| TCGA.3Q.A9WF.01A | 0 | 467 | 0 | 467 | 0 | 467 | B2 | high | T1 |
| TCGA.3S.A8YW.01A | 0 | 643 | 0 | 643 | 1 | 224 | C | -- | T3 |
| TCGA.3S.AAYX.01A | 0 | 571 | 0 | 571 | 0 | 571 | B1 | high | T1 |
| TCGA.3T.AA9L.01A | 0 | 1050 | 0 | 1050 | 1 | 702 | B1 | high | T1 |
| TCGA.4V.A9QI.01A | 0 | 2936 | 0 | 2936 | 0 | 2936 | B2 | high | T1 |
| TCGA.4V.A9QJ.01A | 0 | 2849 | 0 | 2849 | 1 | 793 | B3 | low | T2 |
| TCGA.4V.A9QL.01A | 0 | 1908 | 0 | 1908 | 0 | 1908 | AB | high | T1 |
| TCGA.4V.A9QM.01A | 0 | 1253 | 0 | 1253 | 0 | 1253 | AB | high | T1 |
| TCGA.4V.A9QN.01A | 0 | 970 | 0 | 970 | 1 | 937 | B3 | high | T1 |
| TCGA.4V.A9QR.01A | 0 | 780 | 0 | 780 | 0 | 780 | B3 | low | T2 |
| TCGA.4V.A9QS.01A | 0 | 774 | 0 | 774 | 1 | 752 | B2 | high | T1 |
| TCGA.4V.A9QT.01A | 0 | 813 | 0 | 813 | 0 | 813 | B1 | low | T2 |
| TCGA.4V.A9QU.01A | 0 | 627 | 0 | 627 | 0 | 627 | AB | high | T1 |
| TCGA.4V.A9QW.01A | 0 | 630 | 0 | 630 | 0 | 630 | AB | high | T1 |
| TCGA.4V.A9QX.01A | 0 | 434 | 0 | 434 | 0 | 434 | AB | high | T1 |
| TCGA.4X.A9F9.01A | 0 | 669 | 0 | 669 | 0 | 669 | B1 | high | T1 |
| TCGA.4X.A9FA.01A | 0 | 392 | 0 | 392 | 0 | 392 | A | high | T1 |
| TCGA.4X.A9FB.01A | 0 | 717 | 0 | 717 | 0 | 717 | B2 | high | T1 |
| TCGA.4X.A9FC.01A | 0 | 2072 | 0 | 2072 | 0 | 2072 | AB | high | T1 |
| TCGA.4X.A9FD.01A | 0 | 1503 | 0 | 1503 | 0 | 1503 | B2 | low | T2 |
| TCGA.5G.A9ZZ.01A | 0 | 835 | 0 | 835 | 0 | 835 | C | -- | T3 |
| TCGA.5K.AAAP.01A | 0 | 1054 | 0 | 1054 | 0 | 1054 | A | high | T1 |
| TCGA.5U.AB0D.01A | 0 | 714 | 0 | 714 | 0 | 714 | C | -- | T3 |
| TCGA.5U.AB0E.01A | 0 | 687 | 0 | 687 | 0 | 687 | B3 | high | T1 |
| TCGA.5U.AB0F.01A | 0 | 459 | 0 | 459 | 0 | 459 | AB | high | T1 |
| TCGA.5V.A9RR.01A | 0 | 827 | 0 | 827 | 0 | 827 | AB | high | T1 |
| TCGA.X7.A8D6.01A | 0 | 4225 | 0 | 4225 | 1 | 2928 | B2 | high | T1 |
| TCGA.X7.A8D6.11A | 0 | 4225 | 0 | 4225 | 1 | 2928 | B2 | high | T1 |
| TCGA.X7.A8D7.01A | 0 | 325 | 0 | 325 | 1 | 265 | B1 | high | T1 |
| TCGA.X7.A8D7.11A | 0 | 325 | 0 | 325 | 1 | 265 | B1 | high | T1 |
| TCGA.X7.A8D8.01A | 0 | 58 | 0 | 58 | 0 | 58 | AB | high | T1 |
| TCGA.X7.A8D9.01A | 0 | NA | 0 | NA | 0 | NA | AB | high | T1 |
| TCGA.X7.A8DB.01A | 0 | 1992 | 0 | 1992 | 0 | 1992 | B2 | high | T1 |
| TCGA.X7.A8DD.01A | 0 | 1114 | 0 | 1114 | 0 | 1114 | B1 | high | T1 |
| TCGA.X7.A8DE.01A | 0 | 981 | 0 | 981 | 0 | 981 | B2 | high | T1 |
| TCGA.X7.A8DF.01A | 0 | 901 | 0 | 901 | 0 | 901 | AB | high | T1 |
| TCGA.X7.A8DG.01A | 0 | 695 | 0 | 695 | 0 | 695 | B2 | high | T1 |
| TCGA.X7.A8DJ.01A | 0 | 289 | 0 | 289 | 0 | 289 | AB | high | T1 |
| TCGA.X7.A8M0.01A | 0 | 441 | 0 | 441 | 0 | 441 | B3 | high | T1 |
| TCGA.X7.A8M1.01A | 0 | 1282 | 0 | 1282 | 0 | 1282 | AB | high | T1 |
| TCGA.X7.A8M3.01A | 0 | 50 | 0 | 50 | 0 | 50 | B3 | low | T2 |
| TCGA.X7.A8M4.01A | 0 | 3808 | 0 | 3808 | 0 | 3808 | B2 | high | T1 |
| TCGA.X7.A8M5.01A | 0 | 1645 | 0 | 1645 | 0 | 1645 | B3 | high | T1 |
| TCGA.X7.A8M6.01A | 0 | 4575 | 0 | 4575 | 1 | 1901 | B2 | high | T1 |
| TCGA.X7.A8M7.01A | 0 | 1972 | 0 | 1972 | 0 | 1972 | B2 | low | T2 |
| TCGA.X7.A8M8.01A | 0 | 2533 | 0 | 2533 | 0 | 2533 | B2 | high | T1 |
| TCGA.XH.A853.01A | 0 | 1220 | 0 | 1220 | 1 | 196 | AB | high | T1 |
| TCGA.XM.A8R8.01A | 0 | 1598 | 0 | 1598 | 1 | 244 | C | -- | T3 |
| TCGA.XM.A8R9.01A | 0 | 1488 | 0 | 1488 | 0 | 1488 | A | high | T1 |
| TCGA.XM.A8RB.01A | 0 | 1144 | 0 | 1144 | 0 | 1144 | B1 | low | T2 |
| TCGA.XM.A8RC.01A | 0 | 2963 | 0 | 2963 | 0 | 2963 | B2 | high | T1 |
| TCGA.XM.A8RD.01A | 0 | 1546 | 0 | 1546 | 0 | 1546 | B2 | high | T1 |
| TCGA.XM.A8RE.01A | 0 | 422 | 0 | 422 | 0 | 422 | AB | high | T1 |
| TCGA.XM.A8RF.01A | 0 | 797 | 0 | 797 | 0 | 797 | A | high | T1 |
| TCGA.XM.A8RG.01A | 0 | 386 | 0 | 386 | 0 | 386 | A | high | T1 |
| TCGA.XM.A8RH.01A | 0 | 363 | 0 | 363 | 0 | 363 | B1 | high | T1 |
| TCGA.XM.A8RI.01A | 0 | 387 | 0 | 387 | 0 | 387 | B2 | high | T1 |
| TCGA.XM.A8RL.01A | 0 | 2562 | 0 | 2562 | 0 | 2562 | B1 | low | T2 |
| TCGA.XM.AAZ1.01A | 0 | 397 | 0 | 397 | 0 | 397 | B3 | low | T2 |
| TCGA.XM.AAZ2.01A | 0 | 981 | 0 | 981 | 0 | 981 | A | high | T1 |
| TCGA.XM.AAZ3.01A | 0 | 1150 | 0 | 1150 | 0 | 1150 | AB | high | T1 |
| TCGA.XU.A92O.01A | 0 | 3410 | 0 | 3410 | 0 | 3410 | A | high | T1 |
| TCGA.XU.A92Q.01A | 0 | 1092 | 0 | 1092 | 0 | 1092 | A | high | T1 |
| TCGA.XU.A92T.01A | 0 | 1376 | 0 | 1376 | 0 | 1376 | AB | high | T1 |
| TCGA.XU.A92U.01A | 0 | 4562 | 0 | 4562 | 0 | 4562 | B2 | high | T1 |
| TCGA.XU.A92V.01A | 1 | 124 | 0 | 124 | 0 | 124 | AB | low | T2 |
| TCGA.XU.A92W.01A | 0 | 3279 | 0 | 3279 | 0 | 3279 | AB | low | T2 |
| TCGA.XU.A92X.01A | 0 | 2343 | 0 | 2343 | 0 | 2343 | B2 | high | T1 |
| TCGA.XU.A92Y.01A | 1 | 3488 | 1 | 3488 | 1 | 1224 | B3 | low | T2 |
| TCGA.XU.A92Z.01A | 0 | 3663 | 0 | 3663 | 0 | 3663 | C | -- | T3 |
| TCGA.XU.A930.01A | 0 | 2514 | 0 | 2514 | 0 | 2514 | B3 | low | T2 |
| TCGA.XU.A931.01A | 1 | 1609 | 0 | 1609 | 1 | 1377 | B3 | low | T2 |
| TCGA.XU.A932.01A | 0 | 2149 | 0 | 2149 | 0 | 2149 | B2 | low | T2 |
| TCGA.XU.A933.01A | 0 | 1789 | 0 | 1789 | 0 | 1789 | C | -- | T3 |
| TCGA.XU.A936.01A | 0 | 643 | 0 | 643 | 0 | 643 | C | -- | T3 |
| TCGA.XU.AAXV.01A | 0 | 4070 | 0 | 4070 | 0 | 4070 | B2 | low | T2 |
| TCGA.XU.AAXW.01A | 0 | 3762 | 0 | 3762 | 0 | 3762 | B2 | high | T1 |
| TCGA.XU.AAXX.01A | 0 | 2721 | 0 | 2721 | 0 | 2721 | B2 | low | T2 |
| TCGA.XU.AAXY.01A | 0 | 1891 | 0 | 1891 | 0 | 1891 | B2 | high | T1 |
| TCGA.XU.AAXZ.01A | 0 | 1537 | 0 | 1537 | 0 | 1537 | B3 | high | T1 |
| TCGA.XU.AAY0.01A | 0 | 1502 | 0 | 1502 | 0 | 1502 | AB | high | T1 |
| TCGA.XU.AAY1.01A | 0 | 3268 | 0 | 3268 | 0 | 3268 | AB | high | T1 |
| TCGA.YT.A95D.01A | 0 | 750 | 0 | 750 | 1 | 168 | AB | high | T1 |
| TCGA.YT.A95E.01A | 0 | 1070 | 0 | 1070 | 0 | 1070 | B2 | high | T1 |
| TCGA.YT.A95F.01A | 0 | 775 | 0 | 775 | 0 | 775 | AB | high | T1 |
| TCGA.YT.A95G.01A | 0 | 1514 | 0 | 1514 | 0 | 1514 | B2 | low | T2 |
| TCGA.YT.A95H.01A | 0 | 711 | 0 | 711 | 0 | 711 | AB | high | T1 |
| TCGA.ZB.A961.01A | 1 | 180 | 0 | 180 | 0 | 180 | A | low | T2 |
| TCGA.ZB.A963.01A | 0 | 1482 | 0 | 1482 | 0 | 1482 | AB | high | T1 |
| TCGA.ZB.A964.01A | 0 | 1287 | 0 | 1287 | 0 | 1287 | A | high | T1 |
| TCGA.ZB.A965.01A | 0 | 816 | 0 | 816 | 0 | 816 | AB | high | T1 |
| TCGA.ZB.A966.01A | 1 | 379 | 1 | 379 | 1 | 296 | C | -- | T3 |
| TCGA.ZB.A969.01A | 0 | 1046 | 0 | 1046 | 0 | 1046 | C | -- | T3 |
| TCGA.ZB.A96A.01A | 0 | 2354 | 0 | 2354 | 0 | 2354 | B3 | low | T2 |
| TCGA.ZB.A96B.01A | 0 | 2211 | 0 | 2211 | 1 | 91 | B1 | high | T1 |
| TCGA.ZB.A96C.01A | 0 | 2163 | 0 | 2163 | 0 | 2163 | AB | high | T1 |
| TCGA.ZB.A96D.01A | 0 | 1960 | 0 | 1960 | 0 | 1960 | AB | high | T1 |
| TCGA.ZB.A96E.01A | 0 | 1819 | 0 | 1819 | 0 | 1819 | AB | high | T1 |
| TCGA.ZB.A96F.01A | 0 | 1761 | 0 | 1761 | 0 | 1761 | AB | low | T2 |
| TCGA.ZB.A96G.01A | 0 | 1606 | 0 | 1606 | 0 | 1606 | AB | high | T1 |
| TCGA.ZB.A96H.01A | 0 | 1504 | 0 | 1504 | 0 | 1504 | A | high | T1 |
| TCGA.ZB.A96I.01A | 0 | 1653 | 0 | 1653 | 0 | 1653 | AB | high | T1 |
| TCGA.ZB.A96K.01A | 0 | 1388 | 0 | 1388 | 0 | 1388 | AB | high | T1 |
| TCGA.ZB.A96L.01A | 0 | 1529 | 0 | 1529 | 0 | 1529 | B3 | high | T1 |
| TCGA.ZB.A96M.01A | 0 | 14 | 0 | 14 | 0 | 14 | AB | high | T1 |
| TCGA.ZB.A96O.01A | 0 | 1050 | 0 | 1050 | 0 | 1050 | B1 | low | T2 |
| TCGA.ZB.A96P.01A | 0 | 753 | 0 | 753 | 0 | 753 | B1 | low | T2 |
| TCGA.ZB.A96Q.01A | 0 | 675 | 0 | 675 | 0 | 675 | AB | high | T1 |
| TCGA.ZB.A96R.01A | 0 | 1804 | 0 | 1804 | 0 | 1804 | B2 | high | T1 |
| TCGA.ZB.A96V.01A | 0 | 932 | 0 | 932 | 0 | 932 | C | -- | T3 |
| TCGA.ZC.AAA7.01A | 1 | 853 | 1 | 853 | 1 | 853 | C | -- | T3 |
| TCGA.ZC.AAAA.01A | 0 | 819 | 0 | 819 | 1 | 137 | B2 | low | T2 |
| TCGA.ZC.AAAF.01A | 0 | 1165 | 0 | 1165 | 0 | 1165 | AB | high | T1 |
| TCGA.ZC.AAAH.01A | 0 | 497 | 0 | 497 | 1 | 166 | B2 | high | T1 |
| TCGA.ZL.A9V6.01A | 0 | 2096 | 0 | 2096 | 0 | 2096 | AB | high | T1 |
| TCGA.ZT.A8OM.01A | 0 | 1398 | 0 | 1398 | 0 | 1398 | A | high | T1 |

Table S5. List of genes differentially-expressed in T1, T2 and T3 subtypes in TCGA.

| Genes | logFC | AveExpr | P.Value | adj.P.Val | cluster | label |
| --- | --- | --- | --- | --- | --- | --- |
| KRT14 | 5.103205 | 5.010682 | 2.45E-20 | 1.43E-15 | T1 | P-val <= 0.05 |
| SCN4B | 2.465787 | 3.856048 | 1.49E-11 | 7.82E-08 | T1 | P-val <= 0.05 |
| KLK8 | 2.518258 | 2.265943 | 2.00E-11 | 8.11E-08 | T1 | P-val <= 0.05 |
| MED15P9 | 1.91962 | 1.652141 | 2.08E-11 | 8.11E-08 | T1 | P-val <= 0.05 |
| MYL9 | 2.282432 | 5.47601 | 2.57E-11 | 9.32E-08 | T1 | P-val <= 0.05 |
| FAR2P1 | 2.330641 | 2.14176 | 4.93E-11 | 1.37E-07 | T1 | P-val <= 0.05 |
| DLK2 | 1.828637 | 2.481418 | 1.13E-10 | 2.27E-07 | T1 | P-val <= 0.05 |
| GJB6 | 2.391519 | 3.594752 | 1.65E-10 | 2.91E-07 | T1 | P-val <= 0.05 |
| C16orf89 | 2.281242 | 2.671849 | 4.18E-10 | 6.26E-07 | T1 | P-val <= 0.05 |
| CRYAB | 2.569877 | 3.746908 | 5.61E-10 | 7.28E-07 | T1 | P-val <= 0.05 |
| STOX1 | 1.706712 | 3.074336 | 6.31E-10 | 7.29E-07 | T1 | P-val <= 0.05 |
| PHEX | 2.471595 | 2.260287 | 1.24E-09 | 1.14E-06 | T1 | P-val <= 0.05 |
| OR7E122P | 2.204894 | 2.026672 | 1.30E-09 | 1.17E-06 | T1 | P-val <= 0.05 |
| TBX1 | 2.791075 | 2.647209 | 1.40E-09 | 1.17E-06 | T1 | P-val <= 0.05 |
| BCAM | 2.349473 | 5.040556 | 2.14E-09 | 1.60E-06 | T1 | P-val <= 0.05 |
| PDGFRL | 2.508853 | 3.275926 | 2.76E-09 | 1.94E-06 | T1 | P-val <= 0.05 |
| SGCA | 1.889489 | 1.85829 | 2.99E-09 | 2.06E-06 | T1 | P-val <= 0.05 |
| SMIM1 | 2.121167 | 3.041188 | 3.83E-09 | 2.36E-06 | T1 | P-val <= 0.05 |
| WNT5B | 1.747313 | 3.010663 | 3.90E-09 | 2.37E-06 | T1 | P-val <= 0.05 |
| FAR2P4 | 1.568921 | 1.330067 | 5.37E-09 | 3.01E-06 | T1 | P-val <= 0.05 |
| FAM132A | 2.128554 | 2.359279 | 5.85E-09 | 3.19E-06 | T1 | P-val <= 0.05 |
| CCDC144NL-AS1 | 1.829238 | 1.580696 | 8.09E-09 | 3.95E-06 | T1 | P-val <= 0.05 |
| CCDC3 | 2.034582 | 5.589824 | 8.29E-09 | 4.00E-06 | T1 | P-val <= 0.05 |
| GGT5 | 1.586582 | 3.355936 | 1.45E-08 | 5.80E-06 | T1 | P-val <= 0.05 |
| COL17A1 | 1.558627 | 5.358002 | 2.35E-08 | 8.26E-06 | T1 | P-val <= 0.05 |
| MFAP4 | 1.743656 | 4.423832 | 2.50E-08 | 8.59E-06 | T1 | P-val <= 0.05 |
| VASN | 1.62181 | 2.818976 | 3.51E-08 | 1.07E-05 | T1 | P-val <= 0.05 |
| ANKRD20A19P | 2.548423 | 2.115338 | 3.82E-08 | 1.13E-05 | T1 | P-val <= 0.05 |
| GAS6 | 1.606184 | 6.860648 | 4.29E-08 | 1.23E-05 | T1 | P-val <= 0.05 |
| COL9A3 | 2.236479 | 2.666913 | 4.83E-08 | 1.34E-05 | T1 | P-val <= 0.05 |
| PMP22 | 1.538792 | 4.512289 | 5.01E-08 | 1.37E-05 | T1 | P-val <= 0.05 |
| CPXM1 | 1.616241 | 4.212483 | 5.68E-08 | 1.45E-05 | T1 | P-val <= 0.05 |
| FAM107A | 1.838233 | 5.221385 | 7.11E-08 | 1.71E-05 | T1 | P-val <= 0.05 |
| ELFN1 | 1.532188 | 2.898933 | 7.86E-08 | 1.83E-05 | T1 | P-val <= 0.05 |
| COL2A1 | 1.529957 | 1.67463 | 8.81E-08 | 1.95E-05 | T1 | P-val <= 0.05 |
| RARRES2 | 1.871809 | 3.956026 | 1.11E-07 | 2.30E-05 | T1 | P-val <= 0.05 |
| AACSP1 | 1.828241 | 1.745477 | 1.14E-07 | 2.33E-05 | T1 | P-val <= 0.05 |
| FBN3 | 1.566073 | 1.63176 | 1.21E-07 | 2.44E-05 | T1 | P-val <= 0.05 |
| COL26A1 | 1.642272 | 2.255255 | 1.57E-07 | 2.88E-05 | T1 | P-val <= 0.05 |
| HEY1 | 1.517236 | 2.570727 | 1.63E-07 | 2.96E-05 | T1 | P-val <= 0.05 |
| C1QTNF9B | 2.031583 | 1.697617 | 1.71E-07 | 3.06E-05 | T1 | P-val <= 0.05 |
| P3H2 | 1.521776 | 3.03834 | 1.83E-07 | 3.22E-05 | T1 | P-val <= 0.05 |
| IRX4 | 1.533514 | 1.413661 | 1.88E-07 | 3.27E-05 | T1 | P-val <= 0.05 |
| ID4 | 1.507883 | 2.200277 | 2.46E-07 | 4.02E-05 | T1 | P-val <= 0.05 |
| GPC4 | 1.663286 | 5.062694 | 3.37E-07 | 5.17E-05 | T1 | P-val <= 0.05 |
| THBS4 | 1.534709 | 2.667974 | 3.60E-07 | 5.41E-05 | T1 | P-val <= 0.05 |
| MDFI | 1.622621 | 3.862127 | 3.89E-07 | 5.74E-05 | T1 | P-val <= 0.05 |
| CTGF/CCN2 | 2.249434 | 5.508548 | 3.93E-07 | 5.74E-05 | T1 | P-val <= 0.05 |
| PLTP | 1.527139 | 9.707795 | 4.05E-07 | 5.83E-05 | T1 | P-val <= 0.05 |
| NKX2-4 | 1.729659 | 1.435884 | 4.21E-07 | 5.97E-05 | T1 | P-val <= 0.05 |
| AMOTL2 | 1.689808 | 2.692504 | 4.75E-07 | 6.45E-05 | T1 | P-val <= 0.05 |
| MAOA | 1.700109 | 3.126434 | 6.15E-07 | 7.64E-05 | T1 | P-val <= 0.05 |
| KLF2P1 | 1.692361 | 1.699174 | 6.18E-07 | 7.64E-05 | T1 | P-val <= 0.05 |
| FRZB | 1.761942 | 2.945408 | 8.16E-07 | 9.23E-05 | T1 | P-val <= 0.05 |
| RAMP1 | 2.114959 | 4.389746 | 8.73E-07 | 9.74E-05 | T1 | P-val <= 0.05 |
| KANK2 | 1.550996 | 3.9763 | 9.01E-07 | 0.0001 | T1 | P-val <= 0.05 |
| SBSPON | 1.500227 | 1.953345 | 1.04E-06 | 0.000111 | T1 | P-val <= 0.05 |
| AQP5 | 2.647483 | 3.30922 | 1.05E-06 | 0.000111 | T1 | P-val <= 0.05 |
| IRX2 | 1.922462 | 2.119708 | 1.20E-06 | 0.00012 | T1 | P-val <= 0.05 |
| SLPI | 2.796561 | 5.153698 | 1.60E-06 | 0.00015 | T1 | P-val <= 0.05 |
| CPE | 1.619769 | 5.751459 | 1.69E-06 | 0.000156 | T1 | P-val <= 0.05 |
| SGCE | 1.570623 | 3.291855 | 1.94E-06 | 0.000173 | T1 | P-val <= 0.05 |
| PHF2P2 | 1.779766 | 1.379767 | 1.96E-06 | 0.000174 | T1 | P-val <= 0.05 |
| SAA1 | 1.960749 | 6.487328 | 2.40E-06 | 0.000199 | T1 | P-val <= 0.05 |
| TDGF1 | 1.780662 | 3.089697 | 4.16E-06 | 0.000304 | T1 | P-val <= 0.05 |
| PRSS16 | 2.153753 | 7.797944 | 6.82E-06 | 0.000422 | T1 | P-val <= 0.05 |
| WFDC2 | 1.833973 | 4.952386 | 1.20E-05 | 0.000637 | T1 | P-val <= 0.05 |
| SCARA3 | 1.61332 | 4.138271 | 2.58E-05 | 0.001119 | T1 | P-val <= 0.05 |
| TBATA | 1.854322 | 7.788226 | 3.20E-05 | 0.001295 | T1 | P-val <= 0.05 |
| PTGDS | 1.604451 | 5.329711 | 0.000107 | 0.003149 | T1 | P-val <= 0.05 |
| FN1 | 1.633378 | 4.963272 | 0.000376 | 0.007802 | T1 | P-val <= 0.05 |
| IRS4 | 2.148796 | 0.948359 | 6.41E-14 | 6.23E-10 | T2 | P-val <= 0.05 |
| ENPP3 | 2.533738 | 1.99834 | 1.23E-11 | 5.15E-08 | T2 | P-val <= 0.05 |
| CMBL | 1.718217 | 2.568767 | 2.46E-11 | 8.47E-08 | T2 | P-val <= 0.05 |
| LRTM2 | 2.353433 | 1.034656 | 3.55E-11 | 1.05E-07 | T2 | P-val <= 0.05 |
| NEFM | 2.555562 | 1.532099 | 3.61E-11 | 1.05E-07 | T2 | P-val <= 0.05 |
| AKR1B10 | 2.431823 | 1.824905 | 4.38E-11 | 1.06E-07 | T2 | P-val <= 0.05 |
| C4orf50 | 1.636191 | 0.898305 | 5.29E-11 | 1.14E-07 | T2 | P-val <= 0.05 |
| IL13RA2 | 2.015197 | 1.536575 | 6.28E-11 | 1.27E-07 | T2 | P-val <= 0.05 |
| TUSC8 | 1.968557 | 1.365399 | 1.02E-10 | 1.75E-07 | T2 | P-val <= 0.05 |
| CTB-14A14.2 | 1.691779 | 1.326991 | 4.87E-10 | 6.62E-07 | T2 | P-val <= 0.05 |
| NEFL | 3.500294 | 2.624167 | 5.04E-10 | 6.69E-07 | T2 | P-val <= 0.05 |
| LRRC17 | 1.748698 | 1.797771 | 1.18E-09 | 1.19E-06 | T2 | P-val <= 0.05 |
| RNF128 | 1.755841 | 1.477747 | 1.86E-09 | 1.45E-06 | T2 | P-val <= 0.05 |
| LRRN3 | 1.62021 | 1.506009 | 2.25E-09 | 1.66E-06 | T2 | P-val <= 0.05 |
| SPOCK1 | 2.48867 | 1.766591 | 2.36E-09 | 1.68E-06 | T2 | P-val <= 0.05 |
| ATP10B | 1.733654 | 1.112014 | 6.26E-09 | 3.55E-06 | T2 | P-val <= 0.05 |
| CTSV | 3.025056 | 5.900452 | 1.00E-08 | 5.10E-06 | T2 | P-val <= 0.05 |
| GPT2 | 1.525938 | 2.671722 | 3.25E-08 | 1.19E-05 | T2 | P-val <= 0.05 |
| PDE1A | 1.574643 | 1.228101 | 3.97E-08 | 1.36E-05 | T2 | P-val <= 0.05 |
| DSG3 | 2.197185 | 1.646742 | 4.14E-08 | 1.41E-05 | T2 | P-val <= 0.05 |
| TFF3 | 1.953658 | 2.6622 | 5.70E-08 | 1.75E-05 | T2 | P-val <= 0.05 |
| CXCL12 | 1.662516 | 4.750762 | 6.03E-08 | 1.81E-05 | T2 | P-val <= 0.05 |
| HSPA2 | 1.93697 | 2.433426 | 1.09E-07 | 2.75E-05 | T2 | P-val <= 0.05 |
| PLA2G4F | 1.560138 | 2.025075 | 7.21E-07 | 9.93E-05 | T2 | P-val <= 0.05 |
| GPR87 | 1.585695 | 1.313154 | 1.24E-06 | 0.000148 | T2 | P-val <= 0.05 |
| ASS1 | 1.59311 | 4.238533 | 5.79E-06 | 0.000452 | T2 | P-val <= 0.05 |
| KRT15 | 2.515722 | 3.692261 | 7.32E-06 | 0.000529 | T2 | P-val <= 0.05 |
| NUPR1 | 1.778696 | 2.711947 | 1.14E-05 | 0.000726 | T2 | P-val <= 0.05 |
| HSPB8 | 1.587982 | 4.059224 | 1.31E-05 | 0.000799 | T2 | P-val <= 0.05 |
| GPNMB | 1.92058 | 3.656931 | 1.36E-05 | 0.000822 | T2 | P-val <= 0.05 |
| PTN | 2.113544 | 3.72457 | 1.71E-05 | 0.00097 | T2 | P-val <= 0.05 |
| PCP4L1 | 1.525555 | 1.57626 | 1.72E-05 | 0.000975 | T2 | P-val <= 0.05 |
| MYH8 | 1.965491 | 1.69701 | 2.56E-05 | 0.001305 | T2 | P-val <= 0.05 |
| RHBDL1 | 1.535768 | 2.495398 | 6.11E-05 | 0.002457 | T2 | P-val <= 0.05 |
| TIMD4 | 1.52901 | 1.881904 | 6.56E-05 | 0.002586 | T2 | P-val <= 0.05 |
| SIX2 | 1.749118 | 3.262334 | 6.62E-05 | 0.002603 | T2 | P-val <= 0.05 |
| S100A9 | 2.012467 | 4.252142 | 0.000103 | 0.003589 | T2 | P-val <= 0.05 |
| ZBED2 | 1.549877 | 4.954583 | 0.00017 | 0.005165 | T2 | P-val <= 0.05 |
| CCL19 | 1.816026 | 6.481893 | 0.001768 | 0.028144 | T2 | P-val <= 0.05 |
| SOD3 | 1.55819 | 2.510625 | 0.001869 | 0.029164 | T2 | P-val <= 0.05 |
| CBLC | 3.545575 | 0.432766 | 1.28E-43 | 7.48E-39 | T3 | P-val <= 0.05 |
| CCL20 | 5.376025 | 0.811696 | 3.63E-37 | 1.06E-32 | T3 | P-val <= 0.05 |
| PLEKHG7 | 1.932816 | 0.194918 | 5.07E-36 | 9.86E-32 | T3 | P-val <= 0.05 |
| KIAA1549L | 2.1934 | 0.353303 | 9.57E-35 | 1.40E-30 | T3 | P-val <= 0.05 |
| PTGIR | 3.414096 | 0.898377 | 5.77E-32 | 5.40E-28 | T3 | P-val <= 0.05 |
| PROX1 | 1.849254 | 0.243662 | 6.48E-32 | 5.40E-28 | T3 | P-val <= 0.05 |
| GFI1B | 3.125157 | 0.376179 | 9.32E-32 | 6.80E-28 | T3 | P-val <= 0.05 |
| UGT8 | 1.761436 | 0.243444 | 2.35E-31 | 1.37E-27 | T3 | P-val <= 0.05 |
| HOXC9 | 2.406615 | 0.319454 | 2.55E-30 | 1.35E-26 | T3 | P-val <= 0.05 |
| CTA-126B4.7 | 2.492136 | 0.761419 | 1.12E-29 | 5.47E-26 | T3 | P-val <= 0.05 |
| MSLN | 5.635131 | 0.887954 | 1.99E-29 | 8.95E-26 | T3 | P-val <= 0.05 |
| KIT | 4.430711 | 1.166247 | 6.34E-29 | 2.65E-25 | T3 | P-val <= 0.05 |
| SLC9A7 | 1.765432 | 1.362786 | 2.33E-28 | 8.49E-25 | T3 | P-val <= 0.05 |
| MSLNL | 2.505176 | 0.270533 | 2.10E-26 | 5.84E-23 | T3 | P-val <= 0.05 |
| ASCL4 | 2.165212 | 0.336632 | 2.93E-26 | 7.78E-23 | T3 | P-val <= 0.05 |
| HOXC6 | 2.213949 | 0.412743 | 4.37E-26 | 1.11E-22 | T3 | P-val <= 0.05 |
| OXGR1 | 3.274403 | 0.741008 | 1.06E-25 | 2.38E-22 | T3 | P-val <= 0.05 |
| PLA2G3 | 1.630931 | 0.174918 | 8.91E-25 | 1.73E-21 | T3 | P-val <= 0.05 |
| POU2F3 | 2.591641 | 0.57779 | 1.01E-24 | 1.91E-21 | T3 | P-val <= 0.05 |
| TMEM151B | 1.630382 | 0.224091 | 1.45E-24 | 2.41E-21 | T3 | P-val <= 0.05 |
| RAB27B | 1.821298 | 0.531796 | 2.51E-24 | 3.96E-21 | T3 | P-val <= 0.05 |
| PITX1 | 2.809344 | 0.39575 | 6.16E-24 | 9.47E-21 | T3 | P-val <= 0.05 |
| CD70 | 2.685647 | 0.807919 | 7.54E-24 | 1.12E-20 | T3 | P-val <= 0.05 |
| ARNT2 | 2.425394 | 0.96851 | 7.68E-24 | 1.12E-20 | T3 | P-val <= 0.05 |
| PPP1R1B | 3.573196 | 0.837738 | 1.14E-23 | 1.63E-20 | T3 | P-val <= 0.05 |
| MYBPC1 | 3.939354 | 0.426088 | 2.19E-23 | 3.05E-20 | T3 | P-val <= 0.05 |
| FAM222A | 1.731638 | 0.623301 | 5.59E-23 | 7.09E-20 | T3 | P-val <= 0.05 |
| SPIC | 3.250195 | 0.820261 | 1.77E-22 | 2.02E-19 | T3 | P-val <= 0.05 |
| CD24 | 4.31901 | 1.562164 | 2.55E-22 | 2.80E-19 | T3 | P-val <= 0.05 |
| LRRC16B | 1.548625 | 0.690928 | 1.21E-21 | 1.18E-18 | T3 | P-val <= 0.05 |
| KRT80 | 1.574089 | 0.312401 | 1.61E-21 | 1.52E-18 | T3 | P-val <= 0.05 |
| GJB4 | 1.811501 | 0.404886 | 2.15E-21 | 1.96E-18 | T3 | P-val <= 0.05 |
| HOXC-AS1 | 1.791285 | 0.240109 | 2.41E-21 | 2.12E-18 | T3 | P-val <= 0.05 |
| HOXC8 | 2.644153 | 0.482275 | 2.43E-21 | 2.12E-18 | T3 | P-val <= 0.05 |
| NUAK2 | 1.919741 | 1.72662 | 3.21E-21 | 2.68E-18 | T3 | P-val <= 0.05 |
| TUBB2B | 3.139696 | 0.713954 | 4.51E-21 | 3.71E-18 | T3 | P-val <= 0.05 |
| GNAZ | 2.16655 | 0.820347 | 5.94E-21 | 4.69E-18 | T3 | P-val <= 0.05 |
| MYEOV | 1.877127 | 0.331873 | 8.60E-21 | 6.61E-18 | T3 | P-val <= 0.05 |
| HCK | 3.058291 | 3.177692 | 9.31E-21 | 7.06E-18 | T3 | P-val <= 0.05 |
| ELF3 | 3.290632 | 1.186069 | 1.86E-20 | 1.31E-17 | T3 | P-val <= 0.05 |
| MOCOS | 1.573996 | 0.541973 | 2.07E-20 | 1.44E-17 | T3 | P-val <= 0.05 |
| PLAU | 2.890067 | 2.054318 | 2.18E-20 | 1.48E-17 | T3 | P-val <= 0.05 |
| INPP5J | 2.418968 | 0.664661 | 2.18E-20 | 1.48E-17 | T3 | P-val <= 0.05 |
| PPFIBP2 | 2.059233 | 1.382153 | 2.58E-20 | 1.67E-17 | T3 | P-val <= 0.05 |
| ANKRD13B | 1.824375 | 1.698876 | 2.62E-20 | 1.68E-17 | T3 | P-val <= 0.05 |
| HEPACAM2 | 3.091774 | 0.639935 | 3.43E-20 | 2.10E-17 | T3 | P-val <= 0.05 |
| C15orf48 | 2.70863 | 1.206019 | 3.45E-20 | 2.10E-17 | T3 | P-val <= 0.05 |
| TMPRSS4 | 1.958366 | 0.285609 | 3.58E-20 | 2.15E-17 | T3 | P-val <= 0.05 |
| DUSP5 | 2.39101 | 1.962754 | 4.04E-20 | 2.38E-17 | T3 | P-val <= 0.05 |
| KEL | 1.898583 | 0.695549 | 5.72E-20 | 3.27E-17 | T3 | P-val <= 0.05 |
| ETV4 | 1.864804 | 0.569585 | 2.05E-19 | 1.08E-16 | T3 | P-val <= 0.05 |
| FOXA1 | 2.020933 | 0.304878 | 2.48E-19 | 1.29E-16 | T3 | P-val <= 0.05 |
| PADI3 | 1.63582 | 0.225282 | 2.64E-19 | 1.36E-16 | T3 | P-val <= 0.05 |
| MEX3A | 1.663292 | 1.023582 | 3.50E-19 | 1.76E-16 | T3 | P-val <= 0.05 |
| PAPSS2 | 2.174514 | 1.365243 | 7.28E-19 | 3.46E-16 | T3 | P-val <= 0.05 |
| PTGS2 | 2.297404 | 0.575447 | 1.14E-18 | 5.30E-16 | T3 | P-val <= 0.05 |
| ADTRP | 1.512708 | 0.503911 | 1.29E-18 | 5.93E-16 | T3 | P-val <= 0.05 |
| SULF1 | 2.57914 | 1.277372 | 2.34E-18 | 9.93E-16 | T3 | P-val <= 0.05 |
| SLC12A8 | 1.682392 | 0.695473 | 2.93E-18 | 1.22E-15 | T3 | P-val <= 0.05 |
| MUC4 | 1.60581 | 0.31061 | 3.48E-18 | 1.43E-15 | T3 | P-val <= 0.05 |
| GRB7 | 2.513637 | 1.274472 | 4.12E-18 | 1.66E-15 | T3 | P-val <= 0.05 |
| DHRS9 | 2.676518 | 0.450826 | 5.46E-18 | 2.14E-15 | T3 | P-val <= 0.05 |
| AACS | 1.597257 | 1.915664 | 9.96E-18 | 3.78E-15 | T3 | P-val <= 0.05 |
| GPT | 2.130057 | 0.93215 | 1.29E-17 | 4.73E-15 | T3 | P-val <= 0.05 |
| TSPAN17 | 1.626431 | 2.659738 | 1.91E-17 | 6.81E-15 | T3 | P-val <= 0.05 |
| THBS2 | 2.592223 | 1.19432 | 2.62E-17 | 8.96E-15 | T3 | P-val <= 0.05 |
| CDC42EP1 | 3.023523 | 2.725554 | 3.25E-17 | 1.07E-14 | T3 | P-val <= 0.05 |
| KCNN3 | 1.782293 | 0.585378 | 3.43E-17 | 1.13E-14 | T3 | P-val <= 0.05 |
| TMEM132A | 2.741661 | 1.73896 | 3.98E-17 | 1.29E-14 | T3 | P-val <= 0.05 |
| HOXC4 | 2.037358 | 0.710678 | 4.72E-17 | 1.51E-14 | T3 | P-val <= 0.05 |
| SLC39A11 | 1.504132 | 1.780266 | 5.00E-17 | 1.58E-14 | T3 | P-val <= 0.05 |
| BRI3BP | 1.542734 | 2.171402 | 6.06E-17 | 1.89E-14 | T3 | P-val <= 0.05 |
| ADAM8 | 2.326889 | 2.055163 | 8.04E-17 | 2.44E-14 | T3 | P-val <= 0.05 |
| TNFRSF11B | 1.666016 | 0.354427 | 8.41E-17 | 2.54E-14 | T3 | P-val <= 0.05 |
| INSM1 | 2.622407 | 0.858866 | 9.09E-17 | 2.70E-14 | T3 | P-val <= 0.05 |
| SOX2 | 3.044282 | 0.795625 | 1.07E-16 | 3.15E-14 | T3 | P-val <= 0.05 |
| FGD5 | 2.3253 | 1.444564 | 1.21E-16 | 3.53E-14 | T3 | P-val <= 0.05 |
| FNDC1 | 1.509546 | 0.373756 | 1.59E-16 | 4.60E-14 | T3 | P-val <= 0.05 |
| RAPGEFL1 | 2.424561 | 1.907881 | 1.68E-16 | 4.84E-14 | T3 | P-val <= 0.05 |
| MISP | 2.8452 | 0.971924 | 3.15E-16 | 8.46E-14 | T3 | P-val <= 0.05 |
| TNFAIP6 | 1.534073 | 0.526633 | 4.44E-16 | 1.16E-13 | T3 | P-val <= 0.05 |
| QPCT | 1.791909 | 1.372481 | 6.06E-16 | 1.56E-13 | T3 | P-val <= 0.05 |
| NFKBIE | 1.725474 | 3.326837 | 7.24E-16 | 1.82E-13 | T3 | P-val <= 0.05 |
| IL4I1 | 2.258911 | 1.634223 | 7.97E-16 | 1.98E-13 | T3 | P-val <= 0.05 |
| MROH6 | 1.616935 | 1.195235 | 1.62E-15 | 3.80E-13 | T3 | P-val <= 0.05 |
| TNFSF9 | 2.037417 | 0.806043 | 1.93E-15 | 4.50E-13 | T3 | P-val <= 0.05 |
| PAX5 | 1.886946 | 0.468217 | 2.23E-15 | 5.12E-13 | T3 | P-val <= 0.05 |
| SLC2A1 | 1.829909 | 2.80002 | 2.53E-15 | 5.70E-13 | T3 | P-val <= 0.05 |
| GJB3 | 2.388714 | 0.951177 | 2.77E-15 | 6.22E-13 | T3 | P-val <= 0.05 |
| CTSC | 1.685183 | 2.982179 | 3.04E-15 | 6.67E-13 | T3 | P-val <= 0.05 |
| TNFRSF4 | 2.546669 | 2.178529 | 3.92E-15 | 8.39E-13 | T3 | P-val <= 0.05 |
| CXCL13 | 4.002681 | 1.602666 | 3.96E-15 | 8.44E-13 | T3 | P-val <= 0.05 |
| LAG3 | 2.40319 | 1.374674 | 6.91E-15 | 1.39E-12 | T3 | P-val <= 0.05 |
| BSPRY | 1.942347 | 1.360286 | 8.03E-15 | 1.59E-12 | T3 | P-val <= 0.05 |
| RGS16 | 2.263479 | 1.419818 | 1.14E-14 | 2.20E-12 | T3 | P-val <= 0.05 |
| DGAT2 | 1.640738 | 1.025057 | 4.34E-14 | 7.52E-12 | T3 | P-val <= 0.05 |
| SCNN1A | 2.258557 | 1.065622 | 4.48E-14 | 7.74E-12 | T3 | P-val <= 0.05 |
| SPIB | 2.775409 | 1.33722 | 4.74E-14 | 8.12E-12 | T3 | P-val <= 0.05 |
| IL17REL | 2.061551 | 0.807169 | 5.90E-14 | 9.73E-12 | T3 | P-val <= 0.05 |
| C11orf53 | 2.479224 | 0.673811 | 6.93E-14 | 1.13E-11 | T3 | P-val <= 0.05 |
| ADORA1 | 1.908832 | 0.710371 | 7.99E-14 | 1.30E-11 | T3 | P-val <= 0.05 |
| REEP1 | 1.599375 | 0.468145 | 9.08E-14 | 1.45E-11 | T3 | P-val <= 0.05 |
| UBD | 3.629042 | 1.879464 | 1.06E-13 | 1.65E-11 | T3 | P-val <= 0.05 |
| PPAP2C | 1.938295 | 1.317768 | 1.45E-13 | 2.19E-11 | T3 | P-val <= 0.05 |
| SOX8 | 1.510975 | 0.402113 | 2.19E-13 | 3.16E-11 | T3 | P-val <= 0.05 |
| EVPL | 1.976718 | 0.917066 | 3.20E-13 | 4.45E-11 | T3 | P-val <= 0.05 |
| OR2I1P | 2.951081 | 1.254417 | 3.36E-13 | 4.62E-11 | T3 | P-val <= 0.05 |
| TNFSF10 | 1.988492 | 2.794838 | 3.38E-13 | 4.63E-11 | T3 | P-val <= 0.05 |
| HTRA3 | 2.355866 | 1.218984 | 3.59E-13 | 4.86E-11 | T3 | P-val <= 0.05 |
| GGT6 | 2.134195 | 0.490843 | 4.96E-13 | 6.52E-11 | T3 | P-val <= 0.05 |
| RAB36 | 1.564282 | 0.804353 | 6.75E-13 | 8.69E-11 | T3 | P-val <= 0.05 |
| PVRL4 | 2.586725 | 1.181474 | 8.75E-13 | 1.11E-10 | T3 | P-val <= 0.05 |
| FABP6 | 2.164475 | 0.650935 | 8.93E-13 | 1.12E-10 | T3 | P-val <= 0.05 |
| DCLK2 | 1.929292 | 1.172647 | 9.36E-13 | 1.17E-10 | T3 | P-val <= 0.05 |
| TUFT1 | 2.131446 | 1.637865 | 1.02E-12 | 1.27E-10 | T3 | P-val <= 0.05 |
| CEMIP | 1.639687 | 0.643238 | 1.21E-12 | 1.47E-10 | T3 | P-val <= 0.05 |
| FGFR3 | 3.443264 | 2.427079 | 1.45E-12 | 1.73E-10 | T3 | P-val <= 0.05 |
| TC2N | 1.764504 | 2.224313 | 1.48E-12 | 1.77E-10 | T3 | P-val <= 0.05 |
| FOXI1 | 2.372128 | 0.597333 | 1.53E-12 | 1.81E-10 | T3 | P-val <= 0.05 |
| EEF2K | 1.644119 | 2.761769 | 1.78E-12 | 2.07E-10 | T3 | P-val <= 0.05 |
| RELT | 1.774988 | 1.843052 | 2.06E-12 | 2.37E-10 | T3 | P-val <= 0.05 |
| ECE1 | 2.007865 | 3.710481 | 2.12E-12 | 2.42E-10 | T3 | P-val <= 0.05 |
| SLC2A5 | 1.756275 | 0.92327 | 2.69E-12 | 3.01E-10 | T3 | P-val <= 0.05 |
| ATF5 | 2.265148 | 4.225517 | 3.24E-12 | 3.56E-10 | T3 | P-val <= 0.05 |
| PRR5L | 2.01796 | 1.578635 | 3.50E-12 | 3.79E-10 | T3 | P-val <= 0.05 |
| EMP2 | 1.932278 | 1.873554 | 3.87E-12 | 4.16E-10 | T3 | P-val <= 0.05 |
| WFDC21P | 2.81997 | 2.644871 | 3.96E-12 | 4.23E-10 | T3 | P-val <= 0.05 |
| CLDN4 | 3.333648 | 1.600275 | 4.73E-12 | 5.00E-10 | T3 | P-val <= 0.05 |
| COCH | 1.598802 | 0.495706 | 4.89E-12 | 5.16E-10 | T3 | P-val <= 0.05 |
| SYNGR2 | 1.658711 | 5.384171 | 5.23E-12 | 5.47E-10 | T3 | P-val <= 0.05 |
| CSF2RB | 1.721546 | 1.369997 | 5.76E-12 | 5.96E-10 | T3 | P-val <= 0.05 |
| COL5A2 | 2.049764 | 1.659447 | 6.01E-12 | 6.17E-10 | T3 | P-val <= 0.05 |
| DOC2B | 1.680475 | 0.895278 | 6.05E-12 | 6.21E-10 | T3 | P-val <= 0.05 |
| LY6D | 3.04961 | 0.571641 | 6.35E-12 | 6.42E-10 | T3 | P-val <= 0.05 |
| PYDC1 | 1.987903 | 0.310414 | 7.10E-12 | 7.05E-10 | T3 | P-val <= 0.05 |
| PDZK1IP1 | 1.832706 | 0.561794 | 8.20E-12 | 8.01E-10 | T3 | P-val <= 0.05 |
| PLEKHG6 | 1.755416 | 1.049228 | 8.41E-12 | 8.17E-10 | T3 | P-val <= 0.05 |
| BCL3 | 1.547376 | 3.179825 | 8.51E-12 | 8.24E-10 | T3 | P-val <= 0.05 |
| CA9 | 2.187866 | 0.732202 | 8.81E-12 | 8.52E-10 | T3 | P-val <= 0.05 |
| ANXA1 | 2.171572 | 4.128623 | 8.91E-12 | 8.60E-10 | T3 | P-val <= 0.05 |
| TAS1R3 | 2.557928 | 1.029733 | 9.32E-12 | 8.95E-10 | T3 | P-val <= 0.05 |
| NOV | 1.543864 | 0.985645 | 1.06E-11 | 1.00E-09 | T3 | P-val <= 0.05 |
| B3GNT8 | 1.541386 | 0.983266 | 1.15E-11 | 1.07E-09 | T3 | P-val <= 0.05 |
| RBM47 | 1.844715 | 2.009121 | 1.23E-11 | 1.14E-09 | T3 | P-val <= 0.05 |
| BCL2A1 | 2.303991 | 1.966068 | 1.31E-11 | 1.20E-09 | T3 | P-val <= 0.05 |
| C1orf53 | 1.629294 | 1.088746 | 1.58E-11 | 1.42E-09 | T3 | P-val <= 0.05 |
| TEAD4 | 1.88523 | 1.966229 | 1.89E-11 | 1.64E-09 | T3 | P-val <= 0.05 |
| CCL4 | 2.455413 | 1.803133 | 1.98E-11 | 1.72E-09 | T3 | P-val <= 0.05 |
| DLX5 | 2.257875 | 0.617239 | 3.09E-11 | 2.56E-09 | T3 | P-val <= 0.05 |
| PODXL2 | 1.872503 | 2.668822 | 3.37E-11 | 2.78E-09 | T3 | P-val <= 0.05 |
| ADAMDEC1 | 1.979667 | 0.986198 | 3.86E-11 | 3.15E-09 | T3 | P-val <= 0.05 |
| DEFB4A | 1.611313 | 0.259058 | 4.62E-11 | 3.72E-09 | T3 | P-val <= 0.05 |
| POSTN | 3.030181 | 1.671378 | 4.92E-11 | 3.95E-09 | T3 | P-val <= 0.05 |
| CLDN3 | 3.455861 | 1.703811 | 5.28E-11 | 4.19E-09 | T3 | P-val <= 0.05 |
| LUM | 3.622388 | 2.165698 | 5.45E-11 | 4.29E-09 | T3 | P-val <= 0.05 |
| FAM83H-AS1 | 1.906861 | 1.149 | 5.46E-11 | 4.30E-09 | T3 | P-val <= 0.05 |
| SGK1 | 1.887481 | 2.293571 | 7.93E-11 | 6.02E-09 | T3 | P-val <= 0.05 |
| FST | 2.472743 | 1.417052 | 8.13E-11 | 6.16E-09 | T3 | P-val <= 0.05 |
| SOX14 | 2.01569 | 0.301648 | 9.14E-11 | 6.86E-09 | T3 | P-val <= 0.05 |
| FAM129A | 1.963205 | 2.105524 | 1.01E-10 | 7.53E-09 | T3 | P-val <= 0.05 |
| PRR15L | 2.387599 | 0.813446 | 1.11E-10 | 8.20E-09 | T3 | P-val <= 0.05 |
| CHI3L1 | 3.331162 | 2.125952 | 1.18E-10 | 8.64E-09 | T3 | P-val <= 0.05 |
| KRT16 | 2.839346 | 0.924534 | 1.70E-10 | 1.18E-08 | T3 | P-val <= 0.05 |
| COL3A1 | 3.425104 | 3.641359 | 1.87E-10 | 1.28E-08 | T3 | P-val <= 0.05 |
| MECOM | 1.576197 | 0.967747 | 1.87E-10 | 1.28E-08 | T3 | P-val <= 0.05 |
| ASPN | 1.687066 | 0.917284 | 2.17E-10 | 1.46E-08 | T3 | P-val <= 0.05 |
| SPON1 | 1.588367 | 0.585169 | 2.26E-10 | 1.51E-08 | T3 | P-val <= 0.05 |
| SPTBN2 | 1.834976 | 1.278073 | 2.30E-10 | 1.53E-08 | T3 | P-val <= 0.05 |
| FXYD3 | 3.313255 | 1.740562 | 2.43E-10 | 1.61E-08 | T3 | P-val <= 0.05 |
| CKB | 2.956927 | 3.478622 | 2.59E-10 | 1.70E-08 | T3 | P-val <= 0.05 |
| PMAIP1 | 1.933623 | 2.575913 | 2.94E-10 | 1.89E-08 | T3 | P-val <= 0.05 |
| SLCO3A1 | 1.748426 | 1.625259 | 3.50E-10 | 2.21E-08 | T3 | P-val <= 0.05 |
| BIK | 1.973417 | 2.523826 | 3.53E-10 | 2.23E-08 | T3 | P-val <= 0.05 |
| FAM3B | 1.938537 | 1.558463 | 3.72E-10 | 2.33E-08 | T3 | P-val <= 0.05 |
| CREB3L1 | 2.53895 | 1.516524 | 3.76E-10 | 2.35E-08 | T3 | P-val <= 0.05 |
| STOM | 1.875321 | 4.80083 | 4.67E-10 | 2.83E-08 | T3 | P-val <= 0.05 |
| HAPLN3 | 1.671595 | 2.093876 | 5.11E-10 | 3.07E-08 | T3 | P-val <= 0.05 |
| COLCA2 | 2.397159 | 2.423027 | 6.47E-10 | 3.77E-08 | T3 | P-val <= 0.05 |
| ERBB3 | 1.794947 | 0.705897 | 7.39E-10 | 4.28E-08 | T3 | P-val <= 0.05 |
| MGAT3 | 2.391432 | 1.684857 | 8.25E-10 | 4.73E-08 | T3 | P-val <= 0.05 |
| CXCL11 | 1.971778 | 1.169454 | 8.43E-10 | 4.81E-08 | T3 | P-val <= 0.05 |
| FBP1 | 1.604009 | 1.443941 | 8.84E-10 | 5.02E-08 | T3 | P-val <= 0.05 |
| SMIM22 | 2.663733 | 0.91533 | 9.10E-10 | 5.13E-08 | T3 | P-val <= 0.05 |
| CTSK | 1.994388 | 2.37826 | 1.33E-09 | 7.22E-08 | T3 | P-val <= 0.05 |
| PDGFD | 1.500158 | 1.426134 | 1.71E-09 | 8.87E-08 | T3 | P-val <= 0.05 |
| TNFRSF18 | 2.164111 | 2.026812 | 1.77E-09 | 9.11E-08 | T3 | P-val <= 0.05 |
| SLAMF7 | 1.681049 | 1.695035 | 2.07E-09 | 1.04E-07 | T3 | P-val <= 0.05 |
| CELSR2 | 1.533394 | 1.664955 | 2.33E-09 | 1.16E-07 | T3 | P-val <= 0.05 |
| GSN | 1.948432 | 4.615324 | 2.50E-09 | 1.24E-07 | T3 | P-val <= 0.05 |
| C1orf115 | 1.507462 | 2.061268 | 3.21E-09 | 1.55E-07 | T3 | P-val <= 0.05 |
| COLCA1 | 1.671382 | 1.316943 | 3.45E-09 | 1.65E-07 | T3 | P-val <= 0.05 |
| CDKN1A | 1.694808 | 3.82375 | 4.22E-09 | 1.99E-07 | T3 | P-val <= 0.05 |
| C2orf54 | 1.513492 | 0.683876 | 4.81E-09 | 2.22E-07 | T3 | P-val <= 0.05 |
| MYC | 2.19269 | 4.642669 | 5.01E-09 | 2.31E-07 | T3 | P-val <= 0.05 |
| CD9 | 1.753728 | 5.173081 | 5.16E-09 | 2.36E-07 | T3 | P-val <= 0.05 |
| CCDC78 | 1.76211 | 1.651207 | 6.71E-09 | 2.97E-07 | T3 | P-val <= 0.05 |
| TJP3 | 1.539859 | 1.044066 | 7.17E-09 | 3.14E-07 | T3 | P-val <= 0.05 |
| C8orf4 | 1.791357 | 1.726954 | 7.23E-09 | 3.16E-07 | T3 | P-val <= 0.05 |
| CCR5 | 1.740498 | 1.420428 | 7.71E-09 | 3.32E-07 | T3 | P-val <= 0.05 |
| RELB | 1.509688 | 3.933555 | 1.06E-08 | 4.41E-07 | T3 | P-val <= 0.05 |
| MEOX1 | 1.801843 | 1.154084 | 1.07E-08 | 4.42E-07 | T3 | P-val <= 0.05 |
| COL5A1 | 2.110263 | 2.415806 | 1.08E-08 | 4.49E-07 | T3 | P-val <= 0.05 |
| TMEM40 | 1.661369 | 0.930404 | 1.64E-08 | 6.45E-07 | T3 | P-val <= 0.05 |
| CXCL10 | 2.848751 | 3.278131 | 1.71E-08 | 6.69E-07 | T3 | P-val <= 0.05 |
| BIRC3 | 1.718315 | 3.085907 | 1.94E-08 | 7.44E-07 | T3 | P-val <= 0.05 |
| CTHRC1 | 1.646913 | 2.114834 | 2.40E-08 | 9.00E-07 | T3 | P-val <= 0.05 |
| OASL | 1.693018 | 1.3693 | 2.45E-08 | 9.15E-07 | T3 | P-val <= 0.05 |
| GPRC5B | 2.065999 | 1.835777 | 2.66E-08 | 9.88E-07 | T3 | P-val <= 0.05 |
| GBP5 | 1.727333 | 1.236574 | 3.02E-08 | 1.10E-06 | T3 | P-val <= 0.05 |
| TLCD1 | 1.760766 | 2.226094 | 5.04E-08 | 1.73E-06 | T3 | P-val <= 0.05 |
| ADAM15 | 1.622936 | 2.98984 | 5.61E-08 | 1.89E-06 | T3 | P-val <= 0.05 |
| TMEM176A | 1.550418 | 2.586095 | 7.50E-08 | 2.43E-06 | T3 | P-val <= 0.05 |
| LA16c-321D4.2 | 1.712301 | 1.375577 | 7.71E-08 | 2.48E-06 | T3 | P-val <= 0.05 |
| MUC1 | 1.70654 | 1.149158 | 9.13E-08 | 2.86E-06 | T3 | P-val <= 0.05 |
| COMP | 2.021924 | 0.945993 | 1.50E-07 | 4.42E-06 | T3 | P-val <= 0.05 |
| HAGHL | 1.869393 | 1.986373 | 1.65E-07 | 4.81E-06 | T3 | P-val <= 0.05 |
| KRT13 | 1.581938 | 1.076641 | 2.04E-07 | 5.81E-06 | T3 | P-val <= 0.05 |
| TNFAIP2 | 1.710793 | 5.310384 | 2.14E-07 | 6.05E-06 | T3 | P-val <= 0.05 |
| TSPO | 1.635961 | 5.569653 | 2.23E-07 | 6.27E-06 | T3 | P-val <= 0.05 |
| PYCR1 | 1.932104 | 2.530374 | 2.78E-07 | 7.62E-06 | T3 | P-val <= 0.05 |
| ASCL3 | 1.603945 | 0.444886 | 3.15E-07 | 8.50E-06 | T3 | P-val <= 0.05 |
| S100A2 | 3.012372 | 2.861438 | 3.34E-07 | 8.95E-06 | T3 | P-val <= 0.05 |
| MFAP5 | 1.540403 | 0.615505 | 3.92E-07 | 1.02E-05 | T3 | P-val <= 0.05 |
| EHF | 1.903984 | 0.819888 | 4.00E-07 | 1.04E-05 | T3 | P-val <= 0.05 |
| COL1A1 | 2.980235 | 4.281177 | 4.73E-07 | 1.20E-05 | T3 | P-val <= 0.05 |
| PARM1 | 1.958676 | 3.418309 | 6.21E-07 | 1.52E-05 | T3 | P-val <= 0.05 |
| CCDC80 | 1.674862 | 1.108754 | 6.39E-07 | 1.56E-05 | T3 | P-val <= 0.05 |
| OLFML2B | 1.620207 | 1.974256 | 6.99E-07 | 1.68E-05 | T3 | P-val <= 0.05 |
| AZGP1 | 1.693319 | 0.67627 | 8.05E-07 | 1.91E-05 | T3 | P-val <= 0.05 |
| GBP4 | 1.540088 | 2.216234 | 8.14E-07 | 1.93E-05 | T3 | P-val <= 0.05 |
| MARCO | 1.530394 | 0.794699 | 8.39E-07 | 1.98E-05 | T3 | P-val <= 0.05 |
| GRASP | 1.881164 | 2.753125 | 8.40E-07 | 1.98E-05 | T3 | P-val <= 0.05 |
| TMEM176B | 1.547639 | 4.146705 | 9.16E-07 | 2.14E-05 | T3 | P-val <= 0.05 |
| OLFML3 | 1.767418 | 2.004227 | 1.01E-06 | 2.32E-05 | T3 | P-val <= 0.05 |
| HMGN2P15 | 1.663916 | 1.608749 | 1.61E-06 | 3.47E-05 | T3 | P-val <= 0.05 |
| GZMH | 1.871519 | 2.054587 | 1.82E-06 | 3.85E-05 | T3 | P-val <= 0.05 |
| JUNB | 1.711395 | 5.739378 | 2.26E-06 | 4.62E-05 | T3 | P-val <= 0.05 |
| KLF4 | 1.740662 | 1.832114 | 2.28E-06 | 4.66E-05 | T3 | P-val <= 0.05 |
| CCNO | 1.60331 | 0.851235 | 2.45E-06 | 4.96E-05 | T3 | P-val <= 0.05 |
| LAD1 | 1.893207 | 3.06235 | 2.49E-06 | 5.02E-05 | T3 | P-val <= 0.05 |
| RHOV | 2.176855 | 2.053671 | 2.84E-06 | 5.61E-05 | T3 | P-val <= 0.05 |
| PLA2G2A | 1.701972 | 0.511057 | 2.91E-06 | 5.74E-05 | T3 | P-val <= 0.05 |
| NID1 | 1.712211 | 2.149393 | 3.26E-06 | 6.31E-05 | T3 | P-val <= 0.05 |
| SPR | 1.513522 | 3.071081 | 3.40E-06 | 6.53E-05 | T3 | P-val <= 0.05 |
| ATF3 | 1.871455 | 2.017196 | 6.04E-06 | 0.000106 | T3 | P-val <= 0.05 |
| VPREB3 | 1.970539 | 1.528104 | 6.06E-06 | 0.000106 | T3 | P-val <= 0.05 |
| ANKRD22 | 1.882262 | 2.29541 | 6.33E-06 | 0.00011 | T3 | P-val <= 0.05 |
| C10orf10 | 1.653516 | 3.124858 | 1.06E-05 | 0.000172 | T3 | P-val <= 0.05 |
| PLA2G2D | 1.563869 | 1.071223 | 1.19E-05 | 0.000189 | T3 | P-val <= 0.05 |
| FHDC1 | 1.665976 | 1.341117 | 1.19E-05 | 0.00019 | T3 | P-val <= 0.05 |
| COL1A2 | 2.585321 | 3.828842 | 1.58E-05 | 0.000241 | T3 | P-val <= 0.05 |
| UPK1B | 1.586089 | 0.789134 | 2.18E-05 | 0.000318 | T3 | P-val <= 0.05 |
| ATP8B1 | 1.549541 | 1.56962 | 2.34E-05 | 0.000338 | T3 | P-val <= 0.05 |
| SELM | 1.737868 | 2.940131 | 2.38E-05 | 0.000343 | T3 | P-val <= 0.05 |
| SPP1 | 2.023934 | 1.874739 | 2.44E-05 | 0.000351 | T3 | P-val <= 0.05 |
| FCGR3A | 1.859776 | 2.957198 | 2.59E-05 | 0.000367 | T3 | P-val <= 0.05 |
| GZMB | 1.695642 | 2.260185 | 2.68E-05 | 0.000378 | T3 | P-val <= 0.05 |
| CCND2 | 1.639185 | 4.020878 | 3.93E-05 | 0.000523 | T3 | P-val <= 0.05 |
| HES6 | 1.875774 | 2.961378 | 3.95E-05 | 0.000525 | T3 | P-val <= 0.05 |
| BHLHE40 | 1.558225 | 3.967555 | 3.97E-05 | 0.000528 | T3 | P-val <= 0.05 |
| KLF5 | 1.830884 | 3.575852 | 3.99E-05 | 0.00053 | T3 | P-val <= 0.05 |
| NKG7 | 1.610303 | 3.667796 | 0.000103 | 0.001184 | T3 | P-val <= 0.05 |
| CXCL14 | 2.964325 | 2.611576 | 0.000109 | 0.001244 | T3 | P-val <= 0.05 |
| EPHX3 | 1.68995 | 2.096155 | 0.00011 | 0.001247 | T3 | P-val <= 0.05 |
| GZMK | 1.555514 | 2.022197 | 0.000115 | 0.0013 | T3 | P-val <= 0.05 |
| RASSF6 | 1.635127 | 2.468008 | 0.000207 | 0.002123 | T3 | P-val <= 0.05 |
| G0S2 | 1.523952 | 1.781919 | 0.000246 | 0.002451 | T3 | P-val <= 0.05 |
| TSPAN1 | 1.558005 | 1.521885 | 0.000273 | 0.002675 | T3 | P-val <= 0.05 |
| CCL5 | 1.690068 | 4.723333 | 0.00033 | 0.003154 | T3 | P-val <= 0.05 |
| FDCSP | 1.786405 | 0.949997 | 0.000571 | 0.004978 | T3 | P-val <= 0.05 |
| DHRS2 | 1.512352 | 0.776559 | 0.000607 | 0.005222 | T3 | P-val <= 0.05 |
| NNMT | 1.570045 | 2.675375 | 0.000629 | 0.005388 | T3 | P-val <= 0.05 |
| COL9A2 | 1.595955 | 2.480417 | 0.000635 | 0.00543 | T3 | P-val <= 0.05 |
| CXCL9 | 1.937674 | 3.139645 | 0.000642 | 0.005479 | T3 | P-val <= 0.05 |
| KRT18 | 1.554781 | 4.687814 | 0.000643 | 0.00549 | T3 | P-val <= 0.05 |
| GPR87 | 1.633403 | 1.313154 | 0.000769 | 0.00636 | T3 | P-val <= 0.05 |
| GADD45G | 1.654476 | 3.427979 | 0.00087 | 0.007058 | T3 | P-val <= 0.05 |
| KCNH2 | 1.57124 | 1.737173 | 0.000961 | 0.007657 | T3 | P-val <= 0.05 |
| PTGES | 1.841985 | 2.275374 | 0.001232 | 0.00937 | T3 | P-val <= 0.05 |
| EPCAM | 1.512825 | 3.200508 | 0.00166 | 0.011583 | T3 | P-val <= 0.05 |
| DPT | 1.568176 | 1.368203 | 0.00211 | 0.014059 | T3 | P-val <= 0.05 |
| FCN2 | 2.045495 | 1.97067 | 0.002457 | 0.015894 | T3 | P-val <= 0.05 |
| MIR8071-2 | 1.502947 | 1.556117 | 0.003041 | 0.018882 | T3 | P-val <= 0.05 |
| PRAME | 1.8084 | 2.239057 | 0.003171 | 0.019523 | T3 | P-val <= 0.05 |
| TACSTD2 | 1.528948 | 3.043215 | 0.009839 | 0.048764 | T3 | P-val <= 0.05 |

Table S6. List of antibodies

| Antibodies | Manufacturer | Catalog number | Dilution |
| --- | --- | --- | --- |
| KRT14 | Abcam | ab51054 | 1:100 |
| MYL9 | Abcam | ab191393 | 1:500 |
| CCN2/CTGF | Proteintech | 25474-1-AP | 1:100 |
| DNTT | Abcam | ab76544 | 1:250 |
| RAG2 | Proteintech | 11825-1-AP | 1:50 |
| CCL21 | Abcam | ab9851 | 1:500 |
| CD8 | Abcam | ab101500 | 1:100 |
| LAG3 | Abcam | ab209236 | 1:1000 |
| HAVCR2/TIM3 | Abcam | ab241332 | 1:1000 |
| PD1 | Abcam | ab52587 | 1:50 |
| CCL20 | Proteintech | 26527-1-AP | 1:500 |
| CD4 | Abcam | ab133616 | 1:600 |
| CCR6 | Abcam | ab303672 | 1:200 |
| FOXP3 | Abcam | ab215206 | 1:100 |
| CD163 | Abcam | ab182422 | 1:300 |
| GLUL | Abcam | ab176562 | 1:250 |
| MSLN | Abcam | ab196235 | 1:1500 |
| FOXI1 | Abcam | ab20454 | 1:1000 |
| KRT7 | Proteintech | 17513-1-AP | 1:1000 |
| CFTR | Abcam | ab131553 | 1:1000 |
| RARRES2 | Proteintech | 10216-1-AP | 1:500 |
| CMKLR1 | Abcam | ab306554 | 1:200 |
| TBX1 | Abcam | ab109313 | 1:200 |
| MHCII | Abcam | ab170867 | 1:200 |
| CCR7 | Abcam | ab253187 | 1:500 |

**High-resolution H&E, IHC and mIF images for Figures**

**
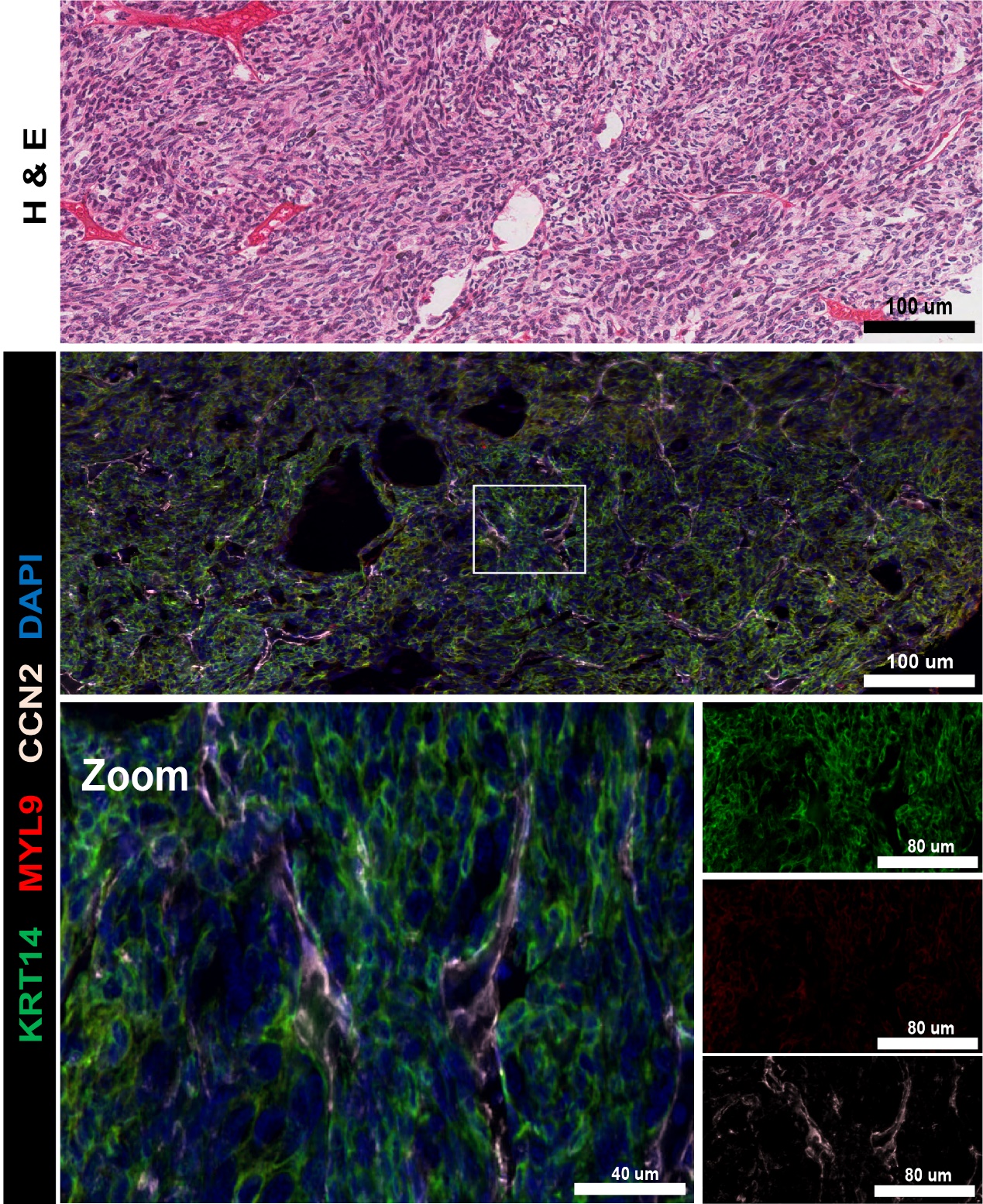
**

**High-resolution H&E and mIF image for Figure 1i.**

**
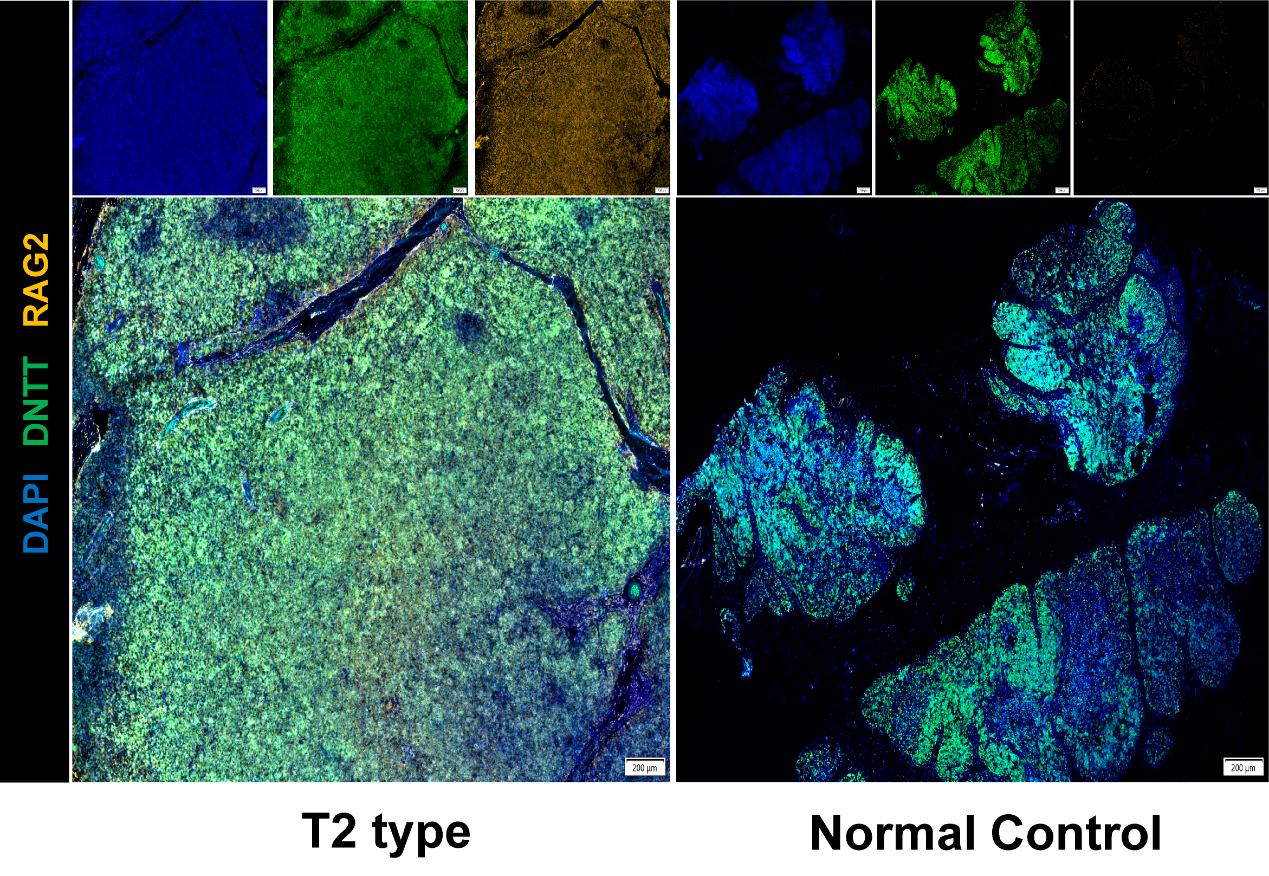
**

**High-resolution mIF image for Figure 3h.**

**
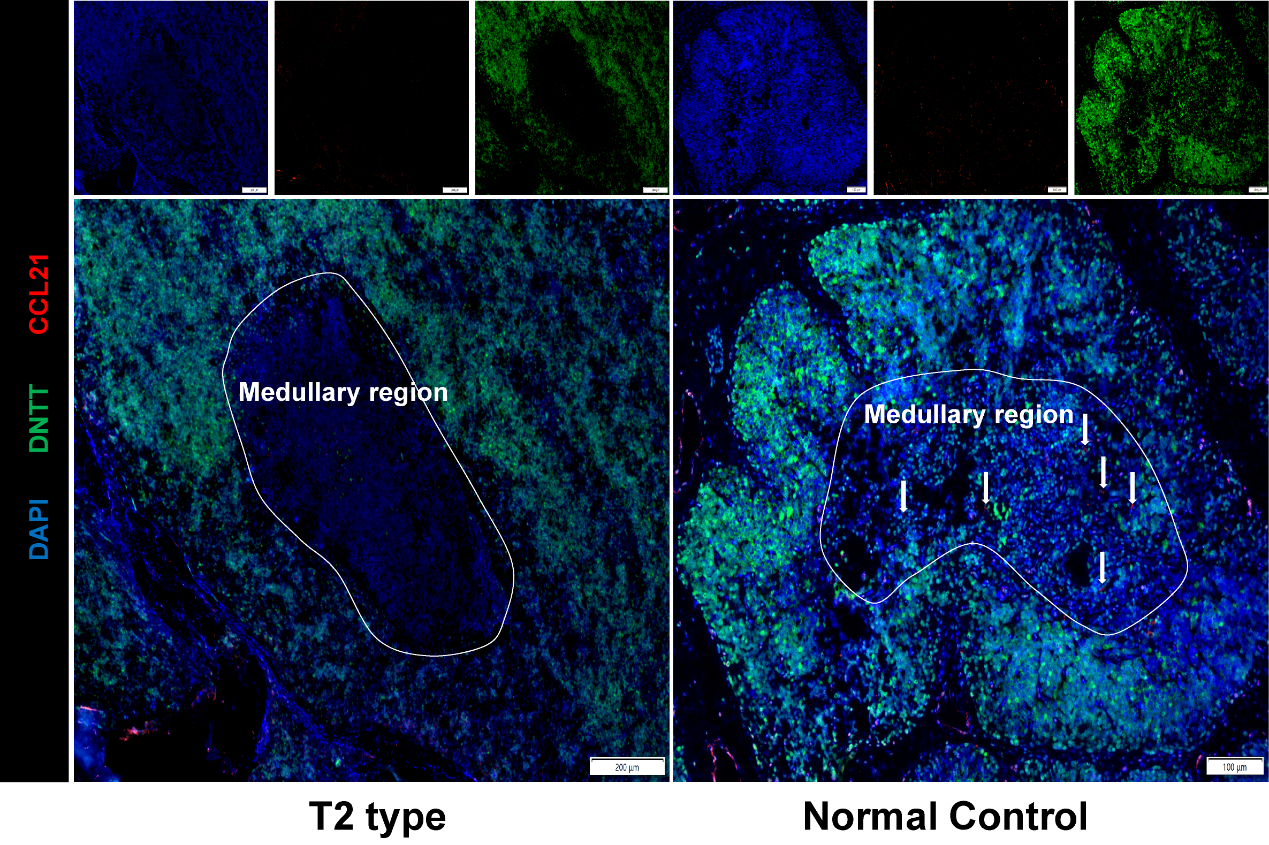
**

**High resolution mIF image for Figure. 3k.**

**
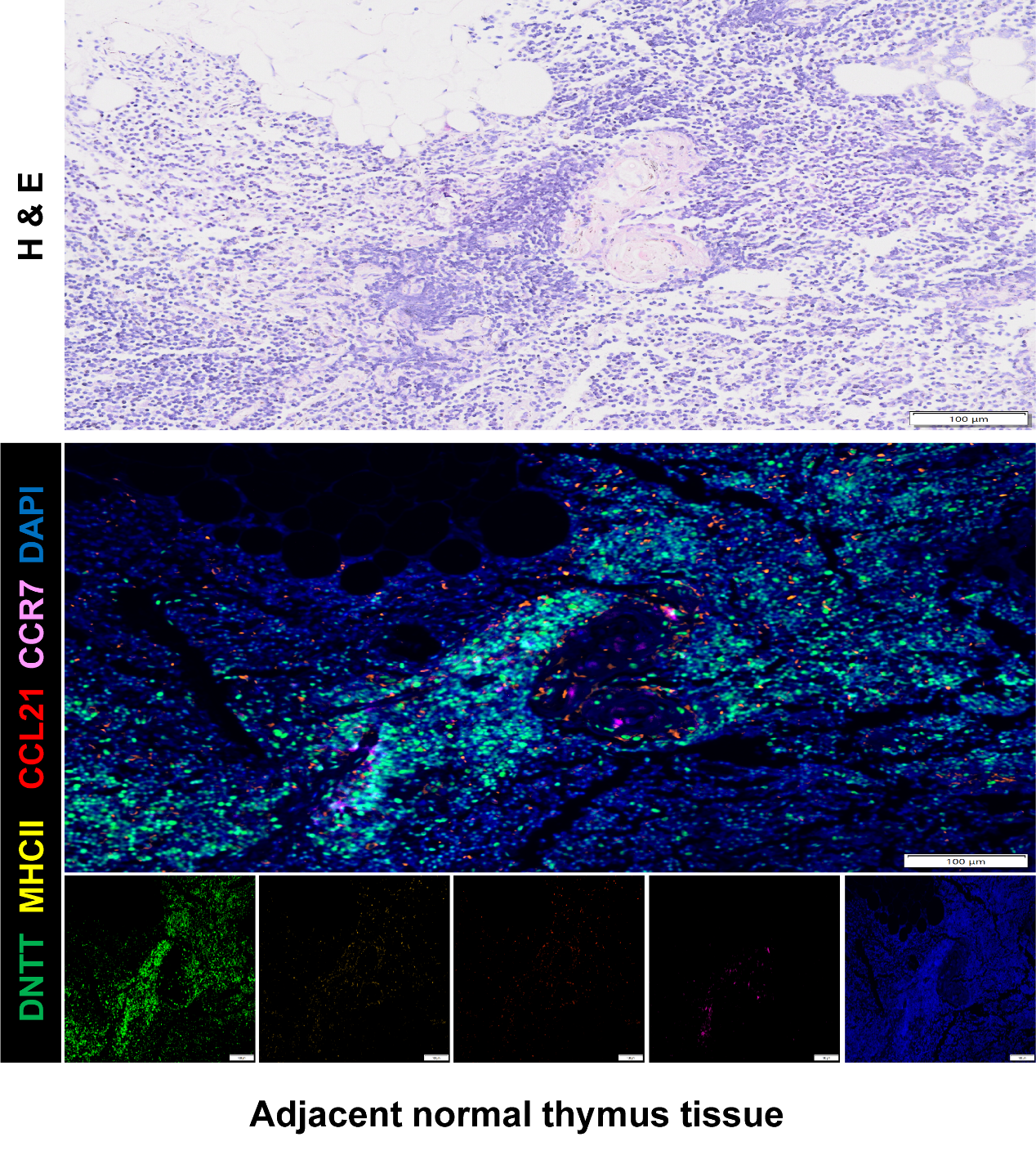
**

**High-resolution H&E and mIF images for Figure S10b.**

**
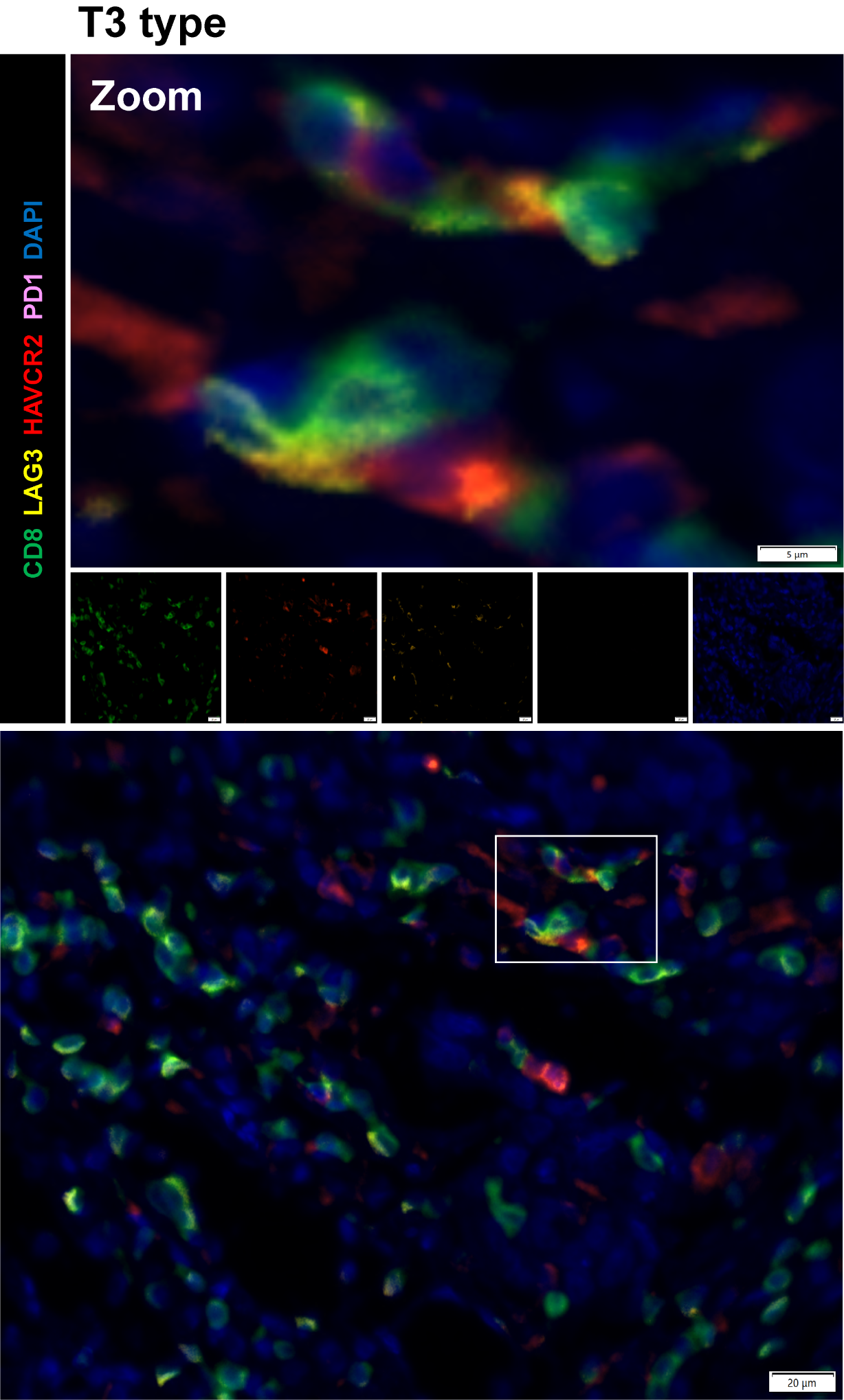
**

**High-resolution mIF image for Figure 4g.**

**
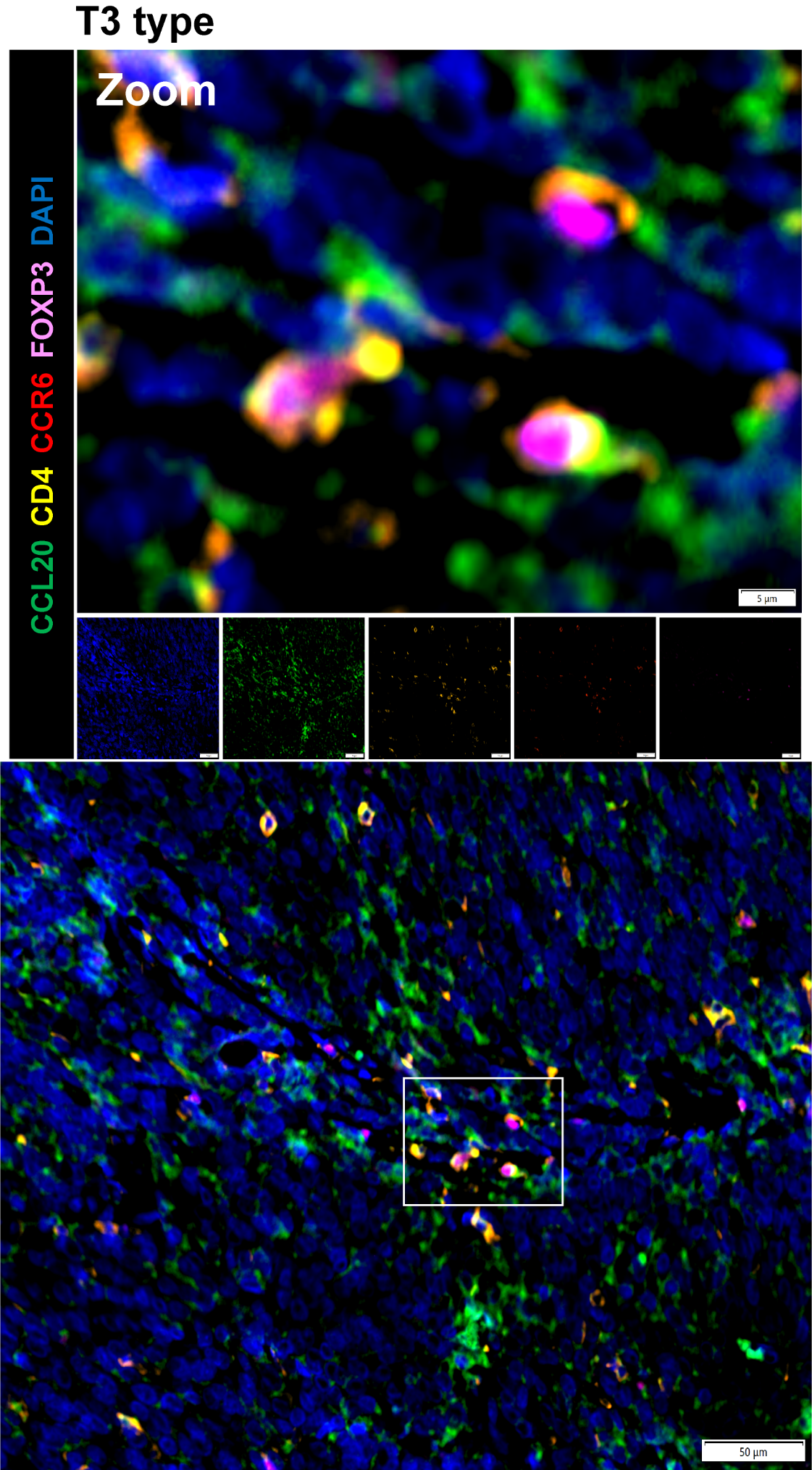
**

**High-resolution mIF image for Figure 4k.**

**
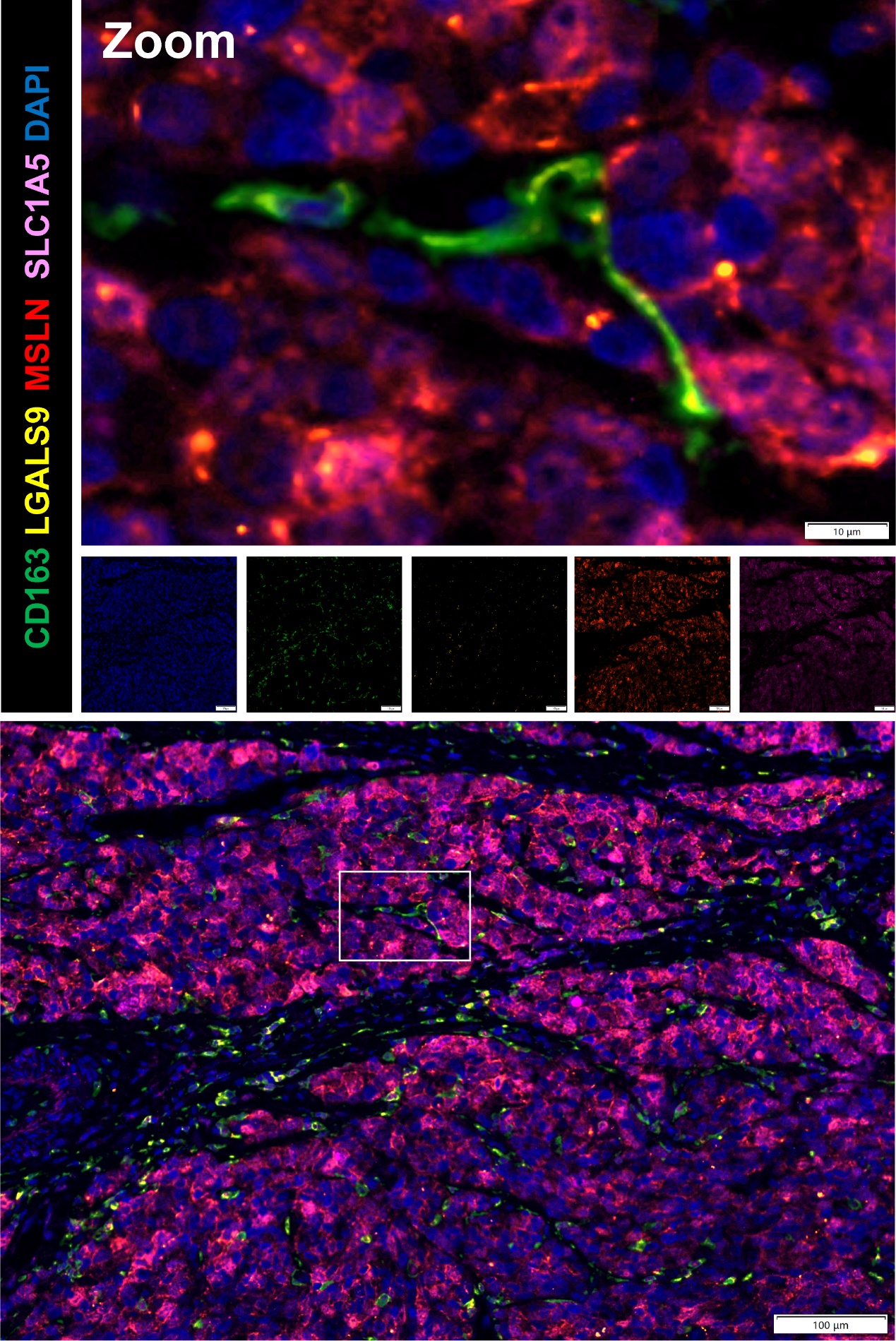
**

**High-resolution mIF image for Figure 5f.**

**
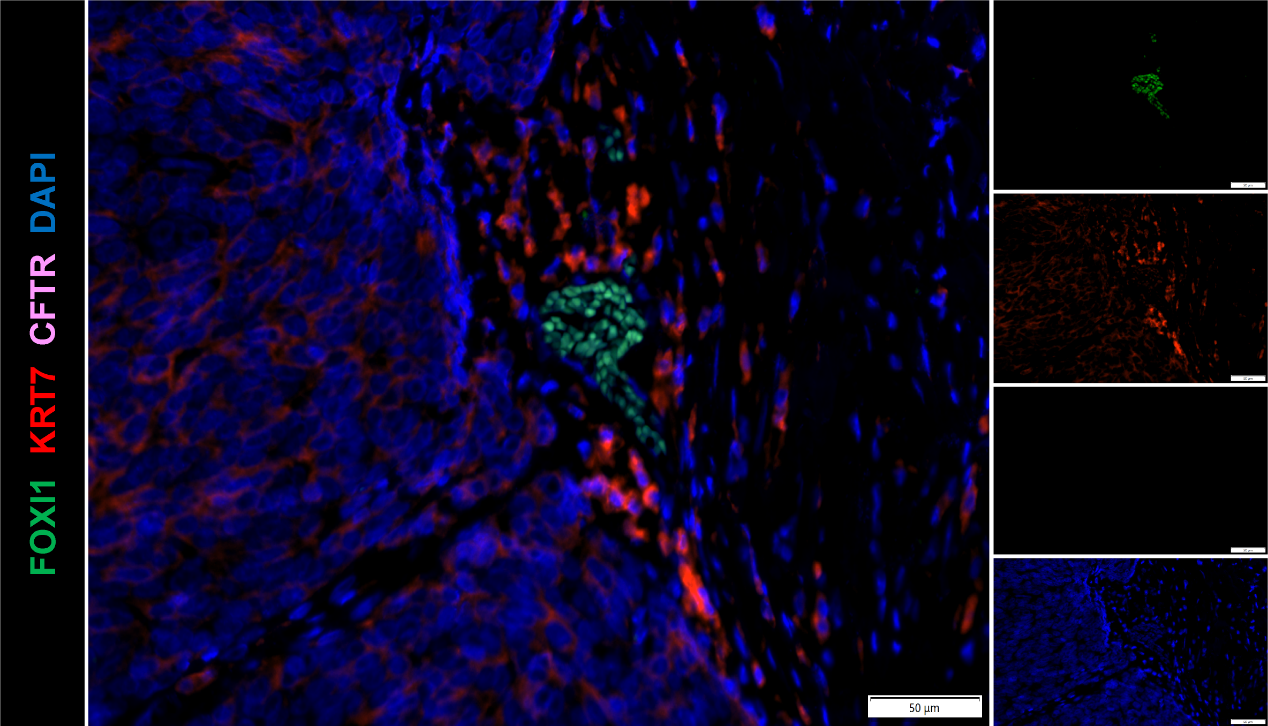
**

**High-resolution mIF image for Figure 6e.**

**
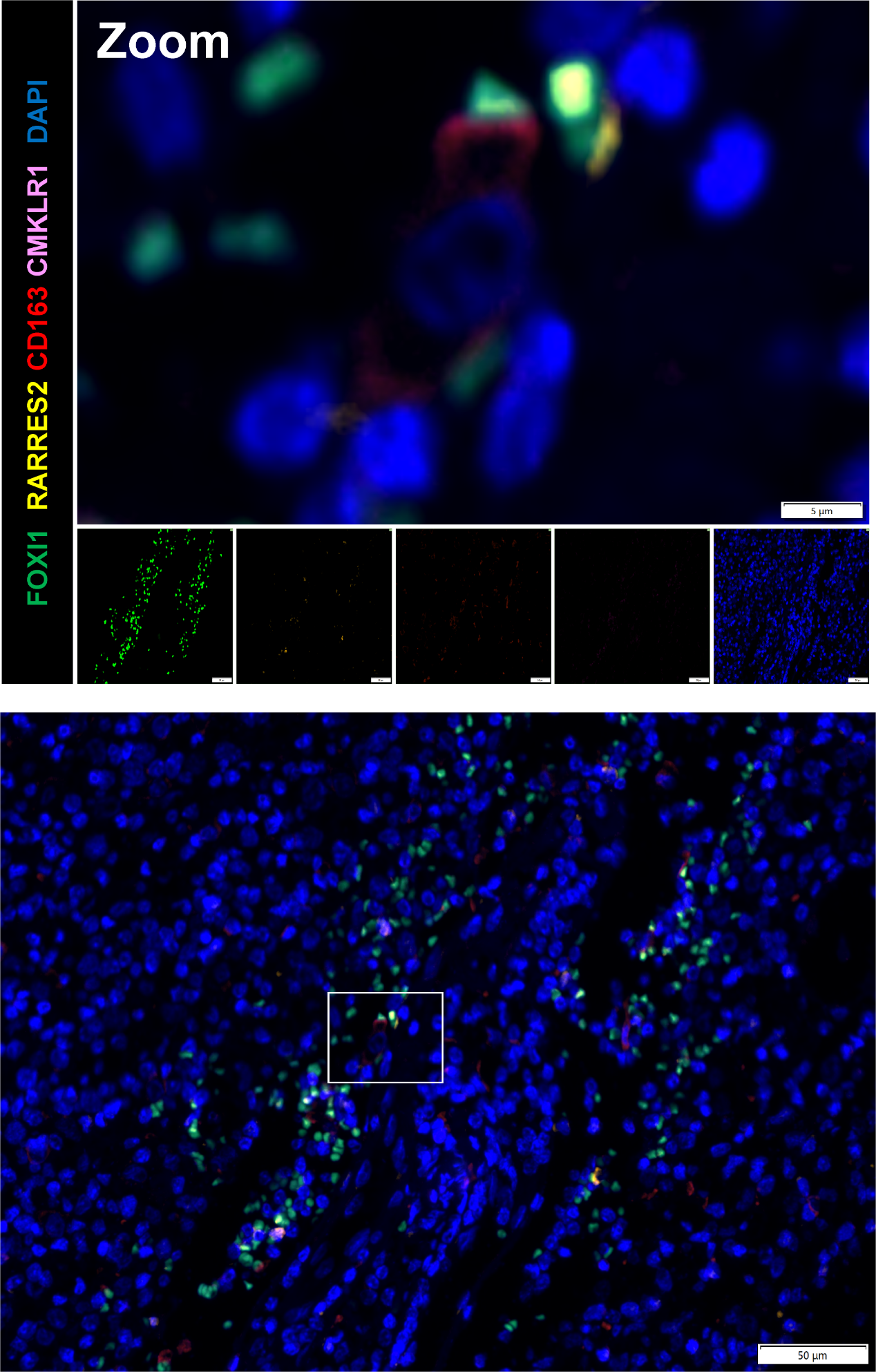
**

**High-resolution mIF image for Figure 6h.**
